# Assembly of two functionally-distinct protein import complexes in the outer membrane of plant chloroplasts

**DOI:** 10.1038/s41467-026-71676-6

**Published:** 2026-04-20

**Authors:** Sreedhar Nellaepalli, Domagoj Baretić, Astrid F. Brandner, Sybille Kubis-Waller, Duorong Xu, Ziad Soufi, Shuyang Cheng, Sireesha Kodru, Jun Fang, Úrsula Flores-Pérez, Vaishnavi Ravikumar, Pablo Pulido, Marjorie Fournier, Ivan Ahel, Syma Khalid, R. Paul Jarvis

**Affiliations:** 1 Section of Molecular Plant Biology, Department of Biology, University of Oxford, Oxford, UK; 2 Sir William Dunn School of Pathology, University of Oxford, Oxford, UK; 3 Department of Biochemistry, University of Oxford, Oxford, UK; 4 Department of Biology, University of Leicester, Leicester, UK; 5 Department of Plant Molecular Genetics, Centro Nacional de Biotecnologia, Consejo Superior de Investigaciones Cientificas (CNB-CSIC), Madrid, Spain; 6 Present Address: MRC Laboratory of Molecular Biology, Cambridge, UK

**Keywords:** Chloroplasts, Protein trafficking in plants, Protein translocation, Chloroplasts

## Abstract

The TOC translocon delivers thousands of nucleus-encoded proteins to chloroplasts and related non-photosynthetic plastids. It comprises the β-barrel channel, Toc75, and multiple isoforms of receptor GTPases, Toc33 and Toc159. However, exactly how TOC complexes are assembled in different plastid types is unknown. Here, we present detailed characterisation of two distinct TOC complexes, TOC-P and TOC-N, from photosynthetic chloroplasts and non-photosynthetic plastids, respectively. The assembled complexes are distinguished by having different sets of receptors, but both possess Toc75 which we identify as a central hub in TOC biogenesis: assembly is driven by *TOC75* expression, with Toc33 and Toc159 being added sequentially thereafter. Integrative structural analysis reveals a modular architecture for TOC-P comprising a cytosolic GTPase receptor module linked flexibly to a membrane β-barrel channel module. TOC-N has a similar overall architecture, albeit with some clear differences that likely account for observed functional differences related to client specificity.

## Abstract (web summary)

Chloroplasts import thousands of nucleus-encoded proteins via multiprotein translocons. The authors elucidate the assembly and molecular architecture of the outer-membrane TOC translocon (the import “entry gate”), which exists in two functionally-distinct forms, TOC-P and TOC-N.

## Introduction

Chloroplasts are membrane-bound endosymbiotic organelles in plants and algae that perform photosynthesis, the energetic basis for essentially all life on Earth^1^. They belong to a broader family of interconvertible organelles called the plastids, along with several non-photosynthetic variants (e.g., etioplasts in dark-grown plants, and leucoplasts in roots)^2^. Chloroplasts and other plastids are built up from thousands of different proteins, and because these are mostly nucleus-encoded, organelle biogenesis requires the efficient operation of sophisticated protein-import machines^3–6^. This machinery enables the massive, rapid delivery of thousands of different proteins to developing chloroplasts during photosynthetic establishment.

In plants, the plastid protein import machinery comprises two multiprotein complexes called TOC and TIC (translocon of the outer/inner chloroplast envelope membrane). TOC recognises and imports preproteins from the cytosol to the intermembrane space (IMS), while TIC completes delivery to the stroma in coordination with import-motor systems^3–6^. Because TOC governs which proteins enter the chloroplast, it is the crucial gatekeeper of the import machinery. The TOC comprises a channel protein, Toc75, and two preprotein-receptors, Toc33 and Toc159. As an Omp85-superfamily member (related to proteins in bacteria and mitochondria), Toc75 comprises a β-barrel domain that forms a preprotein-conducting channel, and a polypeptide-transport-associated (POTRA) domain that chaperones preproteins through the IMS^7^. The receptors possess related GTPase domains facing the cytosol^3^. Toc33 is structurally simple with a single α-helical membrane-span at its C-terminus, while Toc159 is more complex with an N-terminal acidic (A) domain and a large C-terminal membrane domain either side of the central GTPase domain.

In *Arabidopsis thaliana* and other plants, the receptors exist in multiple isoforms with different properties, forming two families (i.e., Toc159, Toc132, Toc120; and Toc33, Toc34)^3,8–10^. Toc159 and Toc33 are preferentially associated with photosynthetic development in green tissues, whereas the other isoforms, Toc132, Toc120 and Toc34, are more prominent in non-photosynthetic tissues, such as roots, and may help to maintain basic organelle functions^1^. Accordingly, plant knockout (ko) mutants of Toc33 (*plastid protein import 1*, *ppi1*) and Toc159 (*ppi2*) exhibit especially pronounced effects on chloroplast biogenesis^11–15^. Existence of these different receptor types may enable nuanced regulation of protein import to dynamically control the organelle’s proteome and functions, potentially circumventing damaging competition effects among the precursors. It may also facilitate to the differentiation of photosynthetic and non-photosynthetic plastid types.

Although the composition of the TOC apparatus is generally well established, the structural characteristics of the complexes and their differences between plastid types, as well as the molecular details of the relevant assembly processes, are poorly understood^16–18^. Here, we have addressed these knowledge gaps by studying different TOC configurations present in chloroplasts and non-green plastids. Using a combination of structural analysis following affinity purification and modelling, together with crosslinking, genetic, and in vivo labelling approaches, we elucidate the structural organisation, assembly and functions of two distinct, spatially enriched TOC complexes, and identify Toc75 as a central hub or “master player” in the biogenesis of TOC complexes.

## Results

### Isolation of two distinct TOC complexes from different plastid types

**Fig. 1:**
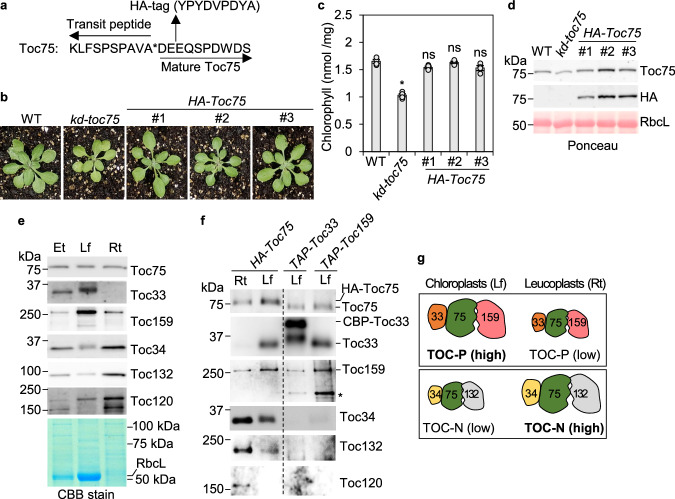
Isolation of two distinct TOC complexes, TOC-P and TOC-N. **a** Insertion site of the HA-tag in Toc75 of the *HA-Toc75* transgenic lines (E142-HA-E143); D141 is the N-terminus of mature Toc75 after transit peptide (TP) cleavage. Asterisk indicates the TP cleavage site. **b** Visible phenotypes of 4-week-old WT, *kd-toc75* and three independent *HA-Toc75* transgenic lines (#1-3). **c** Chlorophyll concentrations in 4-week-old plants of the genotypes shown in (**b**). Asterisks indicate significance according to paired two-tailed Student’s t tests comparing the mutant genotypes with WT (*p < 0.001; ns, not significant). All values are means ± SEM (*n* = 4 experiments). **d** Immunoblotting analysis of total protein extracts from 12–14-day-old plants of the genotypes shown in (**b**). Samples were loaded based on equal plant fresh weight. **e** Immunoblotting analysis of protein extracts from dark-grown wild-type seedlings containing etioplasts (Et); from chloroplast-enriched leaf fractions (Lf); and from roots containing leucoplasts (Rt). The samples were prepared from 7-day-old (Et) or 14-day-old (Lf, Rt) plants. Samples were normalised based on equal Toc75. The experiment was performed three times with similar results. **f** Isolation of TOC complexes by HA- or TAP-tag affinity purification from Lf or Rt samples (similar to those in **e**) from the indicated transgenic lines. Samples were solubilised with 1% β-DM before affinity purification. Purified samples were analysed by immunoblotting, with loading normalised to achieve comparable Toc75 levels. *HA-Toc75* line #1 was used for the purifications. Asterisk indicates a proteolytic fragment of Toc159. The experiment was performed three times with similar results. **g** Schematic representation of the TOC-P and TOC-N complexes that are enriched in chloroplasts and leucoplasts, respectively.

To enable analysis of TOC composition in different plastid types, we generated *A. thaliana* transgenic plants expressing Toc75 with an HA epitope tag inserted close to the N-terminus of the mature protein, between amino acid residues E142 and E143 of the precursor (E142-HA-E143); this was done in a *toc75* knockdown (kd) mutant background (i.e., *mar1/toc75-III-3*, bearing a G658R missense mutation) which accumulates Toc75 to just ~ 30% of the normal level^19^ (Fig. 1a). Three independent *HA-Toc75* transgenic lines showed recovery to the wild-type green phenotype (Fig. 1b, c), linked to restored Toc75 protein accumulation (Fig. 1d). This indicated that insertion of the HA-tag at E142, which is close to the N-terminal linker (148–172) that caps the POTRA1 domain^7^, did not affect the function of Toc75.

Initially, we determined the abundance of TOC components in different tissues of the wild type by immunoblotting. Receptor isoforms Toc33 and Toc159 were enriched in leaves (in chloroplasts), whereas Toc34 and Toc132/120 (Toc132 and Toc120 are highly similar and considered to be largely or completely redundant^15^) were enriched in roots and petals (in non-green plastids or leucoplasts), revealing clear differentiation of TOC composition in different plastid types (Fig. 1e, Supplementary Fig. 1). Interestingly, all these TOC components were also detected in etiolated seedlings, which contain partially differentiated plastids, indicating pre-existence of the TOC components before establishment of photosynthesis.

Next, we applied anti-HA affinity chromatography to purify TOC complexes from different tissues of the *HA-Toc75* transgenic plants^20^. Polypeptides in the HA-Toc75 purification from leaf chloroplasts were visualised by silver staining and subsequently analysed by mass-spectrometry (Supplementary Fig. 2, Supplementary Data 1). Furthermore, the HA-Toc75 purifications from leaf chloroplasts and root leucoplasts were compared by immunoblotting analysis (Fig. 1f). Overall, the analysis showed that Toc75 is mainly associated with Toc33 and Toc159 in leaf chloroplasts, and to a lesser extent with Toc34, Toc132 and Toc120. In contrast, Toc75 is mainly associated with Toc34, Toc132 and Toc120 in root leucoplasts, and to a lesser extent with Toc33 and Toc159 (as judged by comparing with the corresponding interactions in leaf chloroplasts) (Fig. 1f, left side). These data are consistent with the aforementioned TOC accumulation biases (Fig. 1e) and point to the predominance of distinct TOC complexes in leaf chloroplasts and root leucoplasts, although clearly the prevalence of one type does not preclude co-existence of the other at low levels. For clarity, we herein refer to the Toc33-75-159 configuration as TOC-P (for photosynthetic) and the Toc34-75-132/120 configuration as TOC-N (for non-photosynthetic) (Fig. 1g).

We wished to determine if TOC-P and TOC-N interact in chloroplast membranes. For this purpose, we used transgenic lines expressing tandem-affinity-purification (TAP)-tagged Toc33 and Toc159 (*TAP-Toc33* and *TAP-Toc159*)^21,22^ to affinity purify TAP-Toc33 and TAP-Toc159 complexes, respectively, from chloroplast extracts, comparing the results with HA-Toc75 purifications (Fig. 1f, right side). In the resulting TAP samples, co-purification of Toc34 and Toc132 was negligible when compared to HA-Toc75 purifications. In contrast, co-purification of Toc33 and Toc159 in the TAP purifications was very similar to that in HA-Toc75 purifications (Fig. 1f, left side). This indicated that TOC-P and TOC-N exist as distinct complexes and that Toc75 is the common factor of both complexes.

### Toc75 and Toc33/34 maintain the stability of TOC complexes

**Fig. 2:**
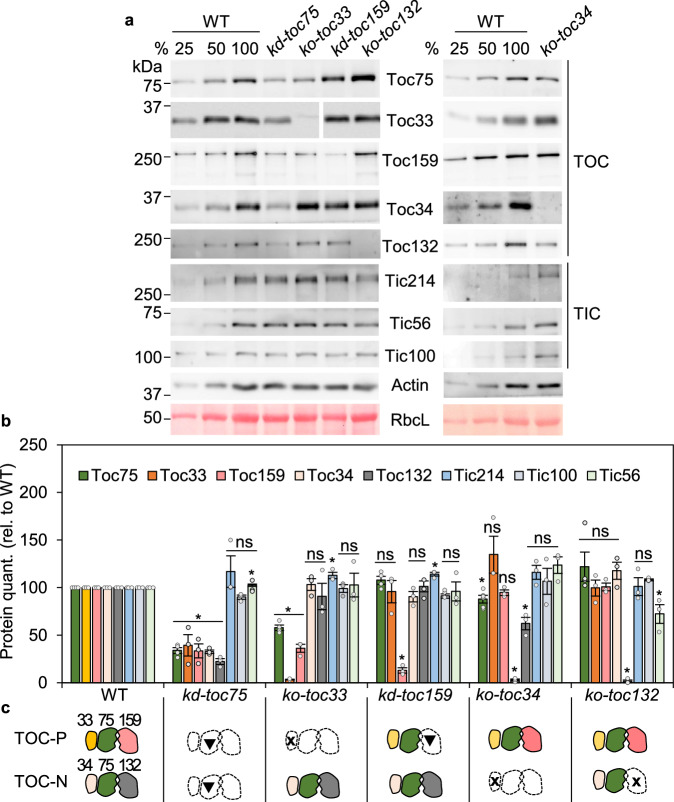
Comparison of the contributions of different TOC components to TOC complex stability. **a** Immunoblotting analysis of leaf protein extracts from 12-14-day-old WT, *kd-toc75* (*mar1/toc75-III-3*), *ko-toc33* (*ppi1-1*), *kd-toc159* (*fts1/ppi2-3*), *ko-toc132* (*toc132-2*), and *ko-toc34* (*ppi3-2*) plants. Samples were loaded based on equal plant fresh weight. Percentage values (25–100) indicate different loadings according to the fresh weight of tissue. **b** Protein quantification of the immunoblots in (**a**) and of other similar results. All values are means ± SEM (*n* = 3-4 experiments). Asterisks indicate significance according to paired one-tailed Student’s *t* tests comparing the mutant genotypes with WT (*p < 0.02; ns, not significant). **c** Schematic representation of the results in (**a**) and (**b**). Dotted lines indicate reduced accumulation of the relevant component. Components marked x are affected by a knockout (ko) mutation; components marked with a triangle are affected by a knockdown (kd) mutation.

To elucidate which TOC components are important in stabilising the integrity of the TOC complexes, we analysed the accumulation of all TOC components in the following *Arabidopsis* TOC mutant genotypes: *mar1/toc75-III-3* (*kd-toc75*)^23,24^, *ppi1-1* (*ko*-*toc33*)^14^, *ppi3-2* (*ko-toc34*)^12^, *fts1/ppi2-3* (*kd-toc159*)^21,25^, and *toc132-2* (*ko-toc132*)^15^ (Fig. 2). As previously described, these mutants typically display a characteristic chlorotic phenotype (Supplementary Fig. 3).

As reported previously^19^ and shown earlier (Fig. 1b), the *kd-toc75* mutant displayed a ~ 70% reduction in Toc75 accumulation (Fig. 2a, b). In this mutant, the Toc33/Toc34 and Toc159/Toc132 receptor isoforms were all similarly reduced, revealing strong effects on the integrity of both TOC-P and TOC-N. In contrast, in the *ko-toc33* mutant, Toc75 and Toc159 were both reduced by ~ 50%, but the components of TOC-N (Toc34 and Toc132) were unaffected. This implies that TOC-P is specifically disrupted in *ko-toc33*. Similarly, in the *ko-toc34* mutant, Toc132 was the most strongly affected protein amongst the other TOC components, indicating a specific effect on TOC-N. Interestingly, in the *kd-toc159* and *ko-toc132* mutants, the Toc75, Toc33 and Toc34 proteins were not affected. Thus, we hypothesise that Toc159 and Toc132 are not required for the stability of the other components of TOC-P and TOC-N, respectively, and that in the aforementioned mutants, these other components maintain their interactions (as in Fig. 1f, g). In general, TIC components were largely unaffected in the different *toc* mutants (Fig. 2a, b). In summary, these results suggest that Toc75 and Toc33/34 are the key players in maintaining the integrity of the TOC-P and TOC-N complexes (Fig. 2c).

Importantly, qRT-PCR analysis revealed that *TOC* gene expression changes in the *toc* mutants were minimal, and generally positive (not negative) (Supplementary Fig. 4). This was the case even in the *kd-toc75* and *ko-toc33* mutants which displayed the strongest effects on TOC protein accumulation. This supports the view that the observed protein effects (summarised in Fig. 2c) were due to defects at the post-translational level.

### Integrative structural analysis reveals a bipartite structure for TOC-P

**Fig. 3:**
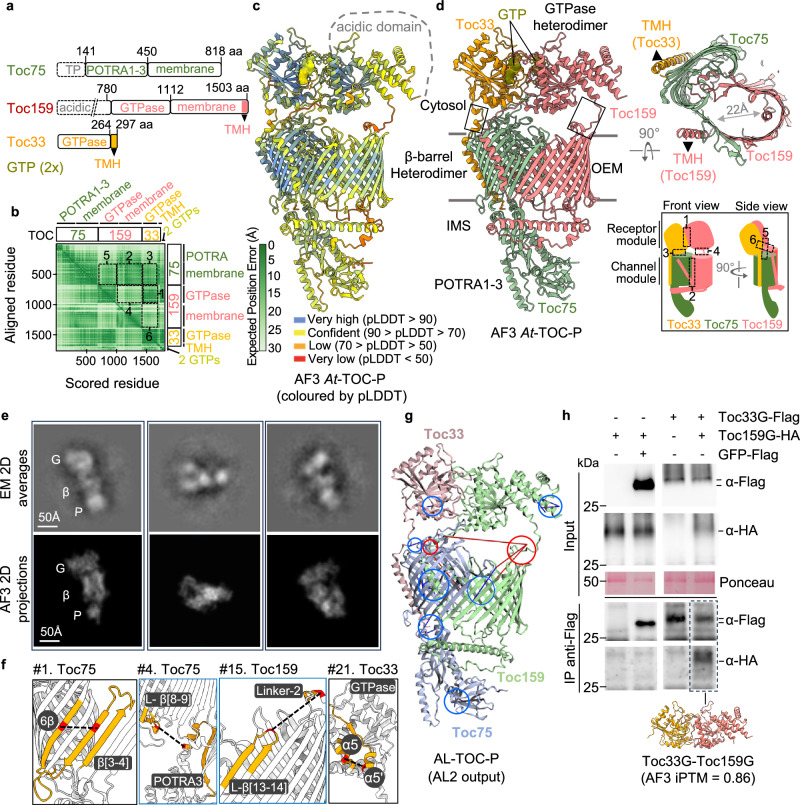
Integrative structural analysis of TOC-P reveals a heterodimeric-GTPase–heterodimeric-β-barrel architecture. **a** Components and ligands (two GTPs) of the TOC-P complex submitted to the AF3 server. Domain maps of the inputted protein sequences are shown; the sequences of Toc75 and Toc159 were N-terminally truncated to remove transit peptide and disordered acidic A-domain sequences, respectively. The numbers refer to amino acid (aa) coordinates of the full-length proteins. **b** Predicted alignment error (PAE) plot from the AF3 analysis of the components in (**a**). This 2D plot shows the expected position error (Å) of different domains and interfaces, and measures the expected positional error (confidence) of pairs of positions running along the axes. Dark green indicates lower error, while light green indicates higher error. Dashed boxes 1–6 indicate interfaces between the TOC subunits as follows: 1, Toc33-GTPase:Toc159-GTPase; 2, Toc75-membrane:Toc159-membrane; 3, Toc33-GTPase:Toc75-membrane; 4, Toc159-GTPase:Toc159-membrane; 5, Toc75-membrane:Toc159-GTPase; 6, Toc159-membrane:Toc33-GTPase. These interfaces are also marked in the inset in (**d**). **c**, **d** Structure of the TOC-P complex as modelled by AF3. The polypeptide chains are coloured according to pLDDT scores (**c**) or protein identity (**d**; front and top views are shown). The position of the acidic domain of Toc159 (which was excluded from the analysis because of its intrinsically disordered nature) is shown by the dashed line. Two linkers connecting the GTPase heterodimer with the β-barrel heterodimer are identified using rectangles. In the top view (90° rotation relative to the front view), the membrane domain of TOC-P, composed of a Toc75-Toc159 hybrid channel, is clearly shown. Transmembrane helices (TMH) of Toc33 and Toc159 are also shown. Inset: Simplified front and side view representations of the TOC-P structure shown. **e** Comparison of 2D back projections of the AF3-derived TOC-P structure shown in (**c**, **d**) (generated by CryoSPARC) (bottom) with similar 2D class averages from the negative-staining EM analysis of purified TOC-P complex (top). **f** XL-MS analysis of the TOC-P complex identified multiple crosslinks. The affinity-purified complex was crosslinked with BS3 and trypsin digested prior to MS analysis. For the full list of identified peptides, refer to Supplementary Data 2. Example crosslinks in Toc75 (#1, #4), Toc159 (#15), and Toc33 (#21) are shown, mapped onto the structure. In each image, crosslinked parts of the structure are highlighted (yellow for peptides, red for residues), with the crosslinks shown by the dotted black lines. Linker-2 connects the GTPase and β-barrel domains of Toc159. L, loop. **g** Structure of TOC-P as predicted by AlphaLink2 (AL-TOC-P). The AlphaLink2 prediction satisfied the majority of experimentally detected crosslinks (17 in total, marked with blue circles), indicating good agreement between experimental data and predicted structures. A few crosslinks (4, marked with red circles) could not be captured by a single static model (Cα-Cα distances of 39–55 Å), suggesting they were located in flexible regions of the complex. **h** Co-immunoprecipitation analysis of the Toc159 and Toc33 GTPase (G) domains. Toc159G-HA and Toc33G-Flag were transiently expressed in *Nicotiana benthamiana* plants, and anti-Flag immunoprecipitation (IP) was performed. Input and IP samples were analysed by immunoblotting using anti-Flag (α-Flag) and anti-HA (α-HA) antibodies. GFP-Flag was used as a negative control upon co-expression with Toc159G-HA.

To gain insight into the structural basis for our biochemical observations (Figs. 1, 2), we implemented an integrative structural analysis. First, a computational approach using AlphaFold3 (AF3) was applied to analyse the TOC-P complex^26^. The amino acid sequences of Toc75 (without the transit peptide), Toc33, and Toc159 (without the unstructured A domain^27^) from *A. thaliana*, plus two GTP ligands, were submitted to the AF3 server to generate structural predictions (Fig. 3a); the sequence of the Toc159 A domain was removed as its intrinsically disordered nature poses a challenge for AF3 prediction^27^. We then analysed the top-scoring TOC model to elucidate domain organisation and component interactions within the complex.

AF3 predicted the overall organisation of the TOC-P complex with good confidence (ipTM = 0.72; pTM = 0.74), and subcomplexes of TOC-P with even higher confidence (ipTM = 0.83–0.86) (Fig. 3b and Supplementary Fig. 5). The model revealed a striking heterodimeric-GTPase–heterodimeric-β-barrel structure, as follows: the cytosolic GTPase domains of Toc33 and Toc159 form a heterodimeric receptor module connected by two linkers to an integrated membrane channel module, the latter composed primarily of the β-barrel segments of Toc75 and Toc159 which together form a hybrid barrel; Toc75 further extends a globular domain comprising three POTRA domains into the presumptive IMS. Overall, TOC-P forms a bipartite receptor-channel complex with a novel arrangement (Fig. 3c, d).

To validate the presented TOC-P model, we performed negative-staining analysis of purified TOC-P complex by electron microscopy (EM) (Fig. 3e) and crosslinking mass spectrometry (XL-MS) (Fig. 3f). For the former, TOC-P complex was isolated from chloroplast membrane extracts of the *HA-Toc75* transgenic line (Fig. 1) by affinity purification and size-exclusive chromatography, and then negatively stained prior to EM imaging and 2D classification of the images. The results revealed that purified TOC-P single particles have a very similar modular arrangement to that seen in the TOC-P AF3 model, as revealed by comparison with 2D projections of the AF3 model (Fig. 3e and Supplementary Fig. 6a).

For the XL-MS analysis, similar affinity-purified TOC-P samples were chemically crosslinked using bis(sulfosuccinimidyl) suberate (BS3) prior to proteolytic digestion for MS analysis. This identified several crosslinks (intra-domain and inter-domain) within Toc75, Toc159 and Toc33, and the positioning of most of these crosslinks was accordant with the AF3 model as the relevant residues were all in close proximity within the structure (Fig. 3f, Supplementary Fig. 6b, c and Supplementary Data 2). Notably, crosslinks between the POTRA and β-barrel domains of Toc75 (crosslinks #4 and #9), and between the central linker and β-barrel domain of Toc159 (crosslinks #15 and #20), confirmed the close proximity between these elements of the structure (Fig. 3f). A few crosslinks were identified in unstructured regions with low pLDDT scores (three in Toc159 [#15, #16, #20], one in Toc75 [#13]) which, according to the AF3 prediction, join residues that are more distantly localised (39.8–55.8 Å) (Supplementary Fig. 6b, c). These likely reflect structural flexibility or a dynamic nature of the complex.

Next, we implemented AlphaLink2 to integrate our crosslinking data with AlphaFold prediction of the TOC-P complex (Fig. 3g)^28,29^. We found that the AlphaLink2 TOC-P model (AL-TOC-P) satisfied the majority of the experimentally-detected crosslinks, indicating good agreement between experimental data and the predicted structure (Supplementary Fig. 6d). Moreover, to validate the formation of the receptor module, we performed co-immunoprecipitation (co-IP) assays using a plant transient expression system to assess interactions between the two TOC-P GTPase domains (Toc33G and Toc159G); the two domains were tagged (Toc33G-Flag, Toc159G-HA) to enable purification and detection (Fig. 3h). The results revealed physical interaction between the two GTPases in planta, consistent with the receptor module part of the TOC-P structure.

In summary, these EM, XL-MS and co-IP results support the veracity of the TOC-P AF3 model. Such an integrative approach, combining computational modelling with experimental validation as described, may present significant advantages over solely cryo-EM-focused approaches when analysing complexes with flexible domains such as the TOC complex (see below)^30,31^.

### AF3 analysis reveals a similar structure for TOC-N

Next, we performed a similar analysis of the TOC-N complex by AF3, which also resulted in a good-confidence model (ipTM = 0.70; pTM = 0.72) (Supplementary Fig. 7a, b). As might be expected given its functional similarity, TOC-N was found to share similar structural features with TOC-P (i.e., a heterodimeric-GTPase–heterodimeric-β-barrel arrangement). However, the two complexes did exhibit some notable differences, including an extended IMS helix in Toc34 (Supplementary Fig. 7b), a disordered α5’ region in Toc34 (Supplementary Fig. 7c), and several areas of contrasting surface electrostatic potential with acidic and basic regions (Supplementary Fig. 7f). The TOC-N complex exhibited a weaker gradation of surface electrostatic potential between the receptor and channel modules by comparison with TOC-P. Such differences could be partly responsible for the efficient routing of different preproteins; e.g., they may facilitate the heavy traffic of photosynthetic preprotein import via TOC-P, while a slower import process may be more suitable or adequate for housekeeping preprotein import via TOC-N. Although the A-domains of Toc159 (aa 1–786) and Toc132 (aa 1–485) were not analysed here, because their intrinsically disordered nature presents problems for structural analysis, these domains may also be important in defining client specificities^13,27^.

### The TOC-P structure incorporates modular dynamics and intimate Toc75 contacts supporting structural integrity

**Fig. 4:**
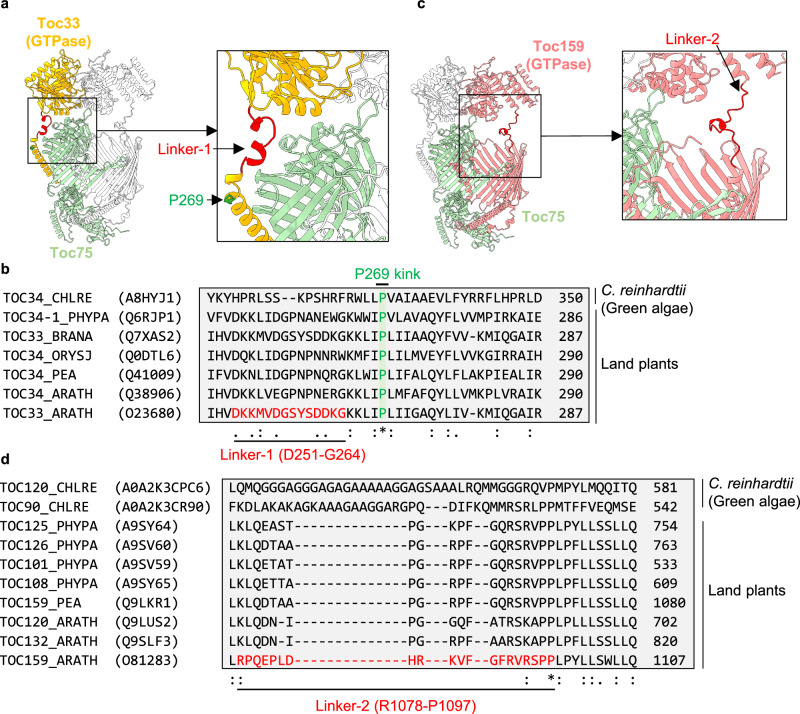
Coupling of the GTPase heterodimer with the β-barrel heterodimer is via two linkers. **a**,** c** Elevated front views of the TOC-P structure showing the positions of linker-1 (**a**) and linker-2 (**c**). Linker-1 (D251-G264 of Toc33) positions the Toc33 GTPase domain at the cytosolic face of the β-barrel domain of Toc75. Linker-2 (R1078-P1097 of Toc159) positions the Toc159 GTPase domain close to the β-barrel domain of Toc159. The inset in each case shows the linker at higher magnification; a proline kink (P269) at the end of the Toc33 TMH is highlighted (**a, inset**). **b**, **d** Multiple sequence alignments (MSAs) showing conservation of the two linkers. Toc33-related sequences were obtained from UniProt for *C. reinhardtii* (A8HYJ1, TOC34_CHLRE), *P. patens* (Q6RJP, TOC34-1_PHYPA), *A. thaliana* (O23680, TOC33_ATHAL; Q38906, TOC34_ATHAL), *Brassica napus* (Q7XAS2, TOC33_BRANA), *O. sativa* (Q0DTL6, TOC34_ORYSJ), and *P. sativum* (Q41009, TOC34_PEA) (**b**). Toc159-related sequences were obtained from UniProt for *C. reinhardtii* (A0A2K3CPC6, TOC120_CHLRE; A0A2K3CR90, TOC90_CHLRE), *A. thaliana* (O81283, TOC159_ARATH; Q9LUS2, TOC120_ARATH; Q9SLF3, TOC132_ARATH), *P. patens* (A9SY64, TOC125_PHYPA; A9SV60, TOC126_PHYPA; A9SV59, TOC101_PHYPA; A9SY65, TOC108_PHYPA) (**d**). Complete sequences were aligned in each case, but for simplicity, only linker-proximal regions are shown here. Clustal Omega was used to perform both MSAs^94^, and the relevant regions were copied out manually. The sequences of the linkers in *Arabidopsis* Toc33 and Toc159 are highlighted (in red), as is the position corresponding to the conserved Toc33 P269 residue (kink; in green). Coordinates at right show positions in each protein sequence; symbols at the bottom (*:.) indicate the degree of conservation at each position. From (**d**) it is evident that in land plants linker-2 is reasonably well conserved, but in green algae it contains sizeable alanine-rich insertions.

To shed light on translocon structure and function, we further scrutinised the TOC-P structural model. Within the GTPase heterodimer, the two receptor components are positioned around a pseudo two-fold rotational axis relative to each other (Supplementary Fig. 8a). Two tight GTP binding pockets exist at the Toc33-Toc159 interface for which the prediction confidence is high (Fig. 3c, d, Supplementary Figs. 8, 9 and Supplementary Results). As already noted, the GTPase heterodimer is connected to the top of the hybrid β-barrel with two linkers (Fig. 3c, d). Linker-1 (Toc33, D251-G264) connects the GTPase domain and single transmembrane helix (TMH) of Toc33, the latter ending adjacent to the cytosolic face of the Toc75 β-barrel domain (Fig. 4a, b); notably, a highly-conserved proline kink (P269) at the cytosolic end of the Toc33 TMH may help in the orientation of the linker and GTPase domain towards the channel. Linker-2 (Toc159, R1078-P1097) brings the GTPase and membrane domains of Toc159 into close proximity (Fig. 4c, d).

**Fig. 5:**
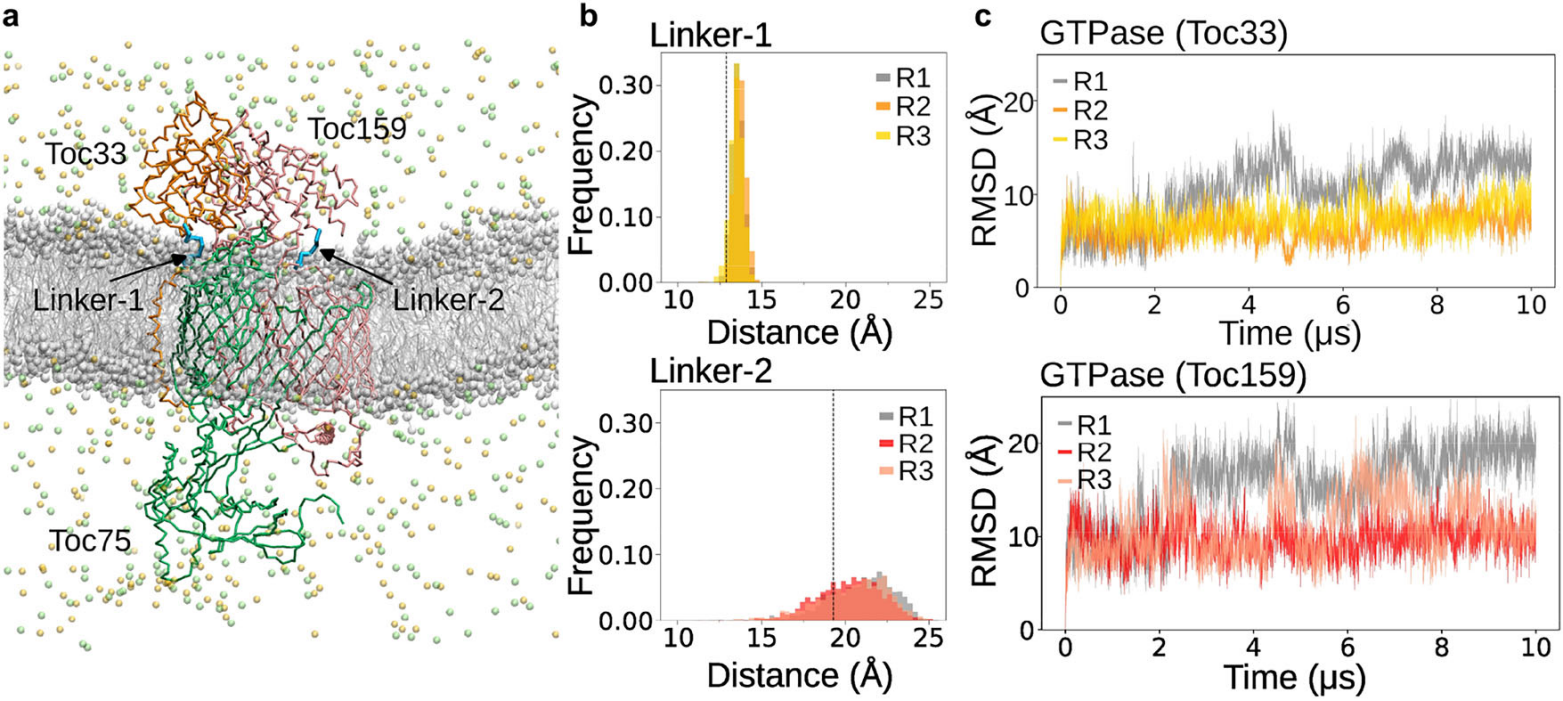
Molecular dynamics simulation analysis of the TOC-P complex. **a** Setup for the molecular dynamics (MD) simulation analysis of TOC-P. The complex from Fig. 3c, d was embedded in a symmetric 1-palmitoyl-2-oleoyl-*sn*-glycero-3-phosphocholine (POPC) lipid bilayer. The protein chains are coloured as in Fig. 3d; POPC molecules are shown in light grey with their headgroup moieties (phosphate and choline groups) represented as spheres; and Cl^-^ and Na^+^ ions are shown as green and orange spheres, respectively. Water is omitted for clarity. Three independent simulation runs (R1–R3) were performed. **b** Flexibility of linker-1 and linker-2 in the MD analysis. Histograms showing the end-to-end distance distribution during the full production run for all three independent replicates. The dotted vertical line in each case corresponds to the value of the initial AF3 model. **c** Mobility of the GTPase domains of Toc33 and Toc159 in the MD analysis. RMSD plots of the GTPase domains after fitting on the transmembrane region of the TOC-P complex, for all three independent runs.

To analyse the flexibility of these linkers, we performed molecular dynamics (MD) simulations of the TOC complex embedded in a 1-palmitoyl-2-oleoyl-*sn*-glycero-3-phosphocholine (POPC) lipid bilayer (Fig. 5a). The results revealed that linker-2 is more flexible than linker-1 (Supplementary Movies 1, 2 and Fig. 5b, c). As a consequence, the GTPase domain of Toc159 is more mobile than the GTPase domain of Toc33, showing larger root mean square deviation (RMSD) values (Supplementary Movies 1, 2 and Fig. 5b, c). Movement of the Toc159 GTPase domain is likely influenced by its α−2 helix through the establishment of spontaneous membrane contacts (Supplementary Fig. 10). Overall, this dynamic behaviour, which does not occlude the channel entry gate at the cytosolic side, may be relevant for preprotein handover from the receptor module to the channel module.

Another remarkable feature of the structure is that the membrane domain of Toc75 and the membrane domain of Toc159 (consisting of the β-barrel and two embedded segments) cooperate in the formation of a hybrid β-barrel comprising a total of 30 antiparallel β-strands: a 16-strand β-sheet from Toc75, and a 14-strand β-sheet from Toc159 (Fig. 3d and Supplementary Fig. 11a–e). The two constituent β-sheet domains are joined by a β-seam, whereby the first β-strand (β1) of Toc75 is bonded to the last β-strand (β14) of Toc159 (Fig. 3d and Supplementary Fig. 11c). These interactions create a flat face on one side of the β-barrel. The C-terminal transmembrane helix of Toc159 traverses the outer face of the β-seam, likely stabilising the β-barrel. On the opposite side of the barrel, the β-sheet domains of Toc75 (β13-16) and Toc159 (β1-4) converge and  curl inwards to close off the β-barrel with weak van der Waals interactions, forming a hydrophobic groove (Supplementary Fig. 11d–f). This resembles the hybrid β-barrel of a BamA-substrate complex (Supplementary Fig. 11g)^30,32^. In addition, embedded segment-II of Toc159 extends across this junction and underneath the Toc75-side of the β-barrel (Supplementary Fig. 11c, d). This overall arrangement of the hybrid β-barrel may allow the protein import channel to adapt dynamically in order to accommodate folded/unfolded protein domains of different sizes^30,33–35^.

The Toc75 part of the dimeric β-barrel establishes contacts with the TMH of Toc33 and the TMH of Toc159 on different sides (Fig. 3c, d and Supplementary Fig. 11f). Moreover, interface analysis of TOC-P identified Toc75 as a hub for interactions with Toc33 both in the membrane region and at the cytosolic side, and with Toc159 mainly at the membrane level (Supplementary Fig. 12a, b). Indeed, a missense mutation (G518R of mature Toc75) in *kd-toc75* (*mar1*) affecting a conserved position that is proximal to these Toc75 contact sites destabilises the TOC-P and TOC-N complexes (Fig. 2 and Supplementary Fig. 12a, d), supporting their importance for complex integrity. In contrast, Toc33-Toc159 interactions are limited to the GTPase domains (Fig. 3c, d and Supplementary Fig. 12c). While Toc33 is important for the stabilisation of the holocomplex (most likely through its TMH and GTPase domains) (Fig. 2c), it appears that the TMH of Toc159 is not. This is clear because the *kd-toc159* (*fts1*) mutant (which lacks the C-terminal TMH due to a premature stop at codon 1472) displays a drastic reduction in Toc159 accumulation without impacting levels of Toc75 and Toc33 accumulation (Fig. 2c and Supplementary Fig. 13a). However, Toc159 in *kd-toc159* but not that in wild type is destabilised by alkaline treatment of a membrane fraction, suggesting that the missing TMH is needed to properly anchor and stabilise the Toc159 β-barrel in the membrane (Supplementary Fig. 13b). Recently, it was shown that this TMH of Toc159 is also important in its membrane targeting^36^, and thus lack of this region likely causes defects in the TOC assembly process.

### Conserved and distinct features of the TOC complex in plants and algae

**Fig. 6:**
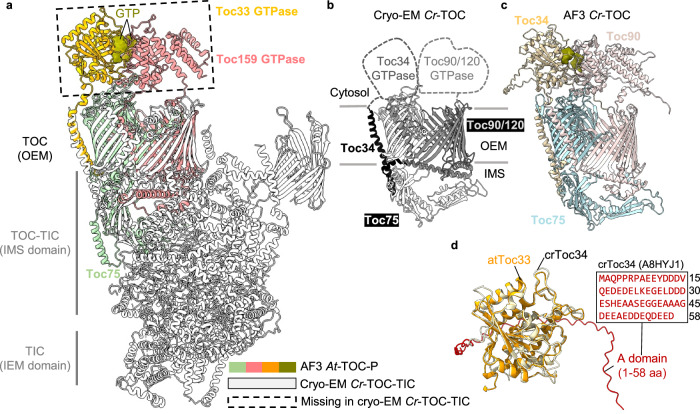
Comparison of the Arabidopsis TOC-P AF3 structure with an algal TOC-TIC cryo-EM structure. **a** Superposition (front view) of the AF3-generated TOC-P complex (coloured) with a cryo-EM structure of the *C. reinhardtii* TOC-TIC supercomplex (grey); just one of the published algal cryo-EM structures is used here (PDB: 7VCF) as these structures are very similar^30,31^. This analysis revealed similarities in the arrangement of the membrane-embedded β-barrel domains, but also some clear differences between the two structures. Most notably, while the GTPase domains of Toc33 and Toc159 are depicted with high confidence in the AF3 model of the *Arabidopsis* TOC-P complex, the GTPase domains of the algal receptors (termed Toc34 and Toc120/Toc90) were entirely missing in both of the cryo-EM structures^30,31^. **b** Cryo-EM structure of a TOC complex from *C. reinhardtii* (PDB: 7VCF). The absence of resolution regarding the cytosolic GTPase domains of the receptors (Toc34 and Toc90/120) is highlighted. **c** AF3-predicted structure of the same *C. reinhardtii* TOC complex, revealing similarity with the corresponding cryo-EM structure (compare with **b**), and showing that the arrangement of the GTPase domains is similar to that in Arabidopsis TOC-P (compare with **a**). **d** Overlay of AF3-predicted models for the *A. thaliana* Toc33 (atToc33) and *C. reinhardtii* Toc34 (crToc34) GTPase domains showing the unstructured acidic (A) domain of the latter (highlighted in red) (Supplementary Results).

Wishing to assess the extent to which the protein import apparatus has been structurally conserved during chloroplast evolution, we compared the AF3-predicted TOC-P complex with the cryo-EM structures of TOC-TIC supercomplexes from the green alga *Chlamydomonas reinhardtii*^30,31^ (Fig. 6a). The latter lack information on the cytosolic GTPase domains of the receptors (termed Toc34 and Toc120/Toc90) (Fig. 6a, b), presumably due to the flexibility of the linkers described earlier. Thus, we applied AF3 to model the algal TOC complex, revealing that the missing domains assume a heterodimeric arrangement analogous to that in plants (ipTM = 0.75; pTM = 0.74) (Fig. 6c). Comparing the different structures, we found that TOC-P from *A. thaliana* resembles the cryo-EM and AF3-predicted structures of the algal TOC complex, indicating that structural organisation of the TOC complex is broadly conserved in plants and green algae (Fig. 6), albeit with some distinct differences in the receptors (Supplementary Results).

Next, to investigate TOC-TIC interactions in plants, we separated protein complexes from leaf extracts of wild-type Arabidopsis by sucrose density gradient ultracentrifugation, analysing the resulting fractions by immunoblotting (Supplementary Fig. 14a, b). The results showed that TOC and TIC components primarily form distinct complexes of different sizes ( ~ 300 and ~ 600 kDa, respectively), suggesting that interactions between the TOC and TIC complexes in plants are weak or transient under these conditions, although minor co-fractionation of TOC components in the TIC-enriched heavier fractions (potentially representing TOC-TIC supercomplexes) was observed. We further evaluated this point by analysing affinity-purified TOC-P complexes from our *HA-Toc75* line, as well as complexes from TAP-tagged transgenic lines (*TAP-Toc159*, *TAP-Toc33*), by immunoblotting (Supplementary Fig. 14b, c); and quantitative mass-spectrometry analysis of the HA-Toc75 preparations was additionally performed (Supplementary Fig. 14d). The results reinforced the conclusion that there are weak but specific interactions between TOC and TIC components in plants, which contrasts notably with the heavily integrated TOC-TIC arrangement seen in the algal cryo-EM structures^30,31^. For example, in the *C. reinhardtii* structures, a small segment of Tic214 is inserted into the hybrid β-barrel of TOC, occupying an area that in our Arabidopsis TOC model is vacant (Fig. 3c, d). However, stronger TOC-TIC interactions in plants are likely to occur during active preprotein import, as reported^37–39^. Several studies based on chloroplast protein import assays previously suggested that proportions of plant TOC and TIC exist as TOC-TIC supercomplexes even in the absence of added preprotein^37,40–42^; we speculate that these were preexisting interactions formed in planta, before organelle isolation, by chloroplasts that were actively engaged in natural import processes.

Overall, these results reveal a remarkable distinction between the plant and algal TOC systems, with the latter being tightly integrated with the TIC apparatus in an interlocked TOC-TIC supercomplex^30,31^. This implies that the plant and algal protein import systems are substantially different.

### Toc75 is a key player in the biogenesis of TOC complexes

**Fig. 7:**
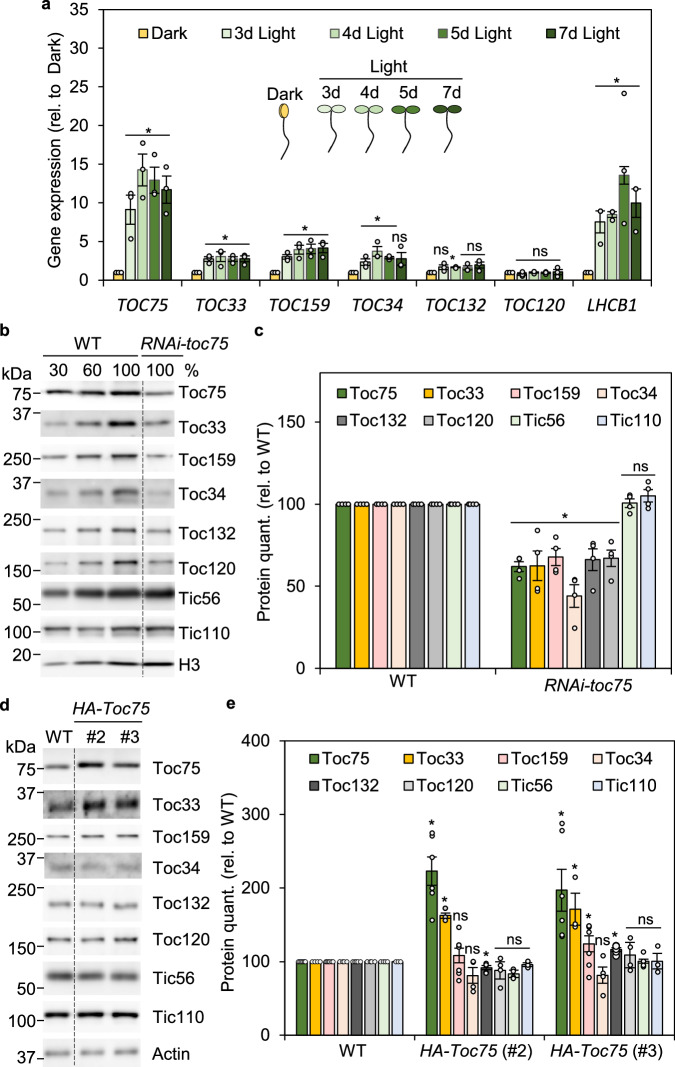
Identification of Toc75 as the central player in TOC complex formation. **a** Gene expression analysis of TOC components during de-etiolation by qRT-PCR. Dark-grown WT seedlings were exposed to long-day cycles (16 h light / 8 h dark) for between 3 and 7 days (3d-7d Light). Expression data for genes of interest were normalised using data for *ACTIN2*. Asterisks indicate significance according to paired one-tailed Student’s *t* tests comparing the de-etiolation time points with the Dark sample (*p < 0.05; ns, not significant). **b**,** c** Immunoblotting analysis of WT and *RNAi-toc75* knockdown plants (**b**), and corresponding protein quantification by densitometry (**c**). Seedlings were grown for 10–12 days on medium containing 50 mM dexamethasone inducer prior to analysis. In (**b**), percentage values indicate different loadings according to the fresh weight of tissue. **d**, **e** Immunoblotting analysis of WT and *HA-Toc75* overexpressor plants (**d**), and corresponding protein quantification by densitometry (**e**). In (**c**, **e**), asterisks indicate significance according to paired one-tailed Student’s *t* tests comparing *RNAi-toc75* or *HA-Toc75* with WT (*p < 0.042; ns, not significant). All values are means ± SEM (*n* = 3–6 experiments).

Our biochemical and structural analyses revealed differing contributions of the TOC components to the overall integrity and architecture of the complex. To investigate if these differences are also reflected in the assembly pathway, we studied the biogenesis of the TOC complex. We began by analysing the expression of *TOC* genes during de-etiolation – the greening process in which chloroplast biogenesis results in photosynthetic establishment^43–45^. Dark-grown wild-type seedlings were exposed to long-day photoperiods for 7 days, during which time photosynthesis was gradually initiated. During the first 5 days of illumination, expression of the light-harvesting complex gene *LHCB1* increased rapidly and by an order of magnitude, as expected (Fig. 7a). Interestingly, *TOC75* expression followed a very similar pattern of dramatic induction to *LHCB1*. In contrast, all other *TOC* genes were only moderately upregulated. These results suggested that *TOC75* expression plays a leading role during TOC biogenesis.

To investigate whether *TOC75* gene expression changes can affect the accumulation of TOC components, we first studied an inducible RNA interference (RNAi) line in which *TOC75* mRNA was reduced to ~ 20% of the wild-type level^23^ (Fig. 7b, c). Immunoblotting analysis showed that the reduced accumulation of Toc75 in the *RNAi-toc75* plants resulted in an overall reduction in the accumulation of all TOC-P and TOC-N components, while leaving TIC components unaffected. These results supported the view that *TOC75* expression can, to some extent, drive the accumulation of the TOC-P and TOC-N components. To further test this hypothesis, we employed two *HA-TOC75* expression lines (*HA-Toc75*, #2 and #3) that were generated in the *kd-toc75* background which, as described earlier, selectively reduces TOC (but not TIC) protein accumulation (Fig. 2a). In these lines, the expression of *TOC75* was enhanced 5–8 fold relative to wild type, while the expression of *TOC33* and *TOC159* was unaltered (Supplementary Fig. 15). Notably, immunoblotting revealed that all TOC-P and TOC-N proteins were recovered to at least wild-type levels in these overexpression *HA-Toc75* lines (Fig. 7d, e). In fact, Toc33 protein even exceeded the wild-type level despite *TOC33* transcript abundance being unchanged. Thus, on the basis of all these results, we concluded that Toc75 is a leading and central player in the biogenesis of TOC complexes.

### Toc75 synthesis precedes that of Toc159 in the TOC assembly process

**Fig. 8:**
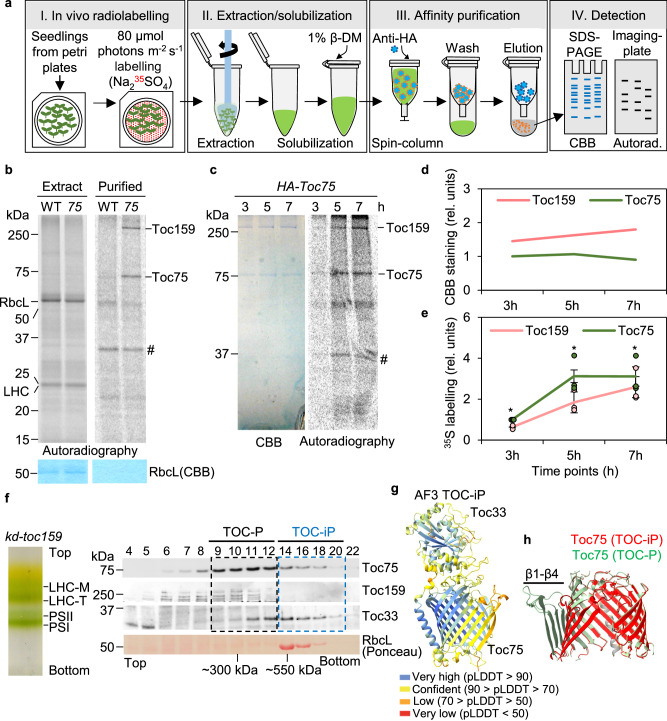
In vivo labelling analysis reveals the order of synthesis of TOC components. **a** Schematic summary of the experimental procedure. **b** Radiolabelling of ~ 7-day-old WT and *HA-Toc75* (*75*) transgenic seedlings with inorganic sulphate (Na_2_^35^SO_4_) for 16 h. Labelled protein samples extracted from the plants were subjected to HA-affinity purification and/or analysis by SDS-PAGE and autoradiography. Samples were solubilised with 1% β-DM before affinity purification. Total protein extracts (Extract) and affinity-purified samples derived from the total protein extracts (Purified) are shown. Positions of molecular weight markers (sizes in kDa), and two prominent chloroplast proteins (Rubisco large subunit, RbcL; Light-harvesting chlorophyll protein, LHC), are indicated at left. Resolved polypeptides were visualised by autoradiography. Samples were normalised according to fresh weight, and this was verified by detecting RbcL using Coomassie brilliant blue (CBB) staining. A non-specific band is indicated (#). **c** Similar radiolabelling of HA-Toc75 plants for shorter time periods. Affinity-purified samples from 3, 5 and 7 h labelling time points were prepared as in (**b**). Resolved polypeptides in the purified samples were visualised by either staining (CBB) or autoradiography. A non-specific band is indicated (#). **d**,** e** The CBB (**d**) and autoradiography (**e**) signals from (**c**) were quantified by densitometry. Asterisks indicate significance according to paired one-tailed Student’s *t* tests comparing the labelling of Toc159 with the labelling of Toc75 (*p < 0.015). All values in (**e**) are means ± SEM (*n* = 2-3 experiments). In (**e**) the labelling of Toc159 is divided by a factor of 1.25 (because the cysteine + methionine residue counts for Toc75 and Toc159 are 20 and 26, respectively). **f** Sucrose density gradient ultracentrifugation analysis of protein complexes in a chloroplast-enriched leaf fraction of the *kd-toc159* mutant. The gradient showed clear separation of pigment-protein complexes (left). Fractions from the gradient were analysed by immunoblotting and Ponceau staining (to detect RbcL) (right), enabling resolution of TOC-P (black) and TOC-iP (blue). **g**, **h** AF3 analysis of the TOC-iP complex. Polypeptide chains are coloured according to pLDDT scores (**g**) or protein identity (**h**); overlay of Toc75 from AF3 TOC-P (green) and AF3 TOC-iP (red) in (**h**) reveals open and semi-closed states, respectively.

To shed further light on the synthesis and assembly of TOC complexes, wild-type and *HA-Toc75* seedlings were subjected to in vivo radiolabelling using inorganic sulphate (Na_2_^35^SO_4_) over a time-course^46^. Very young seedlings were employed so as to capture an active phase of photosynthetic establishment. Total radiolabelled seedling extracts were solubilised and subjected to anti-HA affinity purification, and the purified complexes were analysed by SDS-PAGE and autoradiography (Fig. 8a).

After an extended labelling reaction (16 h), the labelling intensities of two chloroplast diagnostic proteins, Rubisco large subunit (RbcL) and light-harvesting complex protein (LHC), were identical in the two genotypes, indicating efficient labelling of the chloroplasts (Fig. 8b). However, the TOC complex could only be purified from the *HA-Toc75* line, as expected. The purified complex showed strong labelling of Toc75 and Toc159 (Toc33, which has a low cysteine/methionine content (9 residues), was unfortunately obscured by a comigrating nonspecific band), indicating that these components were undergoing active synthesis. Although the other TOC components (i.e., TOC-N components) were shown to be present in comparable samples by immunoblotting (Fig. 1f), their labelling was not detected.

Next, to reveal the precise order of synthesis, we analysed TOC complexes from shorter labelling reactions (3 to 7 h) (Fig. 8c). The samples were derived from young seedling (~7 days old), and the analysis was performed at a small scale (~40 seedlings) due to the need to conduct radiolabelling. Although purification yields were low, the staining (by Coomassie brilliant blue, CBB) of Toc159 and Toc75 was clearly detectable. The purified sample contained similar yields of Toc75 and Toc159 (staining was slightly higher for Toc159, likely because of its larger size) (Fig. 8c, d). However, the Toc75 protein was more rapidly labelled. Labelling of Toc75 was readily detectable from 3 h, and essentially plateaued by 5 h of labelling (Fig. 8c, e). The labelling pattern of Toc159 was similar, but progressed more slowly and was still rising at 7 h, never quite reaching the levels seen in Toc75 (after adjusting for the cysteine/methionine contents of the proteins: 26 in Toc159, 20 in Toc75) (Fig. 8e). These findings reveal that Toc75 is synthesised just before Toc159, likely as part of a step-wise assembly process. Unfortunately, it was not possible to follow Toc33 labelling in this experiment (as noted earlier).

### Identification of a TOC intermediate complex, TOC-iP

To determine if Toc33 can assemble with Toc75 in the absence of Toc159, we separated protein complexes from leaf extracts of the *kd-toc159* mutant, which accumulates only ~ 20% of the wild-type Toc159 amount, by sucrose density gradient ultracentrifugation, analysing the resolved fractions by immunoblotting (Fig. 8f). Observed co-fractionation of TOC-P components at the expected size range (fractions 9–12; around 300 kDa) suggested that the TOC-P complex is able to form in this mutant. However, co-fractionation of just Toc75 and Toc33 in heavier fractions (fractions 14-18; > 550 kDa) was also observed, which we interpret to be indicative of an intermediate complex (TOC-iP) that is stabilised by auxiliary factors during the assembly process (hence its larger size) (Fig. 8f). Supporting this view, quantitative mass-spectrometry analysis of the HA-Toc75 purifications identified OEP80 (Toc75-V), albeit at low levels (Supplementary Fig. 14d); OEP80 belongs to the Omp85 superfamily of proteins which assemble β-barrel proteins into bacterial and organellar outer membranes, and is implicated in TOC biogenesis^47,48^. Moreover, assembly of the TOC-iP complex was predicted by AF3 with good confidence (ipTM = 0.71) (Fig. 8g). Interestingly, the AF3 model showed that the first four β-sheets of Toc75 fold inwards, forming a semi-closed state in the absence of Toc159, rather than outwards to yield an open state, as in the mature TOC-P complex (Fig. 8h). This suggests that Toc159 completes the formation of the mature complex at the last step of assembly. These observations align with previous data^16,49,50^ obtained through analysis of in vitro translation products with reconstituted proteoliposomes or isolated chloroplasts showing that the insertion of Toc159 requires the actions of Toc75 and Toc33.

**Fig. 9:**
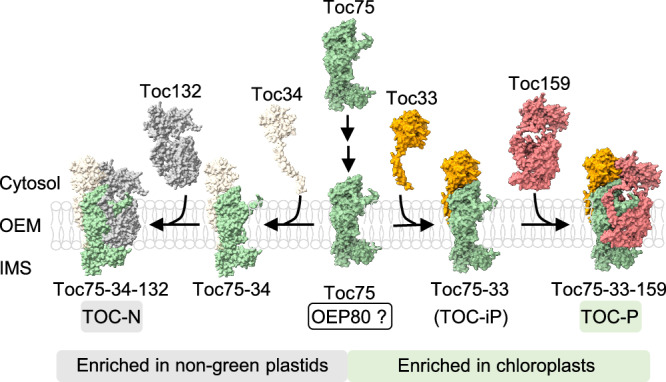
Model for the step-wise assembly of TOC complexes. Assembly is initiated by gene expression of *TOC75*, and upon translation, Toc75 is integrated into the outer envelope membrane of the organelle. Newly inserted Toc75 is stabilised by the integration of Toc33, forming a mutually-stabilised Toc75-Toc33 assembly intermediated complex (TOC-iP). In the next step, in chloroplasts, Toc75 rapidly assembles with Toc159 to yield the mature TOC-P translocon complex. Based on our genetic, biochemical and structural modelling data, we infer that Toc75 assembles sequentially with Toc34 and Toc132 (or the redundant Toc120) in a similar fashion to form TOC-N complexes; this is a minor process in chloroplasts where TOC-P predominates, but is the dominant process in non-green plastids such as leucoplasts where TOC-N predominates. The OEP80 protein may act as an assembly factor in the integration of the TOC complex.

Thus, we hypothesise that Toc75 is initially synthesised and inserted into the membrane, where it is stabilised through assembly with Toc33, forming a stable intermediate complex (TOC-iP) that subsequently and rapidly associates with Toc159 to form the mature TOC-P complex (Figs. 8b–f, 9). Although the TOC-N components were undetectable by either staining or radiolabelling in these experiments (reflecting the fact that TOC-P is the dominant configuration in photosynthetic tissues), based on our preceding analyses (e.g., Figs. 1, 2, 7 and Supplementary Fig. 14), we further infer that TOC-N biogenesis follows a similar pathway (Fig. 9).

### Receptor mutant analyses reveal functional differences between TOC-P and TOC-N

Having elucidated compositional and structural differences between the TOC-P and TOC-N complexes, we next wished to assess for functional differences. To this end, we employed a microscopy-based ratiometric approach to assess protein import in the *ko-toc33* and *ko-toc34* mutants, which lack characteristic receptors of TOC-P and TOC-N, respectively. For this, a protoplast-based import assay system was employed^51^, enabling assessment of multiple genotype and preprotein client combinations. Although the use of protoplasts does remove cells from their tissue-level context, we note that the system has been successfully applied to address numerous physiological questions^52,53^. To create model clients for these assays, the transit peptides (TP) of Rubisco small subunit precursor (SSU; a photosynthetic preprotein) and pyruvate dehydrogenase E1α subunit precursor (E1α; a non-photosynthetic preprotein)^15,54^ were fused with either YFP or CFP, yielding four fusions as follows: TP-SSU-YFP, TP-E1α-YFP, TP-SSU-CFP and TP-E1α-CFP.

**Fig. 10:**
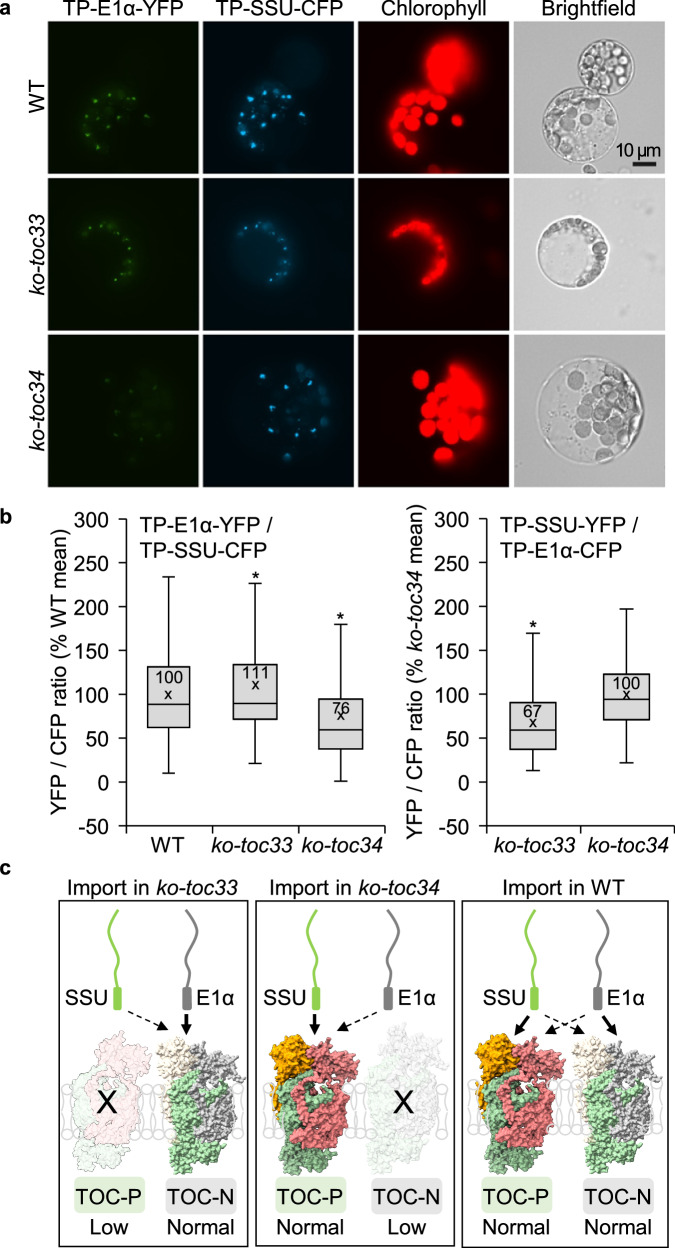
In vivo protein import analyses reveal client specificity differences between *ko-toc33* and *ko-toc34* mutants. **a**,** b** Ratiometric analysis of pairs of fusion proteins, each comprising a transit peptide (TP), either of Rubisco small subunit (SSU) or of pyruvate dehydrogenase E1α subunit (E1α), linked to YFP or CFP. Protoplasts from WT, *ko-toc33* or *ko-toc34* plants were co-transfected with plasmids encoding the indicated TP fusions, and fluorescence microscopy was used to detect the transiently-expressed proteins. Qualitative analysis of the relative chloroplast-targeting efficiencies of the TP-E1α-YFP and TP-SSU-CFP fusions is shown (**b**). The YFP (green; left panels) and CFP (cyan; centre-left panels) fluorescence signals were recorded, alongside chlorophyll autofluorescence (red; centre-right panels) and brightfield images (right panels). Each set of images is of the same cell and is representative of a large number of images derived from four independent experiments. The YFP/CFP fluorescence intensity ratios within individual chloroplasts were quantified (**b**), both for the experiment shown in (**a**) (left graph) and for the inverse experiment (right graph). For each genotype, the data shown comprise ~ 1000–2000 individual ratio measurements, derived from a total of up to 66 different protoplasts from four separate experiments. Asterisks indicate significance according to two-tailed Student’s *t* tests comparing the mutants with WT (*p < 0.001). In the box-whisker plots, the box, centre line, and cross-mark indicate interquartile range, median, and mean, respectively; outlier points are not shown (*n* = 904–1865). **c** Schematic representation of the protein import properties of the *ko-toc33* and *ko-toc34* mutants, and of WT. Import of TP-SSU preprotein (a photosynthetic client) is reduced in *ko-toc33*, whereas import of TP-E1α preprotein (a non-photosynthetic client) is reduced in *ko-toc34*. Thus, the SSU and E1α preproteins are preferentially imported by TOC-P and TOC-N, respectively, in the WT. Calculated pI values of the TPs of SSU and E1α are substantially different (10.05 and 12.13, respectively); this suggests that charge differences between TPs, together with surface electrostatic potential differences between TOC-P and TOC-N (see Supplementary Fig. 7f), may underly the observed client preferences.

First, these fusions were assessed in wild-type protoplasts to verify that their behaviour was as expected. After their expression in different pairwise combinations, the transfected cells were analysed by fluorescence microscopy. Although CFP fluorescence is inherently weaker than that of YFP, the TP-SSU-CFP fusion generally produced stronger chloroplast-associated fluorescence than TP-E1α-YFP in co-transfected cells, resulting in low YFP/CFP ratios (Supplementary Fig. 16a, b). In contrast, in reciprocal experiments using TP-SSU-YFP and TP-E1α-CFP, an opposite YFP/CFP ratio trend was seen (Supplementary Fig. 16b). Importantly, the observed fluorescence intensity differences were not due to differences in expression, as revealed by RT-PCR analysis (Supplementary Fig. 16c). These data indicated that the SSU TP was more effective at delivering protein import into photosynthetic chloroplasts, which is as expected. Therefore, this ratiometric system was implemented to analyse protoplasts from *ko-toc33* and *ko-toc34* mutants, to test for specific effects of the mutations on the import competence of the SSU and E1α TPs. In assays with TP-E1α-YFP and TP-SSU-CFP, higher YFP/CFP ratio values were seen in *ko-toc33* than in *ko-toc34* (Fig. 10a, b [left side of b]); whereas in the reciprocal experiment using TP-SSU-YFP and TP-E1α-CFP, the opposite trend was observed (Fig. 10b, right side).

Overall, these data provide in vivo support for the hypothesis that TOC-P and TOC-N are functionally distinct, in accordance with their respective predominance in photosynthetic and non-photosynthetic tissues, and owing to their possession of receptors with specificity for different transit peptides (Fig. 10c).

## Discussion

The protein translocon in the outer membrane of chloroplasts and other plastids, TOC, comprises an Omp85-type β-barrel channel, Toc75, and two related GTPase receptors, Toc33 and Toc159^1,3,4,6^. Because the receptors are encoded by small gene families in plants, it has long been proposed that different TOC systems may exist under different circumstances^3^. However, while evidence for functional differences among the various receptor isoforms has been presented, whether or not these isoforms indeed assemble selectively into distinct TOC complexes, for example, in different plastid types^11,13,14^ has remained unclear^17,55^. A major challenge in this area has been the low abundance of the TOC machinery. We circumvented such problems by employing transgenic plants expressing tagged TOC components to enable efficient affinity-purification strategies even from non-photosynthetic tissues. This formed just one strand of a heavily integrated approach (combining diverse experimental approaches with advanced structural analysis) that enabled us to detail the assembly of two structurally- and functionally-distinct TOC complexes, termed TOC-P and TOC-N, that predominate in photosynthetic and non-photosynthetic tissues, respectively.

After applying biochemistry and genetics to elucidate subunit interactions within the TOC-P and TOC-N complexes, we implemented an integrative structural approach (comprising AF3, negative staining EM, XL-MS, AlphaLink2, and MD) to uncover architectural features of the complexes and the differences between them. This analysis revealed a novel heterodimeric-GTPase–heterodimeric-β-barrel arrangement, with the two heterodimeric modules being coupled by a pair of linkers (Figs. 3, 4). In TOC-P, the GTPase module comprises Toc33 and Toc159, whereas the β-barrel module comprises Toc75 and Toc159. The latter module is well-aligned with recently-published cryo-EM structures of TOC-TIC supercomplexes from the green alga *C. reinhardtii*^30,31^. However, the published cryo-EM structures completely lacked information on the receptor GTPase domains, presumably due to the way they are flexibly linked to the β-barrel module, as our results reveal. This highlights a key advantage of the integrative approach employed here over solely cryo-EM-based approaches. We speculate that flexibility is a key feature of the TOC machine, enabling the receptor GTPase module to flip-flop back and forth so as to collect precursor protein clients in the cytosol and then deliver them into the β-barrel channel module. The TOC GTPase heterodimer calls to mind another interaction between related GTPases; that between SRP54 and its membrane receptor FtsY, which occurs during nascent protein delivery to the bacterial cell membrane^56^. However, in that case, the association is only transient in nature, whereas the TOC GTPases appear stably associated.

Even though the TOC β-barrel module is similarly arranged in plants and algae, our results show that the two systems have nonetheless diverged functionally. Whereas in algae the TOC is deeply and stably integrated with the TIC machinery^30,31^, we find that in plants the two translocons are readily separable, a result that aligns well with recent cryo-EM structures of the plant TIC and an associated Ycf2-FtsHi motor complex, which lack TOC components^57^. This feature may be a special adaptation in plants to enable greater flexibility in the import system. One can easily envision how it may be crucial to have much more dynamic TOC-TIC associations in plants, to enable full exploitation of the advantages that having multiple TOC subtypes can bring, for example, when protein import requirements change dramatically during development. This dynamism might be especially important during developmental phases in which the plastids change type via organellar proteome remodelling (such as in de-etiolation or fruit ripening), and it may be facilitated by the proteolytic removal of unneeded TOC components^1,21,58^. Central to this hypothesis is a need for variant TOCs that differ functionally, and our data clearly indicate that this requirement is met. The TOC-P and TOC-N complexes have structural differences, including surface electrostatic potential differences at the entry side of the complex, as well as functional differences in relation to preprotein client specificity (Fig. 10 and Supplementary Fig. 7).

On consideration of our in vivo labelling results in conjunction with the genetics, expression and structural data, we proposed a model for TOC biogenesis (Fig. 9). In this, new TOC complexes are formed in a co-assembly process in which Toc75 plays a driving role. Synthesis of Toc75 primes a new complex, which is then stabilised in the membrane by the addition of Toc33 before final completion with the insertion of Toc159 (Fig. 9). This model is consistent with previous in vitro reconstitution experiments conducted using isolated chloroplasts or proteoliposomes^16,49,50^, and hypotheses founded on the data^17^. Those studies showed that resident Toc33 and Toc75 are required to integrate Toc159 into the membrane, although the order in which Toc33 and Toc75 themselves are assembled was not addressed. Here, we employed native protein biochemistry and genetic analyses to provide a more comprehensive and physiologically-relevant picture of TOC assembly, delivering a deeper and robust understanding of the assembly process (Figs. 2, 7, 8). It was further suggested that a heterodimeric interaction of the Toc159 GTPase domain with that of Toc33 promotes the integration of the Toc159 membrane domain^50,59^, revealing an additional parallel with our results. Other studies showed that resident Toc75 is sufficient to mediate the insertion of outer membrane proteins with similar characteristics to the Toc33 component^60^, which is again consistent with our TOC biogenesis model (Fig. 9).

It is also informative to consider TOC biogenesis from an evolutionary perspective. As a member of the Omp85 superfamily of proteins^61^, Toc75 is clearly of prokaryotic origin and most likely derived from a cyanobacterial Omp85-type protein during endosymbiosis. Thus, we may infer that it was the first component to be recruited to the nascent protein import system of the chloroplast outer envelope membrane during the evolution of photosynthetic eukaryotes^62^. The presence of eukaryotic-type GTPase domains in the receptors, Toc33 and Toc159, led to the further conclusion that these components are of eukaryotic origin and were therefore added later^63^. The large receptor, Toc159, further evolved with the addition of terminal extensions at both ends (the N-terminal acidic domain and the C-terminal membrane domain), delivering new capabilities related to client selectivity and channel flexibility. Interestingly, the Toc159 membrane β-barrel domain shares considerable similarities with the mitochondrial protein import channel, Tom40 (as well as with the related metabolite channel, VDAC)^64–66^, suggesting a remarkable evolutionary link between the protein import systems of the two prevalent endosymbiotically-derived organelles in eukaryotic cells (Supplementary Fig. 17). Regardless, it is clearly apparent that our TOC assembly model (in which Toc75 plays a leading role) is well aligned with these evolutionary aspects.

In conclusion, we have defined the properties of two distinct TOC complexes in plant plastids. The TOC-P and TOC-N complexes are structurally and functionally distinct, and are differentially synthesised and assembled to meet differing protein import demands in different plastid types. Furthermore, we have shown that TOC biogenesis is a dynamic and hierarchical process, in which the channel component Toc75 plays a central and driving role. Future work should seek to identify auxiliary factors that support the integration of new components during TOC complex assembly, in order to further elucidate the biogenesis mechanism. Moreover, our results suggest that fine-tuning the expression of Toc75 has the potential to enhance TOC accumulation, protein import, and possibly even photosynthesis, suggesting novel strategies for crop improvement.

## Methods

### Plant materials and growth conditions

All plants used were *Arabidopsis thaliana* (Columbia-0). For most experiments, plants were grown for 7–14 days on Murashige and Skoog (MS) medium under long-day photoperiods (16 h light / 8-h dark; 150 µmol m^–2^ s^–1^) and at 22 °C. For propagation, plants were grown on soil under similar conditions in a greenhouse. The following, previously-described TOC mutant genotypes were employed in this study: *ko-toc33* (*ppi1-1*)^14^, *ko-toc34* (*ppi3-2*)^12^, *kd-toc159* (*fts1*, *ppi2-3*)^21,25^, *kd-toc75* (*mar1*, *toc75-III-3*)^23,24^, *RNAi-toc75* (atToc75-III ↓ #6)^23^, and *ko-toc132* (*toc132-2*)^15^. The generation of the *HA-Toc75* lines is described below.

For de-etiolation experiments, seeds were germinated and grown in the dark at 22 °C for 7 days as previously described^19^. Then, the dark-grown seedlings were exposed to light-dark cycles (16 h light / 8 h dark) for 7 days. Samples were collected at different time points to conduct gene expression analysis.

### Generation of *HA-Toc75* transgenic lines

Genomic DNA corresponding to the entire *TOC75* gene (At3g46740), including the native promotor and untranslated regions, was amplified by PCR from wild-type leaves using primers (gToc75-F/gToc75-R) designed to anneal 1005 bp upstream of the *TOC75* start codon and 504 bp downstream of the *TOC75* stop codon, and then cloned into the pGEM-T Easy vector (Promega) (Supplementary Data 4a). A nucleotide sequence (5’-TATCCTTATGATGTTCCTGATTATGCT-3’) encoding a haemagglutinin (HA)-peptide was inserted between codons 2 and 3 of the mature Toc75 protein (E142-HA-E143 in the preprotein) by inverse PCR using a set of primers: mToc75-HA-F and mToc75-HA-R (Supplementary Data 4a). Then, the full-length *HA-TOC75* sequence was cloned into the plant binary vector pPZP221, generating a transformation vector, pPZP221-HA-Toc75^14,67^. Following transformation into agrobacterium, the plants were transformed by the floral dip method as reported earlier^14,68^. *HA-Toc75* transformants were selected on MS medium containing 110 μg mL^−1^ gentamycin, and homozygous T3 plants were obtained after segregation analysis. The *HA-Toc75* transgenic lines exhibited different levels of *TOC75* expression. The second and third lines exhibited unusually high levels of* TOC75* expression (Supplementary Fig. 15) and were therefore employed as overexpressor lines (*HA-Toc75*, #2 and #3).

### Affinity purification of HA-Toc75, TAP-Toc33 and TAP-Toc159

To purify HA-Toc75/TAP-Toc33/TAP-Toc159 complexes, a crude chloroplast preparation was made as described^69^. Seedlings were homogenised in chloroplast isolation (CI) buffer (0.3 M sorbitol, 5 mM MgCl_2_, 5 mM ethylene glycol tetraacetic acid (EGTA), 5 mM ethylenediaminetetraacetic acid (EDTA), 10 mM NaHCO_3_, 20 mM 4-(2-hydroxyethyl)piperazine-1-ethanesulfonic acid (HEPES)-KOH, pH 8.0) and centrifuged at 1000 × *g* for 5 min. The pellet was resuspended in HEPES-MgSO_4_-sorbitol (HMS) buffer (50 mM HEPES-NaOH, pH 8.0, 3 mM MgSO_4_, 0.3 M sorbitol)^69^. Alternatively, whole cell extracts were prepared from roots or etiolated seedlings using the CI and HMS buffers as described above^69^.

Then, the samples were solubilised with 1% (w/v) β-dodecyl maltoside (β-DM) for 10 min and centrifuged at 20,000 × *g* for 10 min^46^. Supernatant (500 µL) was loaded onto a small 1 mL spin-column containing 20–50 µL of anti-HA beads (HA-tagged protein purification kit [code: 3320], MBL) for HA-Toc-75 or IgG-sepharose beads (IgG-sepharose 6 fast flow [code: 17-0969-01], Cytiva) for TAP-Toc33/TAP-Toc159, and the samples were mixed end-over-end for 1 h. Flow-through was removed by centrifugation for 10 s in a microcentrifuge. Then, the column was washed for 3-5 times with wash buffer (MBL). HA-Toc75 samples were eluted with an elution buffer containing HA peptide (MBL). TAP-Toc33/TAP-Toc159 samples were eluted after digesting with TEV protease (7.5 units/μL) for 2 h at 16 °C^21^. After elution, the samples were concentrated with 30 kDa cut-off ultrafilters (Sartorius). All the extraction and purification steps were performed at 4 °C unless specified otherwise.

### Protein extraction, SDS-PAGE and immunoblotting

Protein samples were prepared from different plant tissues by homogenisation in protein extraction buffer (50 mM Tris-HCl, pH 7.6, 1 mM phenylmethylsulfonyl fluoride (PMSF), 10% (v/v) glycerol, 0.1% (w/v) β-DM, 1 × protease inhibitor cocktail (Sigma)). Polyacrylamide gels (10–15%) were cast using high-Tris gel systems as reported previously^70^. Proteins were heat-denatured at 95 °C in the presence of 100 mM dithiothreitol (DTT) and 2% (w/v) sodium dodecyl sulphate (SDS), for either 3 min (for purified samples) or 5 min (for whole cell extracts). After electrophoresis, proteins were visualised by staining with either Flamingo fluorescent dye (Bio-Rad), silver nitrate, or Coomassie brilliant blue. Alternatively, proteins were transferred onto a nitrocellulose membrane to enable probing with different antibodies. The primary antibodies used in this study were as follows: anti-atToc75-III POTRA-domain (1:1000)^21,71^; anti-atToc159 A-domain (1:5000)^21,71^; anti-atToc33 peptide (1:500)^21,71^; atToc34 (Agrisera, AS07 238) (1:2000); anti-atToc132 A-domain (1:1000)^21,71^; anti-atToc120 A-domain (1:1000)^71^; anti-atTic110 stromal domain (1:5000)^71^; anti-atTic40 stromal domain (1:100000)^71^; anti-atTic56 (1:5000)^39^, anti-atTic100 (1:5000)^39^, anti-atTic214 (1:5000)^39^, anti-actin (1:3000; AS132640, Agrisera); anti-histone H3 (1:1000; ab1791, Abcam); and anti-HA (1:1000; H6908, Sigma). The secondary antibody was anti-rabbit IgG conjugated with horseradish peroxidase (Sigma, 12-348) (1:5000). Multiple bands (100–250 kDa) detected with antibodies against large TOC receptor isoforms (Toc159, Toc132, Toc120) are likely caused by fragmentation during extraction^22^. Immunodetection was by chemiluminescence and monitored using an ImageQuant LAS-4000 imager (GE Healthcare). The protein bands (staining/radiolabelling) were quantified using ImageJ^72^ and the raw data were exported to Microsoft Excel and analysed (Figs. 2a, b, 7b–e, 8c–e). All relevant raw data are presented in the Source Data file.

Membrane fractions from wild-type and *kd-toc159* mutant leaf extracts were prepared as described. These fractions were washed with 100 mM Na_2_CO_3_ for 30 min. Samples were centrifuged (22,000 × *g*) for 30 min, and the pelleted and soluble fractions were analysed by SDS-PAGE and immunoblotting as described above.

Crude chloroplasts from wild-type and *kd-toc159* plants were solubilised in 1% β-DM as described above with the addition of 1 mM DTT, and the solubilised protein complexes were separated by sucrose density gradient ultra-centrifugation^73^. Four distinct green bands, LHC-M and LHC-T (LHCII), photosystem II (PSII core), and photosystem I (PSI-LHCI), were identified after separation. The resulting fractions were analysed by SDS-PAGE and immunoblotting.

### Gene expression analysis by qRT-PCR

RNA extractions were performed using a Spectrum Plant Total RNA kit (Merck). Reverse transcription was performed by using SuperScript IV reverse transcriptase (Invitrogen). For quantitative reverse transcriptase PCR (qRT-PCR), a qPCRBIO SyGreen Mix Hi-ROX kit (PCR Biosystems Ltd.) was employed, together with a StepOnePlus Real-Time PCR System (Applied Biosystems) and its associated software according to the manufacturer’s instructions. The primers used for PCR amplification are shown (Supplementary Data 4b). Expression data for genes of interest were normalised using data for *ACTIN2* (At3g18780).

### Radiolabelling

For each genotype, forty ~ 7-day-old seedlings were collected into 3 mL MS liquid medium in a 6-well microtitre plate. Seedlings were incubated with 30 µCi/mL of Na_2_^35^SO_4_ for 3–16 h at 22 °C under 80–100 µmol photons m^−2^ s^−1^ white light^46,74^. Radiolabelling was stopped by adding 100 mM cold Na_2_SO_4_. Seedlings were collected into a 1.5 mL microfuge tube and homogenised using a small plastic pestle. Protein extraction was performed as described earlier, and the seedling extract was further subjected to anti-HA affinity purification, also as described earlier. After affinity purification, the samples were resolved by SDS-PAGE and then imaged by staining or phosphorimaging (Storm Molecular Imager, Molecular Dynamics).

### Protoplast isolation, transfection and analysis

The transit peptide fusions of the Rubisco small subunit (SSU; At1g67090) and pyruvate dehydrogenase E1α subunit (E1α; At1g01090) to YFP and CFP were generated using the p2GWY7 and p2GWC7 vectors^75^ by Gateway cloning (Supplementary Data 4c). In each case, at least seven residues of the mature part of the preprotein were retained, so as not to disrupt the stromal processing peptidase cleavage site or targeting efficiency. The transit peptide coding sequences were amplified from first-strand cDNA or plasmid cDNA clones using gene-specific primers (Supplementary Data 4c). pI values of the TPs of SSU and E1α in Fig. 10 were calculated using the ExPASy online server (https://web.expasy.org/compute_pi/)^76^.

Protoplasts were prepared from 2-4-week-old plants and transfected using well established methodologies^77,78^. Transfections employed 5 µg plasmid DNA (per construct) and 100 µL protoplast suspension (2 × 10^5^ cells). The transformed protoplasts were analysed by either RT-PCR or fluorescence microscopy.

For RT-PCR analysis of transient expression, total RNA was isolated from transfected (or untransformed) protoplasts using an RNeasy Plant Mini kit according to the manufacturer’s instructions (Qiagen). DNAse І treatment and first-strand cDNA synthesis (using 3 μg RNA) were conducted as described previously^79^. To assess expression of the transit peptide fusions, RT-PCR employed primers specific for each construct as follows: SSU-F, E1α-F, and CFP/YFP-R (Supplementary Data 4d). In each case, the *eIF4E1* (At4g18040) gene was employed as a housekeeping control for normalisation purposes, using the following primers: eIF4E1-F/eIF4E1-R. Following electrophoresis, bands were visualised by staining with SYBR Safe (Invitrogen) and quantified using Aida software (Raytest).

For fluorescence microscopy, samples were analysed 16–24 h after co-transfection using a Nikon Eclipse TE-2000E inverted fluorescence microscope equipped with filters for analysing YFP (exciter HQ500/20x, emitter HQ535/30 m), CFP (exciter D436/20x, emitter D480/40 m), and chlorophyll autofluorescence (exciter D480/30x, emitter D660/50 m) (Chroma Technologies). For each plasmid combination, image sets of co-expressing protoplasts were captured in at least three independent transfection experiments. The ratio of exposure times for YFP and CFP was kept at 1:1 throughout all experiments and for all genotypes. Exposure times ranged from 100 ms to 500 ms, depending on the brightness of the signal, and the gain setting was zero at all times; the YFP images were always captured first. Quantification of the YFP:CFP signal intensity ratios was conducted using Openlab software (Improvision). Firstly, ratio images of corresponding YFP/CFP image pairs were generated using the “Ratio” software module; all ratios were measured as YFP over CFP. To avoid unspecific skewing of the ratio data for individual chloroplasts, the “Density Slice” module was employed, which allows the specific selection of signal spots within chloroplasts (this was necessary because we observed that the fluorescence within chloroplasts was not uniformly distributed). A density slice layer was generated for each image pair and then applied for the extraction of the ratio data from individual signal spots. Cells expressing only one of the two fluorophores were rare ( ~ 10%) and were not analysed.

### Transient expression in leaves and co-immunoprecipitation analysis

The GTPase domains of Toc33 (amino acids 1-265) and Toc159 (amino acids 830–1078) were expressed from pH2GW7-FLAG and pH2GW7, respectively^71^. The nucleotide sequence encoding the Toc33 GTPase domain (Toc33G) was amplified by PCR using primers shown in Supplementary Data 4e. The nucleotide sequence encoding the Toc159 GTPase domain (Toc159G) with a C-terminal HA-tag and attB recombination sites was synthesised by Integrated DNA Technologies B.V. (IDT). A GFP construct in the pH2GW7-FLAG vector was used as a negative control. Expression of these constructs was driven by the cauliflower mosaic virus (CaMV) 35S promoter. The constructs were individually transformed into *Agrobacterium tumefaciens* (GV3101), and the transformants were infiltrated into leaves of *Nicotiana benthamiana* plants in the indicated combinations.

For co-IP assays, 100 mg of fresh agroinfiltrated leaves^80^ were homogenised using metal beads using a Qiagen TissueLyser II with IP lysis buffer (25 mM Tris-HCl, pH 7.6, 150 mM NaCl, 1% NP-40, 1% sodium deoxycholate) containing 1 mM PMSF and 1 × cOmplete Protease Inhibitor Mixture (Roche). Extracts were centrifuged in a microfuge at 17,800 × *g* for 10 min at 4 °C. Supernatants were incubated with 25 μL of Pierce Anti-FLAG magnetic agarose beads (Thermo Fisher) at 4 °C overnight. Finally, the agarose beads were washed using lysis buffer, and then the proteins were eluted with 100 µL of SDS-PAGE sample buffer at 100 °C for 10 min. Proteins were detected by immunoblotting.

### Negative staining and electron microscopy (EM)

A thin clean film of carbon on top of a copper mesh 200 (C101/100, TAAB) was glow-discharged for 25 s at 15 mA current (Pelco, easiGlow) before 3 μL of a freshly-purified TOC-P sample was applied directly onto the grid. After 1 min of incubation at ambient temperature, the sample was absorbed by touching the side of the grid with a wedge of filter paper (Whatman, 1001-090). The grid was rapidly dropped, face downwards, on top of a 20 μL drop of 2% uranyl acetate solution dispensed onto the surface of Parafilm, where it was then incubated for 10 s. The excess stain was blotted away until a thin film remained, and the grid was then air-dried for 5 min. A total of 195 images were collected at a nominal magnification of 50,000 and a pixel size of 2.3 Å on a 200 kV electron microscope (JEM-2100Plus; The Dunn School Electron Microscopy Facility, Oxford).

### EM data processing

The raw images were processed in cryoSPARC^81^, where CTFFIND4^82^ was used for Contrast Transfer Function (CTF) estimation using a higher amplitude contrast of 0.25 at a minimum resolution of 50 Å. Initially, particles were either picked manually or using the blob picker with a particle diameter in the range of 50-200 Å. The best classes were then selected for the template picker using 150 Å particle diameter. After extracting about 20,000 particles with a box size of 200 pixels, two rounds of 2D classifications were run with a constant CTF for output particles. The best 2D classes comprising about 8,000 particles were used for comparison with 2D projections of the AF3-generated model of the TOC-P complex.

Two-dimensional (2D) back-projections of the AlphaFold3 (AF3)-predicted TOC-P complex map were generated in cryoSPARC using the Create Templates function with default parameters (50 evenly spaced templates and a zero-padding factor of 2 for interpolation). The AF3-derived TOC-P map was generated in UCSF ChimeraX v1.9 using the molmap command at an arbitrary resolution of 5 Å. A model map with a uniform box size of 50 pixels was generated in RELION v 5.0.0 using the relion_image_handler program.

### Proteomics analysis

#### Sample preparation

In total, five different proteomics experiments are described, all of which involved analysis of HA-affinity purified TOC complexes. These five experiments were: (i) in-gel and (ii) in-solution proteomic analyses to determine complex composition (Supplementary Fig. 2); (iii) in-gel and (iv) in-solution crosslinking mass spectrometry analyses performed using two different concentrations of crosslinker to determine contact sites in the purified complex (Fig. 3f and Supplementary Fig. 6b, c); and, (v) label-free quantification analysis to identify TOC-interacting components (Supplementary Fig. 14d). For the crosslinking experiments (Supplementary Fig. 6b), the HA-purified complex was crosslinked with 0.5 mM bis(sulfosuccinimidyl)suberate (BS3) (Thermo Fisher Scientific) at room temperature for 30 min (experiment iii) or with 3 mM BS3 at room temperature for 3 h (experiment iv). Crosslinking reactions were terminated by adding 50 mM Tris, pH 7.5. The samples were processed for mass spectrometry either before or after separation of polypeptides by SDS-PAGE.

The two in-gel experiments (i and iii) were performed as follows. For experiment i, proteins shown in Supplementary Fig. 2 were excised, destained and digested as detailed below. For experiment iii, silver-stained high molecular weight SDS-PAGE smears following BS3 crosslinking were excised (Fig. 3f and Supplementary Fig. 6b, c), transferred into Protein LoBind Eppendorf tubes, and destained with 15 mM potassium ferricyanide and 50 mM sodium thiosulfate. Gel pieces were resuspended in 0.1 M ammonium bicarbonate, pH 8.0, and then incubated with 500 µL of 100% acetonitrile. Supernatants were discarded, and dried gel pieces were incubated with 50 µL of 10 mM tris(2-carboxyethyl)phosphine (TCEP) for 30 min at room temperature, and with 50 mM 2-chloroacetamide (2-CAA) for 30 min at room temperature in the dark. Supernatants were discarded, and gel pieces were incubated with 500 µL of 100% acetonitrile. Dried gel pieces were further resuspended in 50 µL of 10 ng/µL trypsin solution in 100 mM ammonium bicarbonate and 10% acetonitrile (digestion buffer), and incubated for 16 h at 37 °C with shaking at 650 rpm. After digestion, supernatants were collected, and peptides were extracted from the gel with 5% formic acid/acetonitrile (1:2, vol:vol) for 15 min at room temperature with shaking at 650 rpm. Recovered peptides were dried in a speed-vac and resuspended in 5% acetonitrile / 5% formic acid before LC-MS/MS analysis.

For the in-solution experiments (ii, iv and v) (Fig. 3f, Supplementary Figs. 2, 6b, 14d and Supplementary Data 1–3), the detergent (β-DM) was removed by using HiPPR detergent removal resin (Thermo Fisher Scientific) prior to the preparation of the samples for mass spectrometry. The samples were denatured in 4 M urea (in 100 mM ammonium bicarbonate, pH 7.8) for 10 min, before cysteine reduction with 10 mM TCEP for 30 min at room temperature and alkylation with 50 mM 2-CAA for 30 min at room temperature in the dark. Next, the samples were pre-digested with endoproteinase LysC (Wako) (1:100 µg enzyme:protein ratio) at 37 °C for 2 h with shaking, and then further digested with trypsin (Promega) (1:40 µg enzyme:protein ratio) for 16 h with shaking. The digests were centrifuged at 11,688 × *g* at 4 °C to remove aggregates, and the supernatants containing digested peptides were loaded onto C18 stage tips pre-activated with 100% acetonitrile and 0.1% trifluoroacetic acid. After centrifugation, peptides were washed with 0.1% trifluoroacetic acid and eluted in 50% acetonitrile / 0.1% trifluoroacetic acid. Eluted peptides were dried in a speed-vac. Dried peptides were resuspended in 5% acetonitrile / 5% formic acid before LC-MS/MS analysis.

#### LC-MS/MS analysis

For experiment i, samples were processed over a 15 min LC gradient; while for experiments ii, iii, iv and v, samples were processed over 60- and 120 min gradients to enhance proteome depth. Peptides were separated by nano liquid chromatography (Thermo Scientific Ultimate RSLC 3000) coupled in-line to a QExactive mass spectrometer equipped with an Easy-Spray source (Thermo Fisher Scientific). Peptides were trapped onto a C18 PepMac100 precolumn (300 µm i.d., 5 mm length, 100 Å; Thermo Fisher Scientific) using Solvent A (0.1% formic acid, HPLC grade water). The peptides were further separated onto an Easy-Spray RSLC C18 column (75 µm i.d., 50 cm length, 100 Å; Thermo Fisher Scientific) using 15, 60 and 120 min linear gradients (15% to 35% Solvent B (0.1% formic acid in acetonitrile)) at a flow rate 200 nL/min. The raw data were acquired on the mass spectrometer in a data-dependent acquisition (DDA) mode. Full-scan MS spectra were acquired in the Orbitrap (scan range 350–1500 m/z, resolution 70,000, AGC target 3e6, maximum injection time 50 ms). The 5 (15 min gradient), 10 (60 min gradient), and 20 (120 min gradient) most intense peaks were selected for higher-energy collision dissociation (HCD) fragmentation at 30% of normalised collision energy. HCD spectra were acquired in the Orbitrap at resolution 17,500, AGC target 5e4, maximum injection time 120 ms with fixed mass at 180 m/z. Charge exclusion was selected for unassigned and 1 + ions. The dynamic exclusion was set to 5, 20 and 40 s for 15, 60 and 120 min gradients, respectively.

#### Data analysis

For experiments i, ii and v (Supplementary Data 1 and 3), tandem mass spectra were searched using Sequest HT in Proteome Discoverer software version 1.4 against an *Arabidopsis thaliana* database downloaded from UniProt (Proteome ID UP000006548; released 14/03/2024) containing 18,300 protein entries. To this list, 292 contaminants were additionally added. In the database, the Toc75-III protein sequence was replaced with HA-tagged Toc75, named as USER_DEF_HA_Toc75. During database searching, we considered cysteines (C) to be fully carbamidomethylated ( + 57.0215, statically added), methionines (M) to be fully oxidised ( + 15.9949, dynamically added), all N-terminal residues to be acetylated ( + 42.0106, dynamically added), and asparagines (N) and glutamines (Q) to be fully deamidated ( + 0.984, dynamically added). Two missed cleavages were permitted. Peptide mass tolerance was set at 20 ppm on the precursor and 0.6 Da on the fragment ions. Data were filtered at FDR below 1% at the PSM level. Perseus version 2.0.11 was used for downstream bioinformatic analysis and label-free quantification analysis of HA-Toc75 versus wild-type negative control (*n* = 3). Median normalisation was performed on log2-transformed protein intensity values followed by filtering data to retain 2 over 3 valid values amongst the three replicate samples and present in at least one group. Imputation was performed by replacing missing values from the normal distribution. An unpaired Student’s *t* test was performed between conditions at S0 = 0.1 and FDR ≤ 0.05.

For experiments iii and iv (crosslinking mass spectrometry) (Supplementary Data 2), acquired tandem mass spectra were searched using pLink 3^83^ and MetaMorpheus 1.0.9^84^, against an *Arabidopsis thaliana* database downloaded from UniProt (Proteome ID UP000006548; released 14/03/2024) containing 18,300 protein entries and the HA-tagged Toc75 protein sequence (USER_DEF_HA_Toc75). In addition, 292 contaminant sequences were added to the database while processing with pLink 3. The default contaminant database of MetaMorpheus was used. The following variable modifications were set: Oxidation (M), Acetylation (N-ter). Carbamidomethylation was set as a fixed modification. Data were filtered at an FDR below 5%. Trypsin was set as the endoprotease. All identified crosslinked spectra were manually inspected and validated. The TOC crosslinked spectra (#1-21) are displayed in Supplementary Fig. 18.

### AlphaFold3 and AlphaLink2 analysis

AF3 predictions were performed in the on-line server (https://alphafoldserver.com)^26^. To generate TOC-P models, the component sequences of TOC-P were submitted in the following order: 1. the mature protein sequence of Toc75 (D141-Y818); 2. truncated Toc159 (E781-Y1503); 3. full-length Toc33 (M1-297L); and 4. two GTP ligands (Fig. 3a). To generate TOC-N models, the component sequences of TOC-N were submitted in the following order: 1. the mature protein sequence of Toc75 (E143-Y818); 2. truncated Toc132 (A486-Q1205); 3. full-length Toc34 (M1-S313); and 4. two GTP ligands ﻿(Supplementary Fig. 7). TOC complex protein sequences from the green alga, *C. reinhardtii*, were submitted in the following order: 1. the mature protein sequence of Toc75 (E128-F798); 2. truncated Toc90 (P190-L967); 3. full-length Toc34 (M1-D396); and 4. two GDP ligands. For each prediction, five models were generated and ranked in order of their scores. Based on their ranking scores and architecture, they were found to be similar overall. To generate models of TOC-iP (Toc75-Toc33), of the membrane module (membrane domains of Toc33-Toc75-Toc159), and of the receptor module (GTPase domains of Toc33-Toc159), relevant TOC sequences were submitted to AF3 prediction, as detailed in the Source Data file. The top-scoring models were thoroughly studied to reveal the structural features by Chimera-1.17 (https://www.cgl.ucsf.edu/chimera/). The models shown in this paper are all derived from top-scoring predictions. Confidence was based on predicted alignment error (PAE), predicted local distance difference test (pLDDT), predicted template modelling (pTM), and interface predicted template modelling (ipTM) scores. Surface electrostatic potential was calculated using Chimera-1.17 and interpreted qualitatively.

For TOC-P, the ipTM score was 0.72, which is below the threshold (0.8) typically considered to indicate high confidence. It is important to note that this ipTM score reflects the accuracy (confidence) of the entire TOC-P complex model, including its unstructured regions. As our MD simulations showed, TOC-P is flexible, particularly at the interface between the receptor and channel modules around the two disordered linkers (Figs. 3d, 5b and Supplementary Movies 1, 2). This interface lies at the membrane plane, which AF3 does not account for; thus, AF3 cannot reliably model the relative orientation of membrane-embedded and soluble regions. Because ipTM measures the accuracy of relative subdomain positioning, long disordered linkers disproportionately lower the score. Notably, when these issues are excluded by analysing subcomplexes separately, AF3 predicts the domain architectures of TOC-P with high confidence (ipTM > 0.8) (Supplementary Fig. 5).

We ran the predictions of the TOC complex structures using the AF3 server, either using PDB templates with a default cut-off date of 30/09/2021, or without using PDB templates at all. In both cases, our structure predictions converged to near-identical models of the TOC complexes with high confidence metrics as described. It is important to note that the algal cryo-EM TOC-TIC structures (accessions 7VCF and 7XZI)^30,31^, which were released after November 2022, were not included in the AF3 training.

AlphaLink2 was performed using AlphaLink2 Notebook^28,29,85^. The following arguments were provided: model weight, 2.2 (trained on 25 A CA-CA); msa mode, MMseq2; max_recycling iters, 3; crosslink_distance cutoff, 25. The TOC sequences were provided in FASTA format, and crosslink coordinates were provided in the following order: residueFrom chain1, residueTo chain2, FDR. The final model from AlphaLink2, termed AL-TOC-P, is shown in Fig. 3g and Supplementary Fig. 6d. It is important to note that AlphaLink2 implements AlphaFold-Multimer to predict the structures. Overall AL-TOC-P model confidence was 0.71; pTM, 0.72; ipTM, 0.70; crosslink satisfaction, 0.79; mean distance of crosslinked residues, 23.35 Å.

All the computational raw models derived from AF3 and AlphaLink2 are displayed in Supplementary Figs. 19 and 20, respectively.

### Phyre2 and I-TASSER analysis

The membrane domain of Toc159 (residues 1045-1503) was submitted to the Phyre2 protein threading server (http://www.sbg.bio.ic.ac.uk/phyre2/html/page.cgi?id=index)^65^ and to the I-TASSER protein structure and function prediction server (https://zhanggroup.org/I-TASSER/)^66^. The models generated by the Phyre2 server were analysed by Chimera as above (Supplementary Fig. 17). The models generated by Phyre2 were validated by parallel I-TASSER analysis which produced similar results.

### Molecular dynamics simulations

The AF3 TOC-P model shown in Fig. 3c, d was shortly minimised with position restraints of 10 kJ mol^−1^ Å^−2^, with the CHARMM36 forcefield converging after 118 steps. The resulting structure was used as a starting point for the coarse-grained (CG) modelling. The topologies to run CG simulations were generated with the martinize tool, choosing the Martini v.2.2 force field with an elastic network^86,87^. The force bond constant was set to 5 kJ mol^−1^ Å^−2^ with lower and upper elastic bond cut-offs of 5 and 9 Å, respectively. Elastic bonds were checked to make sure linker regions were kept flexible. This implementation of the coarse-grained forcefield we have used for describing the protein conformational dynamics constrains the secondary structure of proteins but allows flexibility in unstructured regions. We and others have previously shown that the dynamics afforded by this approach agrees well and can rationalise experimental data^88,89^. The protein was then embedded in a symmetric POPC bilayer using the *insane* tool and solvated in a 150 mM NaCl solution, resulting in a box of dimensions 200 × 200 × 200 Å^3^ (Fig. 5a). Minimisation was performed for 1000 steps with steepest-descent and position restraints of 10 kJ mol^−1^ Å^−2^, followed by a step-wise equilibration increasing the time-step from 10 fs to 20 fs and decreasing position restraints. During the equilibration process, the pressure was semi-isotropically coupled via a Berendsen barostat to a reference value of 1 bar with a coupling constant of 5 and compressibility of 3e^−4^ bar^−1^, while the temperature was coupled in the same way as the production run. Finally, a production run was performed at 323 K and 1 bar. Temperature was maintained via V-rescale thermostat with a coupling constant of tau_t = 1 ps, whereas semi-isotropic pressure coupling was achieved via a Parinello-Raham barostat with a coupling constant tau_p = 12 ps and compressibility 3e-4 bar^-1^. Three replicates of coarse-grained molecular dynamics simulations were run in GROMACS v2021.4^90^ for 10 µs each. To minimise structural bias from the initial starting point, initial particle velocities were assigned according to a Maxwell-Boltzmann distribution for each replicate independently.

Root mean square deviations were calculated in GROMACS using the gmx rms tool, considering only backbone beads. The RMSD values of the GTPase domains of interest were computed and plotted after fitting on the whole transmembrane region of the modelled TOC complex. For this analysis, the linker regions were defined to include only unstructured elements of the proteins spanning the following residues: linker-1 of Toc33, D251-S260; linker-2 of Toc159, L1077-R1086 (amino acid coordinates in the truncated Toc159 included in the AF3 structure are L297-R306). Lipid-protein contact analysis was computed over the whole 10 µs-long simulation (Supplementary Movies 1 and 2). A contact was defined when any protein CG bead was within a distance of up to 6 Å from any POPC bead. The total contact counts were divided by the number of frames to report an averaged value per frame.

VMD^91^ was used for the visualisation of all systems. Ad hoc Python 3 scripts (containing MDAnalysis v2.2.0^92^ and NumPy libraries) were used during post-processing and data analysis. Matplotlib^93^ was used for plotting.

### Sequence retrieval and analysis

Gene sequences for the following proteins from *A. thaliana* were used in this study for experimental or computational analysis: Toc75 (At3g46740), Toc159 (At4g02510), Toc33 (At1g02280), Toc34 (At5g05000), Toc120 (At3g16620), Toc132 (At2g16640), Tic214 (AtCg01130), Tic100 (At5g22640), Tic56 (At5g01590), Tic110 (At1g06950), Tic40 (At5g16620), LHCB1.1 (At1g29920), Actin2 (At3g18780), Rubisco small subunit SSU (At1g67090), and pyruvate dehydrogenase E1α subunit (At1g01090). Gene sequences for the following proteins from *C. reinhardtii* were used in this study for computational analysis: Toc75 (Cre03.g175200), Toc90 (Cre17.g734300), and Toc34 (Cre06.g252200).

Sequences were obtained from the TAIR (http://www.arabidopsis.org/), Phytozome (https://phytozome-next.jgi.doe.gov/), UniProt (https://www.uniprot.org/), or National Centre for Biotechnology Information (NCBI) (https://www.ncbi.nlm.nih.gov/) databases.

Multiple sequence alignments were performed using Clustal Omega (https://www.ebi.ac.uk/jdispatcher/msa/clustalo) using default settings^94^.

### Statistics and reproducibility

Statistical analyses were performed using Microsoft Excel (Microsoft 365 Apps for enterprise, Version 2601, Microsoft Corporation, USA). Two-tailed or one-tailed unpaired Student’s *t* tests were performed. *p-*value ranges are shown in the relevant figure legends, and all the individual *p-*values are included in the Source Data file. *p-*values < 0.05 were considered to indicate significant difference (*); *p-* values > 0.05 indicated no significant difference (ns). Data were plotted as mean ± SEM from at least three independent experiments.

### Reporting summary

Further information on research design is available in the Nature Portfolio Reporting Summary linked to this article.

## Supplementary information

Supplementary Information

Description of Additional Supplementary Files

Supplementary Data 1

Supplementary Data 2

Supplementary Data 3

Supplementary Data 4

Supplementary Movie 1

Supplementary Movie 2

Reporting Summary

Transparent Peer Review file

## Source data

Source Data

## Data Availability

All data generated or analysed during this study are included in this published article or its supplementary information, apart from the following. The mass spectrometry proteomics data have been deposited to the ProteomeXchange Consortium via the PRIDE partner repository^95,96^; specifically, the HA-Toc75 proteomic data (experiments i, ii and v in the Methods) and the crosslinking mass spectrometry data (experiments iii and iv in the Methods) have been deposited under accession codes PXD072672 and PXD064453, respectively (https://www.ebi.ac.uk/pride/archive/projects/). Coordinates and molecular dynamics parameters from the molecular dynamics simulations have been deposited in the Zenodo repository under the accession code 18467186. All unique materials are readily available from the authors. Source data are provided in this paper.

## Rights retention statement

This research was funded in whole or in part by UKRI (BB/F020325/1, BB/K018442/1, BB/N006372/1, BB/R009333/1, BB/R016984/1, BB/V007300/1, BB/W015021/1, BB/X000192/1, BB/Z516624/1 and UKRI719). For the purpose of Open Access, the author has applied a CC BY public copyright licence to any Author Accepted Manuscript (AAM) version arising from this submission.
