# Supplementary material for: Assembly of two functionally-distinct protein import complexes in the outer membrane of plant chloroplasts
Source: Nat Commun. 2026 Apr 20;17:5433. doi: 10.1038/s41467-026-71676-6 (PMC13279931; doi:10.1038/s41467-026-71676-6)

SUPPLEMENTARY INFORMATION FOR:

Assembly of two functionally-distinct protein import complexes in the outer membrane of plant chloroplasts

Sreedhar Nellaepalli[1#*], Domagoj Baretić[2#], Astrid F. Brandner[3], Sybille Kubis-Waller[4,5], Duorong Xu[1], Ziad Soufi[1], Shuyang Cheng[1], Sireesha Kodru[1], Jun Fang[1], Ursula Flores-Perez[1], Vaishnavi Ravikumar[3], Pablo Pulido[1,6], Marjorie Fournier[3], Ivan Ahel[2], Syma Khalid[3], and R. Paul Jarvis[1,*]

[1]Section of Molecular Plant Biology, Department of Biology, University of Oxford, Oxford OX1 3RB, UK
[2]Sir William Dunn School of Pathology, University of Oxford, Oxford OX1 3RE, UK
[3]Department of Biochemistry, University of Oxford, Oxford OX1 3QU, UK
[4]Department of Biology, University of Leicester, Leicester LE1 7RH, UK
[5]Present address: MRC Laboratory of Molecular Biology, Cambridge CB2 OQH, UK
[6]Department of Plant Molecular Genetics, Centro Nacional de Biotecnologia, Consejo Superior de Investigaciones Cientıficas (CNB-CSIC), 28049 Madrid, Spain
[#] Equal authorship
*Correspondence: sreedhar.nellaepalli@biology.ox.ac.uk and paul.jarvis@biology.ox.ac.uk

SUPPLEMENTARY INFORMATION

Supplementary information for this article includes:

Supplementary results

Properties of the GTPase heterodimer in the receptor module

Unique features of the Chlamydomonas TOC receptors

Supplementary figures (1-20)

Supplementary Fig. 1. Analysis of TOC complex components in petals.

Supplementary Fig. 2. Identification of polypeptides in affinity-purified HA-Toc75 preparations by silver staining and mass spectrometry.

Supplementary Fig. 3. Visible phenotypes of the *toc* mutants used in this study.

Supplementary Fig. 4. Analysis of TOC component gene expression in the *toc* mutants.

Supplementary Fig. 5. Comparison of the AF3 outputs for TOC-P and TOC-P subcomplexes.

Supplementary Fig. 6. Negative-staining EM and XL-MS analysis of the purified TOC-P complex.

Supplementary Fig. 7. AF3 analysis of TOC-N reveals similarities to, and differences from, TOC-P.

Supplementary Fig. 8. Two GTP-binding pockets exist at the interface of the GTPase heterodimer formed between Toc33 and Toc159.

Supplementary Fig. 9. Similarity of the Toc33-Toc159 GTPase heterodimer to the crystal structure of a pea Toc34 homodimer.

Supplementary Fig. 10. Molecular dynamics simulations reveal the membrane contact sites of cytosolic regions of Toc159.

Supplementary Fig. 11. Structural organization of the heterodimeric β-barrel.

Supplementary Fig. 12. Toc75 acts as a central hub of interactions with Toc33 and Toc159.

Supplementary Fig. 13. The C-terminal transmembrane helix of Toc159 is required for stabilization of Toc159 in the membrane.

Supplementary Fig. 14. Analysis of TOC-TIC interactions in *A. thaliana* reveals that the two plant complexes are separable.

Supplementary Fig. 15. Analysis of *TOC* gene expression in the two *HA-Toc75* over-expressor lines.

Supplementary Fig. 16. Ratiometric imaging analysis reveals that transit peptide identity influences chloroplast protein import efficiency in vivo.

Supplementary Fig. 17. Phyre2 identifies similarity between the Toc159 β-barrel and the mitochondrial protein import channel Tom40.

Supplementary Fig. 18. Mass spectra identifying TOC crosslinks.

Supplementary Fig. 19. AF3 predictions of TOC complexes.

Supplementary Fig. 20. AlphaLink2 predictions of the TOC-P.

SUPPLEMENTARY RESULTS

Properties of the GTPase heterodimer in the receptor module

In the GTP binding pockets of the TOC-P receptor module, Toc33-GTP2/GTP1 hydrogen-bond interactions (Toc33-GTP2: KSS [aa 49-51], H160, N209; Toc33-GTP1: R130) and Toc159-GTP1/GTP2 hydrogen-bond interactions (Toc159-GTP1: G865, GKSA [aa 867-870], S889, H982, N1036; Toc159-GTP2: D989) are at highly conserved positions **(Supplementary Fig. 8d,e)**. This implies that the two GTPs likely play a role in the dimerization process. Of these residues, Toc33-R130 was previously implicated in homodimerization in vitro, potentially acting as an arginine finger with a catalytic role in GTP hydrolysis[1-3]. In fact, the AF3-predicted GTPase heterodimer is similar overall to the crystal structure of a homodimer of *Pisum sativum* Toc34 formed in vitro, providing further strong support for the veracity of the AF3 model[2] **(Supplementary Fig. 9)**. However, the physiological function of Toc33/Toc34 homodimers in vivo, if any, is not clear.

Unique features of the Chlamydomonas TOC receptors

While the structural organization of the TOC complex is broadly conserved in plants and green algae **(Fig. 6a,c)**, closer scrutiny of the *C. reinhardtii* TOC GTPase domains missing from the cryo-EM structures did reveal two distinct characteristics: (i) Toc34 features an additional, short N-terminal region (1-59 aa, pI = 3.38) that is acidic in nature **(Fig. 6d)**; (ii) whereas the N-terminus of Toc90 is, conversely, basic in nature (1-189 aa, pI = 10.9). In contrast, land plants such as Arabidopsis lack an acidic domain in Toc33/34, and instead possess large N-terminal domains in the Toc159-type receptors that are acidic in nature (for Toc159, 1-781 aa, pI = 3.98). Moreover, three alga-specific TOC subunits (Toc52, Toc39 and Toc10) were previously identified in *C. reinhardtii*[4]. However, functional analyses are required to better understand the particular properties of the algal TOC complex.

SUPPLEMENTARY FIGURES

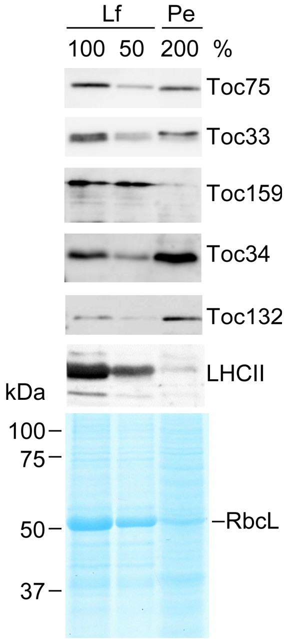

Supplementary Fig. 1. Analysis of TOC complex components in petals.

Total protein extract from the petals (Pe) of 6-week-old WT plants was analysed by immunoblotting alongside the chloroplast-enriched leaf fraction (Lf) described in **Fig. 1e**. The samples were loaded on a tissue fresh weight basis. Twice the amount (200%) of the petal sample was loaded relative to the chloroplast-enriched sample, so as to achieve normalized levels of the Toc75 component, and of the overall proteome as revealed by Coomassie brilliant blue (CBB) staining. Two different loading amounts (%) of the Lf sample are shown.

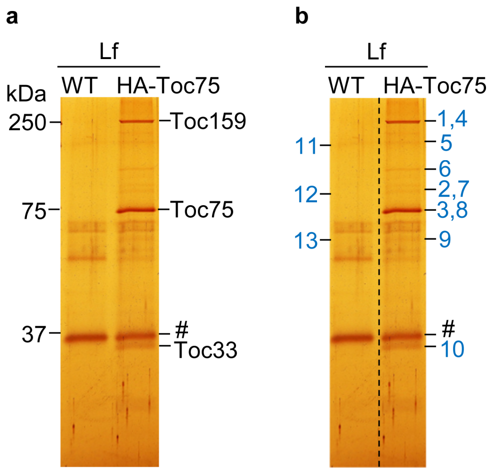

Supplementary Fig. 2. Identification of polypeptides in affinity-purified HA-Toc75 preparations by silver staining and mass spectrometry.

The main components of the purified TOC-P complex were clearly visible by silver staining (**a**). These and other strongly-staining abundant polypeptides in the gel were excised (see numbers in **b**), digested with trypsin, and subjected to LC-MS/MS analysis **(Supplementary Data 1)**. Note that bands annotated as 1-13 here correspond to SN1-SN13, respectively, in the data file. In parallel, HA-peptide eluted samples were subjected directly to in-solution LC-MS/MS analysis **(Supplementary Data 1)**. Overall, the data show that HA-Toc75 purifications from leaf extracts are enriched in Toc159 and Toc33 by comparison with Toc132/-120 and Toc34, respectively. The image in **b** is identical to that in **a**, only with additional annotations. A non-specific band is indicated (#).

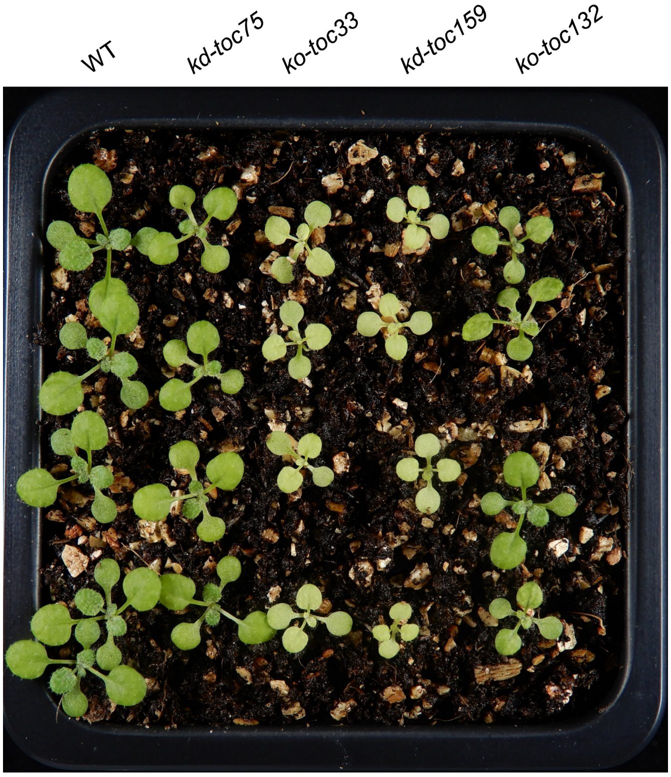

Supplementary Fig. 3. Visible phenotypes of the *toc* mutants used in this study.

The *kd-toc75*, *ko-toc33*, *kd-toc159* and *ko-toc132-2* mutants were grown alongside WT plants under identical conditions. The plants were photographed at the age of three weeks. Other names used previously to describe these mutants are as follows: *mar1/toc75-III-3* (*kd-toc75*), *ppi1-1* (*ko-toc33*), *fts1/ppi2-3* (*kd-toc159*), and *toc132-2* (*ko-toc132*).

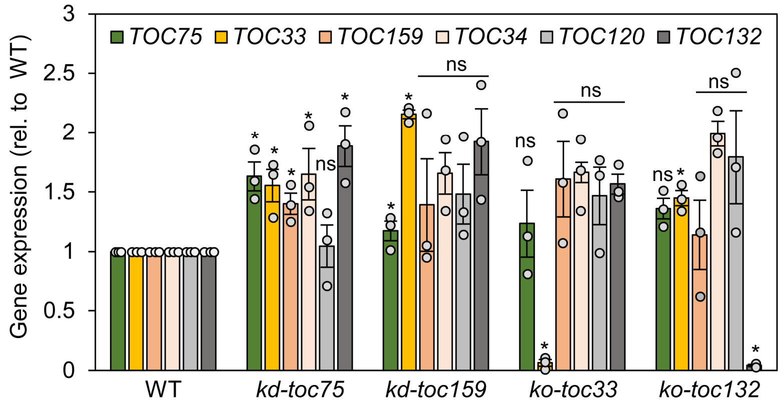

Supplementary Fig. 4. Analysis of TOC component gene expression in the *toc* mutants.

Gene expression analysis was performed by qRT-PCR using RNA samples extracted from 2-week-old plants of the indicated genotypes. Expression data for *TOC* genes were normalized using data for *ACTIN2*. All values are means ± SEM (n = 3 experiments). Asterisks indicate significance according to paired one-tailed Student's t tests comparing the *toc* mutant lines with WT (*p < 0.048; ns, not significant). The data show that *TOC* genes (other than those directly affected by the mutation) are not generally under-expressed in *toc* mutants. The *TOC75* mutation in *kd-toc75* (causing missense mutation G658R) and the *TOC159* mutation in *kd-toc159* (causing premature stop at codon 1472) do not negatively affect transcription levels of *TOC75* and *TOC159*, respectively; though these mutations do strongly affect protein accumulation **(Fig. 2)**.

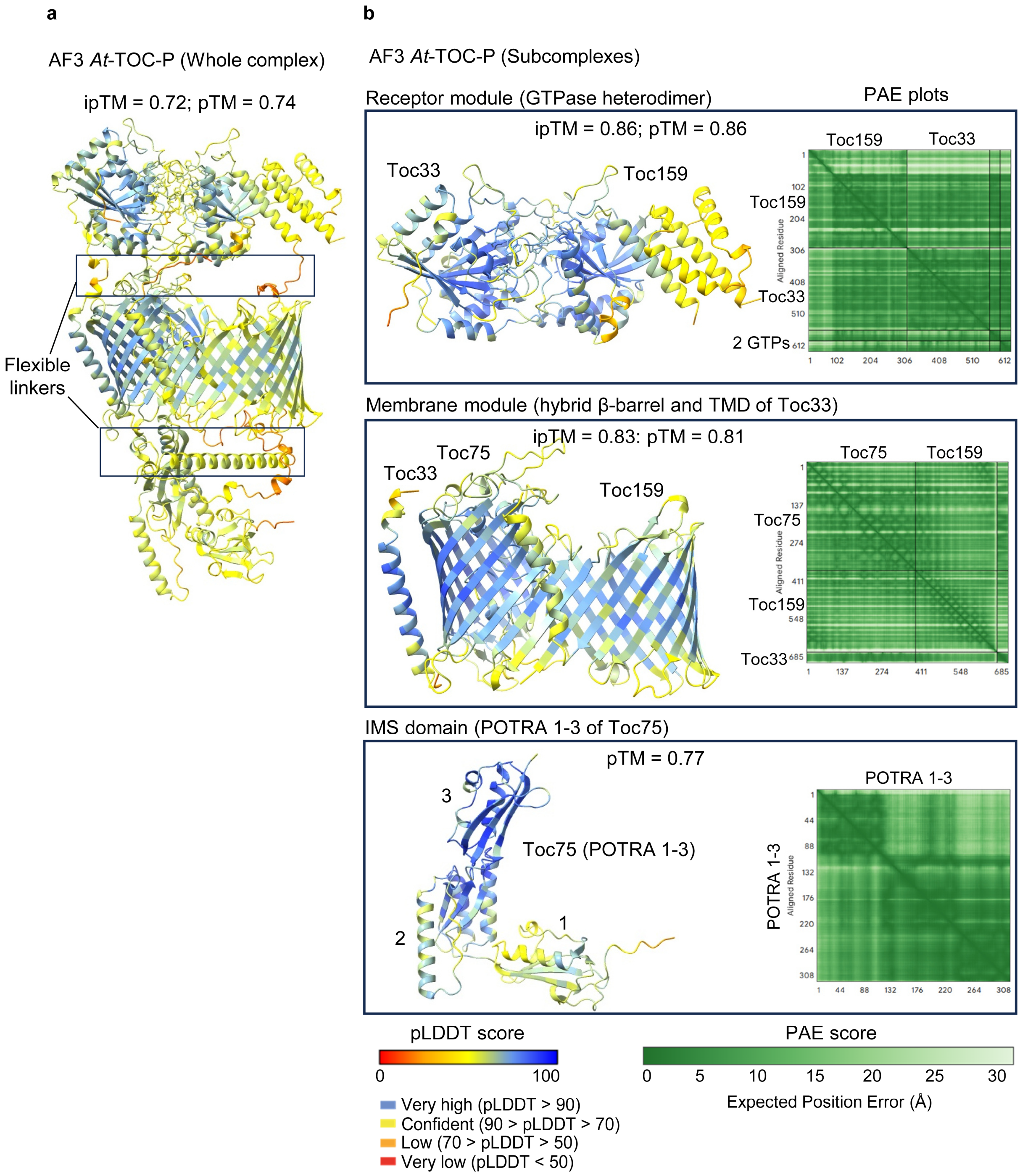

Supplementary Fig. 5. Comparison of the AF3 outputs for TOC-P and TOC-P subcomplexes.

a. AF3 prediction of the whole TOC-P complex. Flexible regions that negatively influence the confidence scores are highlighted.

b. Separate AF3 predictions of each TOC-P subcomplex. From top to bottom: receptor module (Toc33-Toc159 GTPase heterodimer); membrane module (Toc33-Toc75-Toc159 membrane domains); IMS domain (POTRA 1-3 of Toc75). The polypeptide chains are coloured according to pLDDT scores. The 2D plots show predicted alignment error (PAE) scores.

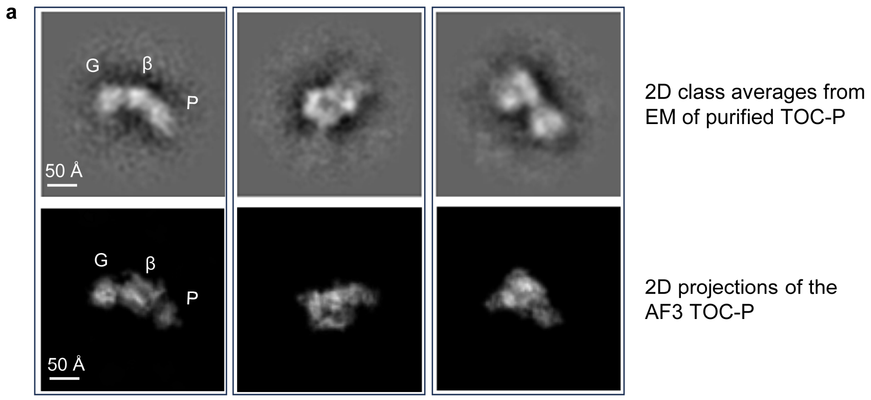

b

No	Crosslinks	TOC subunit	Linkage mapping (XL in red)	Linkage (aa–aa)	Cα–Cα distance (Å)
1	SLMGSVTTSNFLNPQDDLSFKLEYVHPYLDGVYNPR-AGVKANITENFTR	Toc75	β[3-4]-6β	373-426	11.263
2	AGVKANITENFTR-DVKGNPTAVYR	Toc75	6β-L-β[13-14]	426-630	26.766
3	ELETLATCGMFEKVDLEGK-DQGKVSAR	Toc75	POTRA1-POTRA2	84-179	19.353
4	KALSNINSLGLFSNIEVNPRPDEK-DNTKFVNGAVVGQR	Toc75	POTRA3-L-β[8-9]	271-500	15.927
5	AGVKANLTENFTR–TFKTSCFNSR	Toc75	6β-5β	393-426	8.129
6	DNTKFVNGAVVGQR–HQLTMTKFLQLR	Toc75	10β-L-β(8-9)	500-537	11.709
7	HQLTMTKFIQLR-DNTKFVNGAVVGQR	Toc75	L-β(8-9)-10β	537-500	11.709
8	TVFQVDQGLGLGSKFPFFNR–DVKGNPTAVYR	Toc75	L-β[13-14]-L-β[9-10]	524-630	14.320
9	DNTKFVNGAVVGQR–ELPKQLR	Toc75	POTRA3-L-β[8-9]	256-500	21.175
10	KALSNINSLGLFSNIEVNPRPDEK-ELPKQLR	Toc75	POTRA3-POTRA3	256-271	10.645
11	KLSPVFTGGPGVEEVPPIWVDR-AGVKANITENFTR	Toc75	5β-6β	401-426	17.613
12	QSKFTYGLVMEEITTR(3)-AGVKANITENFTR	Toc75	7β-6β	426-438	17.614
13	TGQGSSYGAGVKLGLVR-DVKGNPTAVYR	Toc75	14β-L-β[13-14]	630-651	40.051
14	AGLNNKMSGQLTVR–NSKLALR	Toc159	L-β[13-14]-14β	668-678	31.369
15	AGLNNKMSGQLTVR–KVFGFR	Toc159	L-β[13-14]-Linker-2	307-678	39.880
16	SIRPEATNDKYSMY-KVFGFR	Toc159	C-term-Linker-2	307-719	52.266
17	SNIFSNSNVTMADETEINLSEEEKQK-LEKLQSLRVKFLR	Toc159	α*-α*	4-16	18.397
18	SNIFSNSNVTMADETEINLSEEEKQK-LEKLQSLRVKFLR	Toc159	α*-α*	4-9	8.679
19	SNIFSNSNVTMADETEINLSEEEKQKLEK-LQSLRVKFLR	Toc159	α*-α*	6-16	15.389
20	NSKLALR–KVFGFR	Toc159	14β-Linker-2	307-668	55.850
21	KQEFEDSAIAVVYAENSGR-SDSLLKTIR	Toc33	α5'-α5	184-194	10.289

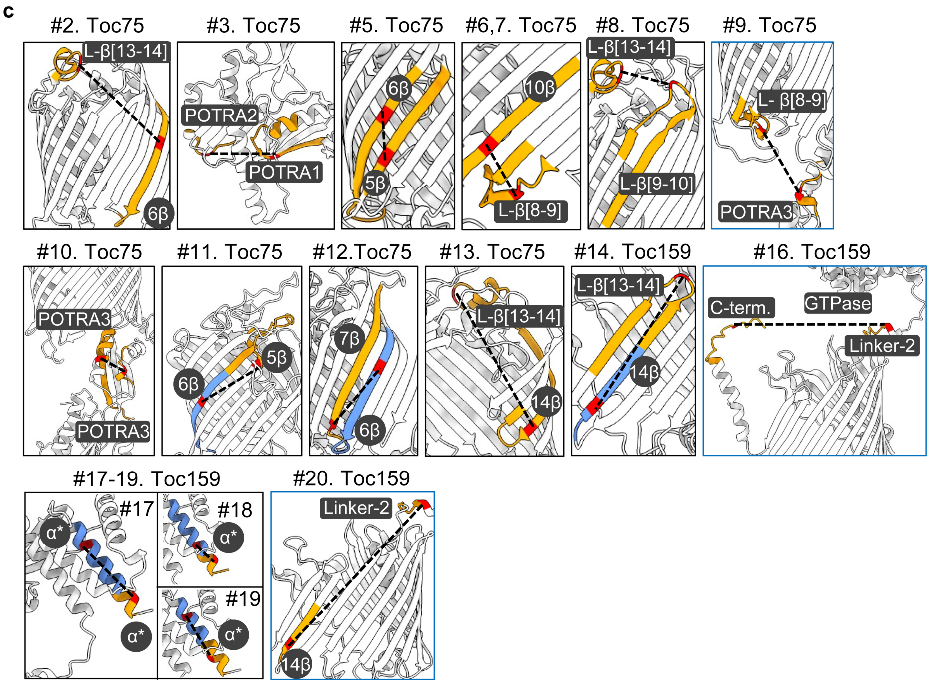

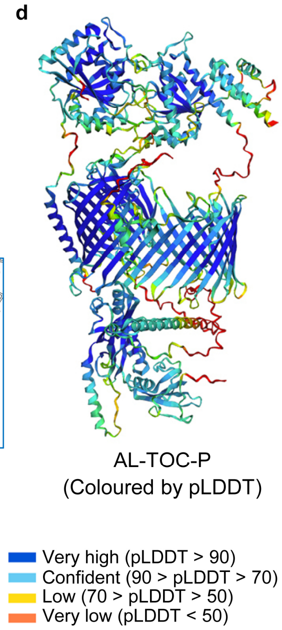

Supplementary Fig. 6. Negative-staining EM and XL-MS analysis of the purified TOC-P complex.

a. Negative staining EM analysis of the TOC-P complex. Affinity-purified TOC-P complex was separated by size exclusion chromatography and the resulting peak fraction was analysed by negative staining and EM. Single particles from the EM images were picked and used to generate 2D class averages. In parallel, 2D projections of the AF3 TOC-P model **(see Fig. 3a-d)** were generated by CryoSPARC, and these were matched with the 2D class average images from the EM analysis. Note that purified HA-Toc75 complexes from chloroplast extracts are clearly enriched in TOC-P, with TOC-N present only at negligible levels. Thus, the negative-staining results highly likely represent the enriched TOC-P complex. See also **Fig. 3e**.

b,c. Crosslinking-mass spectrometry (XL-MS) analysis of the TOC-P complex. Affinity-purified HA-Toc75 samples were crosslinked using 0.5 mM BS3 for 30 min or 3 mM BS3 for 3 h. The resulting samples were subsequently digested in-gel or in-solution with trypsin and subjected to LC-MS/MS analysis. The identified crosslinked peptides are shown, with linkage sites (lysines) highlighted in red (**b**). For a full list of identified peptides, please refer to **Supplementary Data 2**. The identified crosslinks were mapped onto the TOC-P structure **(see also Fig. 3f)**. In each image, crosslinked parts of the structure are highlighted (yellow for peptides, red for residues; for #11, #12, #14 and #17-19, one peptide is in blue as the two peptides are contiguous), with the crosslinks shown by the dashed black lines. Interdomain crosslinks (#9, #16, #20) are marked with blue boxes. Linker-2 connects the GTPase and β-barrel domains of Toc159. L, loop.

d. Structure of TOC-P as predicted by Alphalink2 (AL-TOC-P). The Alphalink2 model shown is identical to the one presented in **Fig. 3g**, except that here it is coloured according to pLDDT model confidence scores. As noted before, the model satisfies the majority of experimentally detected crosslinks. Overall AL-TOC-P model confidence, 0.71; pTM, 0.72; ipTM, 0.70; crosslink satisfaction, 0.79; mean distance of crosslinked residues, 23.35 Å.

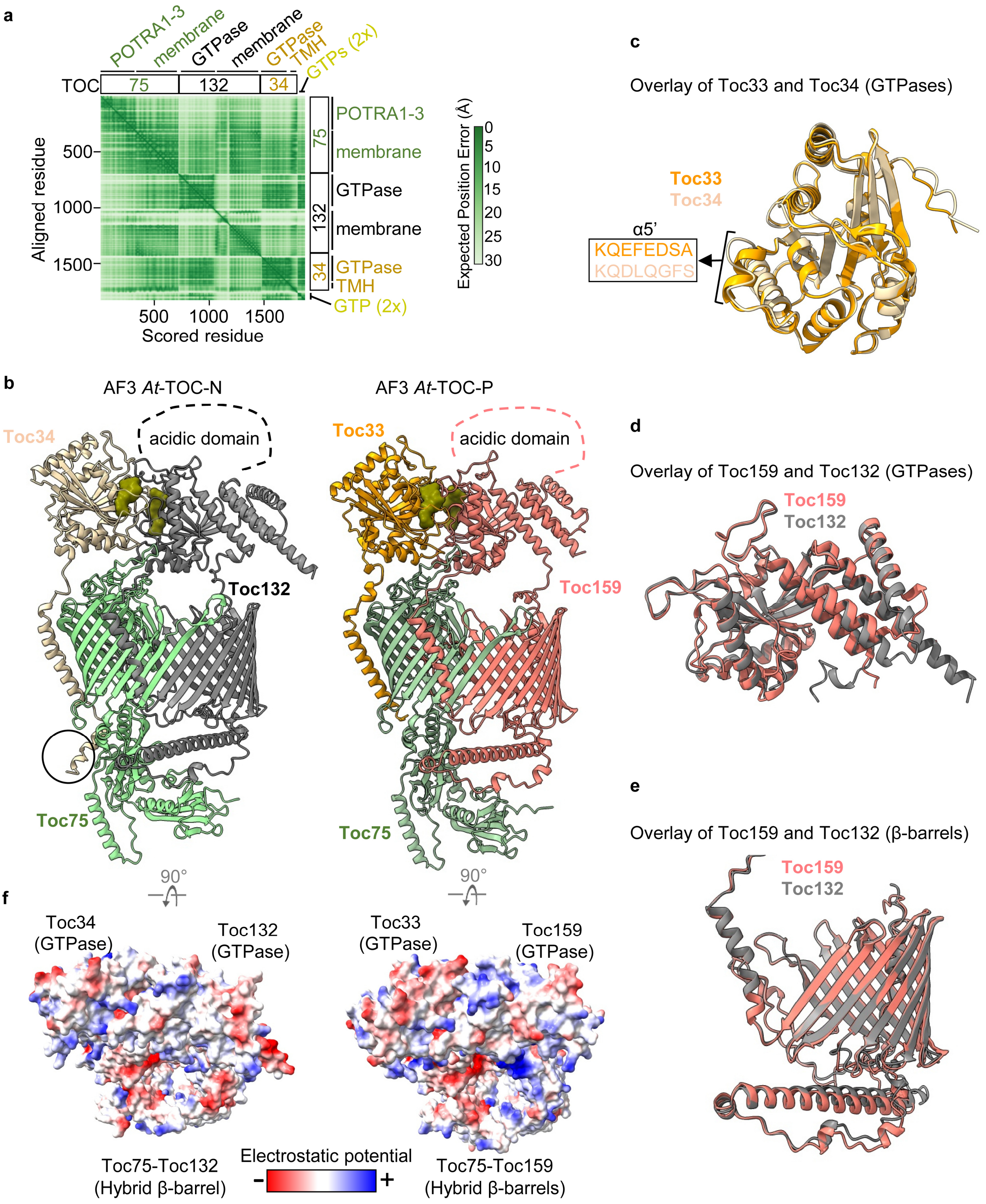

Supplementary Fig. 7. AF3 analysis of TOC-N reveals similarities to, and differences from, TOC-P.

a. Predicted alignment error (PAE) plot from the AF3 analysis of TOC-N components and two GTPs. This analysis was conducted in exactly the same manner as the similar analysis of TOC-P **(see Fig. 3)**. The sequences of Toc75 and Toc132 were N-terminally truncated to remove transit peptide and disordered A domain sequences, respectively; the submitted sequences were 678 (Toc75), 720 (Toc132), and 313 (Toc34) residues long.

b. Predicted structure of the TOC-N complex from AF3 (left side), alongside the similarly-generated TOC-P complex structure **(see Fig. 3c)** for comparison (right side). The circle

shows an extended helix at the C-terminus of Toc34. Positions of the acidic A domains of Toc159 and Toc132 (which were excluded because of their intrinsically disordered nature) are shown by the dashed lines. The two GTPs (depicted as surface models) are shown in olive green.

c. Superposition of the predicted structures of the GTPase domains of Toc33 and Toc34 (rotated 90° relative to panel **b**). The assignment of α5' was according to a published component structure (PDB: 1H65)[63].

d. Superposition of the predicted structures of the GTPase domains of Toc159 and Toc132.

e. Superposition of the predicted structures of the β-barrel domains of Toc159 and Toc132.

f. Surface electrostatic potential representations of the TOC-N and TOC-P structures. Both images are top-down views from the cytosol. There are some notable differences between the two complexes. In the TOC-P complex, the negative potential on the upper surface of Toc33 and the inner surface of the Toc75 β-barrel is counterbalanced by the positive/neutral (non-polar) potential on the upper surface of Toc159 and, especially, on the inner surface of the Toc159 β-barrel. This configuration may facilitate efficient routing of preproteins, enabling the heavy traffic associated with importing photosynthetic preproteins. In contrast, the TOC-N complex exhibits a weaker gradation of surface electrostatic potentials, suggesting a slower import process that is perhaps adequate or better suited for housekeeping preproteins. Calculated pI values of the transit peptides of the two preproteins assessed in this study (SSU and E1α) **(see Fig. 10)** are 10.05 and 12.13, respectively.

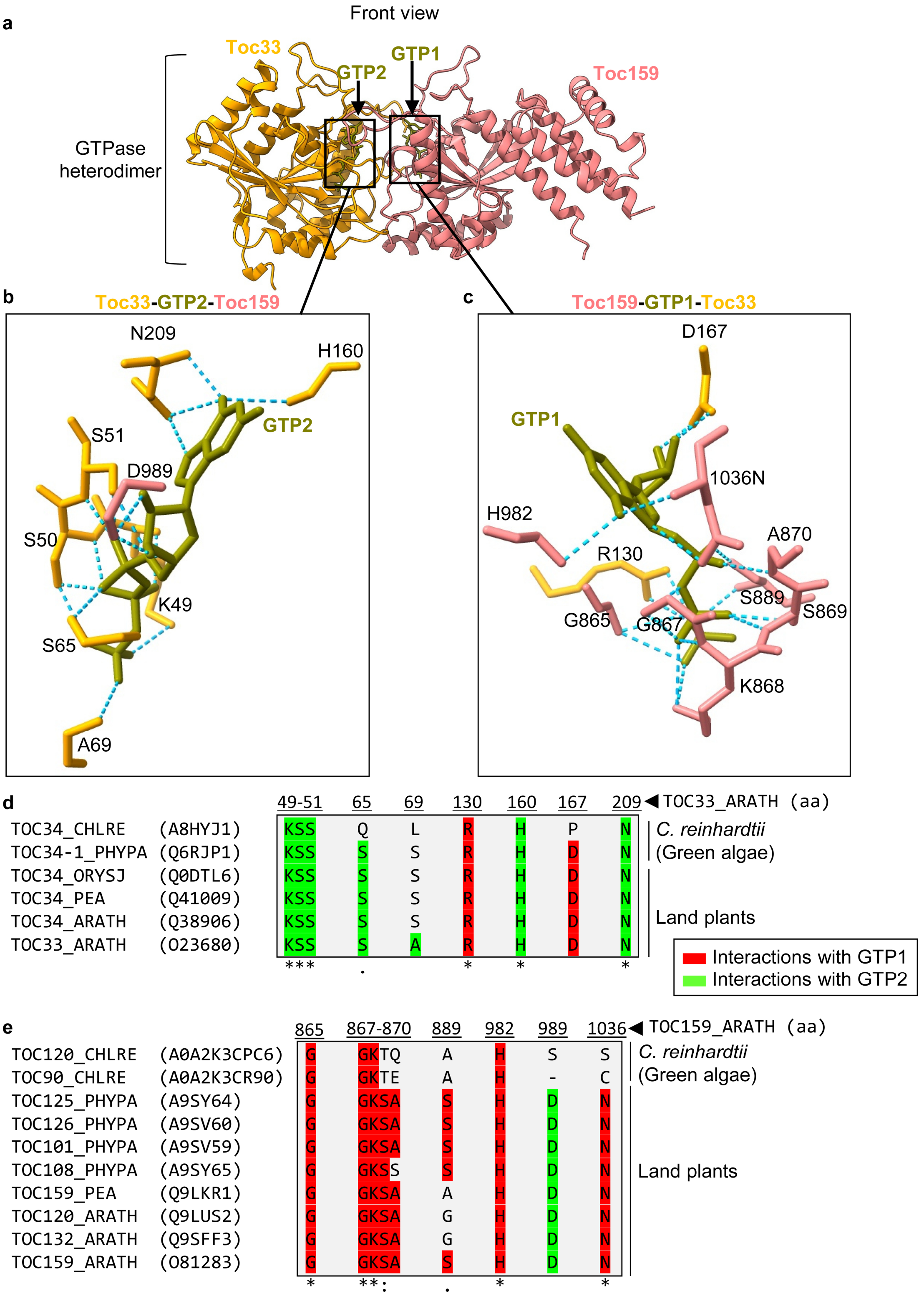

Supplementary Fig. 8. Two GTP-binding pockets exist at the interface of the GTPase heterodimer formed between Toc33 and Toc159.

a. Structure of the GTPase heterodimer (front view), showing how the two GTPs lie at the interface of the dimer. GTP2 is principally associated with Toc33, whereas GTP1 is principally associated with Toc159. Boxes show the two GTP-binding pockets.

b,c. Hydrogen-bond interactions in the two GTP-binding pockets. Interaction between Toc33, GTP2 and Toc159 (**b**), and between Toc159, GTP1 and Toc33 (**c**) are shown by the blue dotted lines. Both GTPases contribute to the binding of each GTP.

d,e. Multiple sequence alignments (MSAs) showing conservation of the GTP-interacting amino acids of the receptor GTPases. Toc33-sequences were obtained from UniProt for *C. reinhardtii* (A8HYJ1, TOC34_CHLRE), *Physcomitrium patens* (Q6RJP, TOC34-1-PHYP), *A. thaliana* (O23680, TOC33_ARATH; Q38906, TOC34_ARATH), *Oryza sativa* (Q0DTL6, TOC34_ORYSJ), and *P. sativum* (Q41009, TOC34_PEA) (**d**). Toc159-related sequences were obtained from UniProt for *A. thaliana* (Q6S5G3, TOC90_ARATH; O81283, TOC159_ARATH; Q9LUS2, TOC120_ARATH, Q9SFF3, TOC132_ARATH), and *P. patens* (A9SY64, TOC125_PHYPA; A9SV60, TOC126_PHYPA; A9SV59, TOC101_PHYPA; A9SY65, TOC108_PHYPA) (**e**). Complete sequences were aligned in each case, but for simplicity only GTP-interacting amino acids are shown here (red, GTP1; green, GTP2). All residues shown in **d** and **e** are proximal to the interface of the GTPase heterodimer. Clustal Omega was used to perform both MSAs[5], and the GTP-interacting residues were copied out manually. Coordinates at the top show positions in the *Arabidopsis* Toc33 (**d**) and Toc159 (**e**) proteins, corresponding to residues marked in **b** and **c**, respectively. The symbols at the bottom (*:.) indicate the degree of conservation at each position.

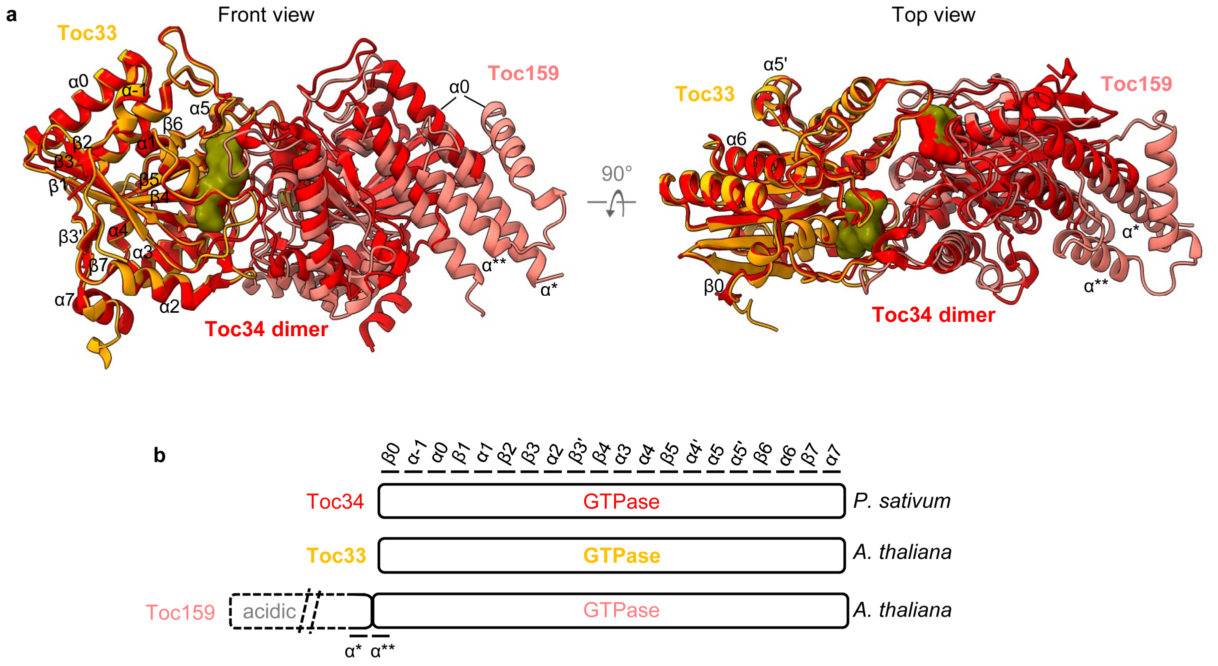

Supplementary Fig. 9. Similarity of the Toc33-Toc159 GTPase heterodimer to the crystal structure of a pea Toc34 homodimer.

a. Superposition of the GTPase heterodimer from the AF3-generated TOC-P structure with the published crystal structure of a *P. sativum* Toc34 homodimer (PDB: 1H65)[2]. *Arabidopsis* Toc33 and Toc159 are shown in orange and salmon pink, respectively; the pea Toc34 homodimer is shown in red; and the two GTPs (depicted as surface models) are shown in olive green. Left side, front view; right side, top view.

b. Secondary structural elements of the *P. sativum* Toc34 GTPase domain as previously described[2]. These structural elements were largely conserved in the GTPase domains of Toc33 and Toc159. Two upstream sequences of the Toc159 GTPase domain were additionally predicted by AF3 to be α-helices, as shown (α*, α**). These two helices may protect the folded GTPase structure from the lengthy intrinsically-disordered acidic domain of Toc159.

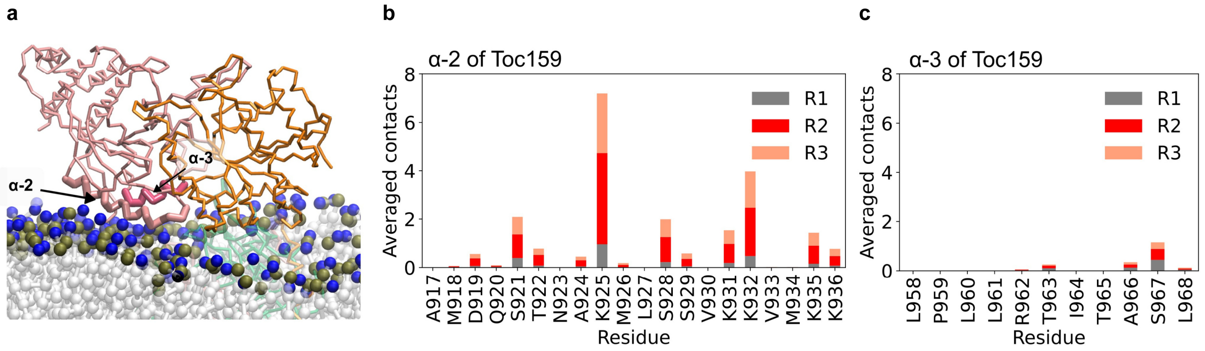

Supplementary Fig. 10. Molecular dynamics simulations reveal the membrane contact sites of cytosolic regions of Toc159.

a. Membrane-contacting cytosolic regions of Toc159 (pink) and Toc33 (orange) are shown. The helices α-2 (salmon pink) and α-3 (dark pink) of Toc159 are depicted with thick lines. POPC molecules are shown in light grey, with their headgroup moieties represented as coloured spheres (phosphate, khaki green; choline, blue).

b,c. Analysis of the lipid-α-2 and lipid-α-3 contacts. Bar charts showing averaged contacts between the α-2 or α-3 helices of Toc159 and POPC. The total contact counts for each residue along the whole production run were divided by the number of frames, producing an average value per frame. A cut-off of 0.6 nm was used to define a direct contact between any protein and POPC bead.

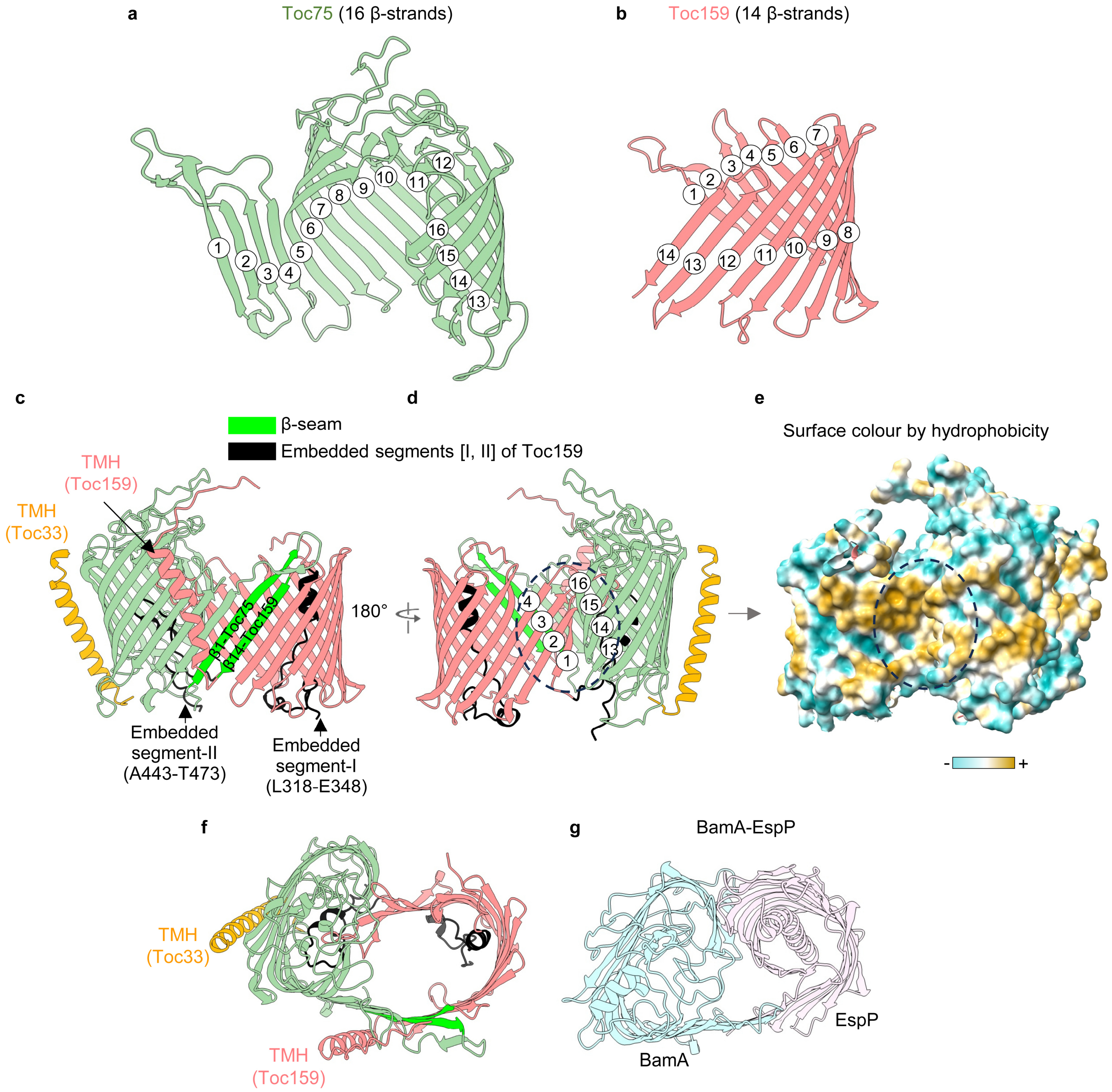

Supplementary Fig. 11. Structural organization of the heterodimeric β-barrel.

a,b. Structures of the Toc75 and Toc159 β-barrel domains (each taken from the TOC-P complex model) are shown in isolation, to more clearly reveal the arrangement of the individual β-strands (16 in Toc75, 14 in Toc159). The images shown are front view (Toc159) or 90° rotation of front view (Toc75).

c. At the front of the hybrid barrel, the β-sheet domains of Toc75 and Toc159 are connected by a β-seam between β1 of Toc75 and β14 of Toc159. The two embedded segments of Toc159 are shown in black; embedded segment-II lies within the Toc75 barrel.

d. At the other side of the barrel (back), the β-sheet domains of Toc75 (strands β13-β16) and Toc159 (strands β1-β4) curl inwardly to close the barrel.

e. The model shown in panel **d** is here surface coloured according to hydrophobicity. Negative (cyan) and positive (yellow) values indicate hydrophilic and hydrophobic patches, respectively. The dashed circle indicates a hydrophobic groove where Toc75 and Toc159 curl inwards and close the β-barrel with weak van der Waals interactions.

f. Top-down view of the hybrid barrel showing how the transmembrane helices (TMH) of Toc33 and Toc159 are in close proximity to the barrel on two faces of the complex. The Toc159 TMH traverses the β-seam and potentially acts to stabilize the hybrid barrel.

g. For comparison, the published structure of a hybrid β-barrel assembly complex formed between bacterial BamA and its substrate protein EspP (PDB: 8BNZ) is shown[32]. This is structurally similar to the Toc75-Toc159 hybrid barrel presented in **f**.

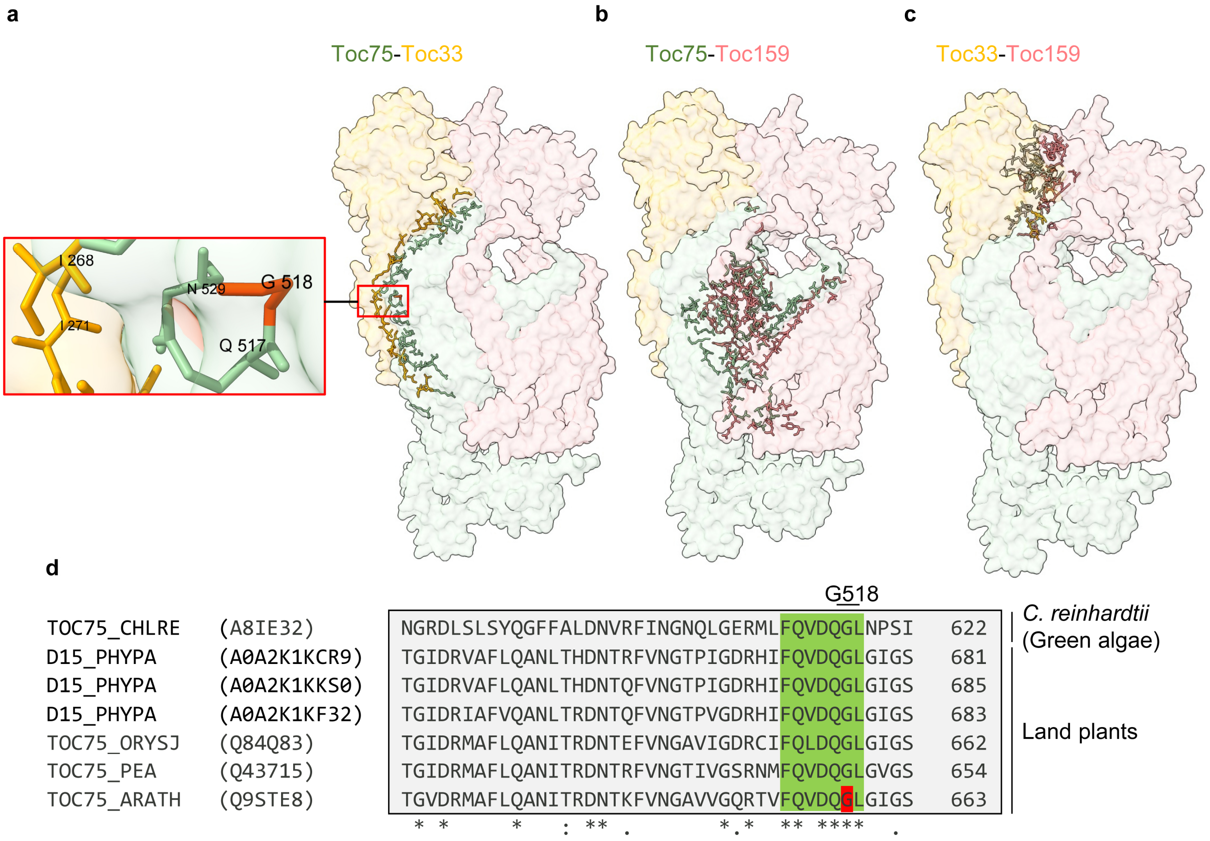

Supplementary Fig. 12. Toc75 acts as a central hub of interactions with Toc33 and Toc159.

a-c. Surface models of the TOC-P complex (front view) that have been annotated to highlight residues participating in inter-subunit interactions. Interacting residues are shown in stick model format with darker colours. The models show that Toc75-Toc33 contacts occur in the membrane and cytosolic regions (**a**); that Toc75-Toc159 contacts are numerous in the membrane region (**b**); and that Toc33-Toc159 interactions are limited to the cytosolic region, between the GTPase domains (**c**). The inset in **a** shows the position of a Toc75 missense mutation present in the *kd-toc75* (*mar1/toc75-III-3*) mutant (G518R of mature Toc75; G658R of the precursor); the G518 residue is highlighted in dark orange/red. This mutation destabilizes the TOC-P and TOC-N complexes, as shown in **Fig. 2**.

d. Multiple sequence alignment (MSA) of Toc75-related sequences showing conservation of glycine 518. Toc75-related sequences were obtained from UniProt for *C. reinhardtii* (A8IE32; TOC75_CHLRE), *P. patens* (A0A2K1KCR9, D15_PHYPA; A0A2K1KKS0, D15_PHYPA; A0A2K1KF32, D15_PHYPA), *O. sativa* (Q84Q83, TOC75_ORYSJ), *P. sativum* (Q43715, TOC75_PEA), and *A. thaliana* (Q9STE8, TOC75-3_ARATH). Complete sequences were aligned in each case, but for simplicity only regions of interest are shown here. Clustal Omega was used to perform the MSA[64], and the relevant regions were copied out manually. Coordinates at right show positions in each protein sequence; symbols at the bottom (*:.) indicate the degree of conservation at each position. It is evident that G518 (red) of *A. thaliana* Toc75 is highly conserved, and present in a conserved motif (green box). This is in accordance with the observation that G518 is important for stabilization of the TOC complexes **(Fig. 2)**.

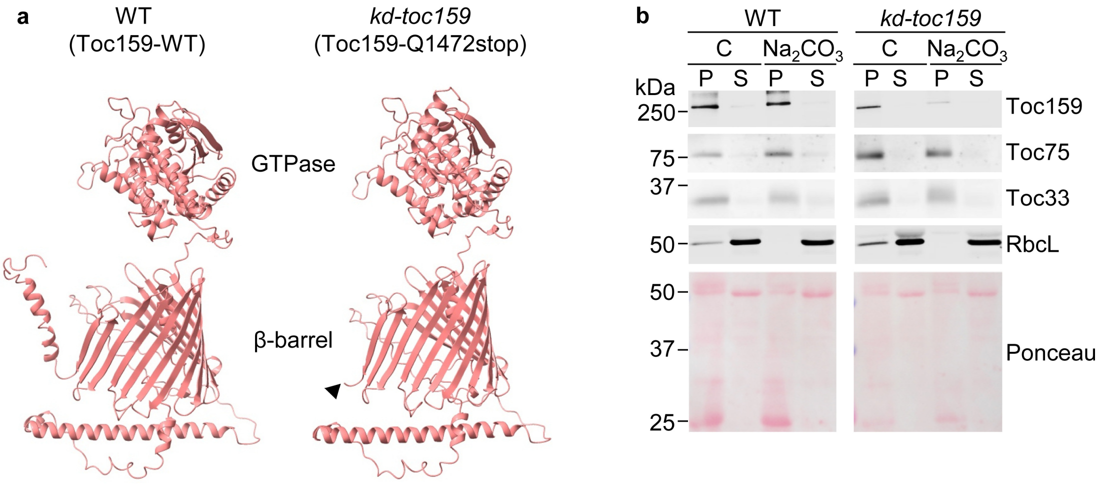

Supplementary Fig. 13. The C-terminal transmembrane helix of Toc159 is required for stabilization of Toc159 in the membrane.

a. In the TOC-P structure, Toc159 is comprised of a cytosolic GTPase domain, a membrane-embedded β-barrel domain, and two additional alpha helices at the membrane: the first lies along the membrane facing towards the intermembrane space, and the second is a C-terminal transmembrane helix (TMH). The C-terminal TMH (32 residues in length) is absent in the *kd-toc159* (*fts1/ppi2-3*) mutant because of a mutation causing a premature stop codon (Q1472stop). The black triangle indicates the position of the truncation.

b. Stability of the mutant Toc159 protein is substantially compromised. Chloroplast membrane fractions from WT and *kd-toc159* plants were subjected to alkaline treatment (100 mM Na_2CO_3) for 30 min (or without alkaline treatment as a control, C); and then the samples were separated into membrane pellet (P) and soluble (S) fractions. The fractions were analysed by immunoblotting. The mutant Toc159 protein (Q1472stop) was extracted much more readily than the WT protein, and compared to Toc75 and Toc33, indicating instability.

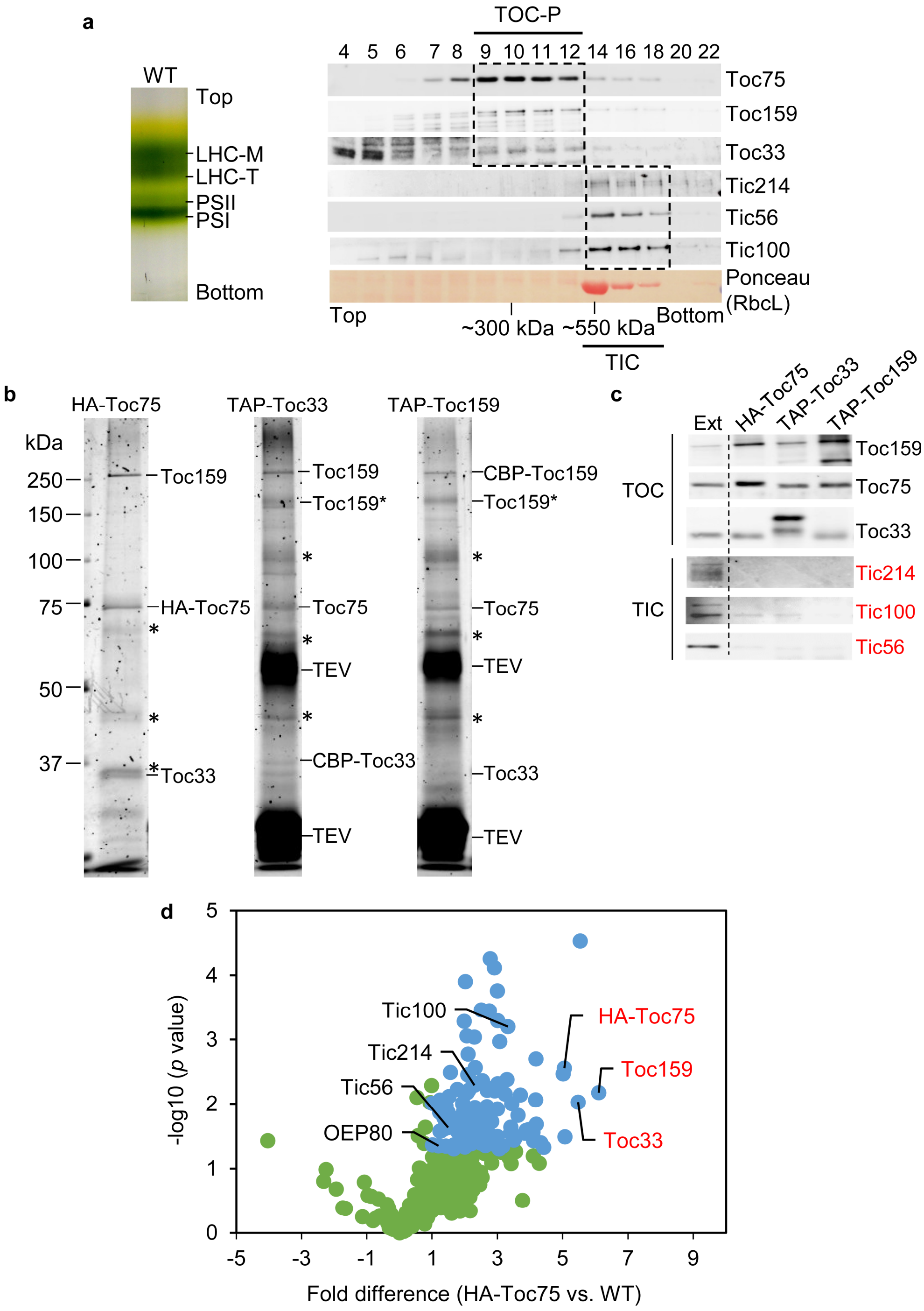

Supplementary Fig. 14. Analysis of TOC-TIC interactions in *A. thaliana* reveals that the two plant complexes are separable.

a. Sucrose density gradient ultracentrifugation analysis of protein complexes in a chloroplast-enriched leaf fraction from WT plants. The gradient showed clear separation of pigment-protein complexes (left). Fractions from the gradient were analysed by immunoblotting and Ponceau staining (to detect RbcL) (right), enabling resolution of TOC-P (left) and TIC (right).

b. Staining analysis of affinity-purified TOC complexes from three different transgenic lines. Chloroplast-enriched samples were prepared from 2-week-old transgenic plants expressing HA-tagged Toc75 (*HA-Toc75* #1) or tandem-affinity-purification (TAP)-tagged Toc33 or Toc159. Samples were solubilized with 1% β-DM to extract membrane protein complexes before purification. The TAP tag carries two affinity domains (calmodulin-binding peptide

(CBP), and the terminal immunoglobulin G (IgG)-binding Protein A domain) separated by a tobacco etch virus (TEV) protease cleavage site. Excess TEV protease was used to elute the TAP samples after Protein A-based purification, and the protease is clearly present in the relevant samples including in aggregated form. Resolved polypeptides were visualized by Flamingo fluorescent staining. Asterisks indicate non-specific interactions, whereas Toc159* indicates a common proteolytic fragment of Toc159. Positions of molecular weight markers (sizes in kDa) are indicated at left. Staining at the expected positions for prominent TIC components was not detected.

c. Immunoblotting analysis of the affinity-purified TOC complex samples. Samples similar to those shown in **b** were analysed by immunoblotting using antibodies against TOC and TIC components. Whereas the TOC components were all clearly present as expected, the TIC components were either completely absent or present at negligible levels. Ext indicates a comparable extract from WT plants (not subjected to affinity purification) for comparison.

d. Volcano plot showing label-free quantitative mass-spectrometry analysis of affinity-purified HA-Toc75 samples. Triplicate samples were digested on-bead with trypsin prior to LC-MS/MS analysis **(Supplementary Data 3)**. Proteins significantly enriched (t test, p <0.05; fold change [FC] >1) relative to WT negative control samples are highlighted in blue. The main TOC components were highly enriched. The analysis also detected TIC components, albeit at much lower levels. Maximum peptide spectral match (PSM) values for TOC and TIC components were as follows: Toc75 (186), Toc159 (127), Toc33 (37), Tic214 (17), Tic100 (2), Tic56 (3).

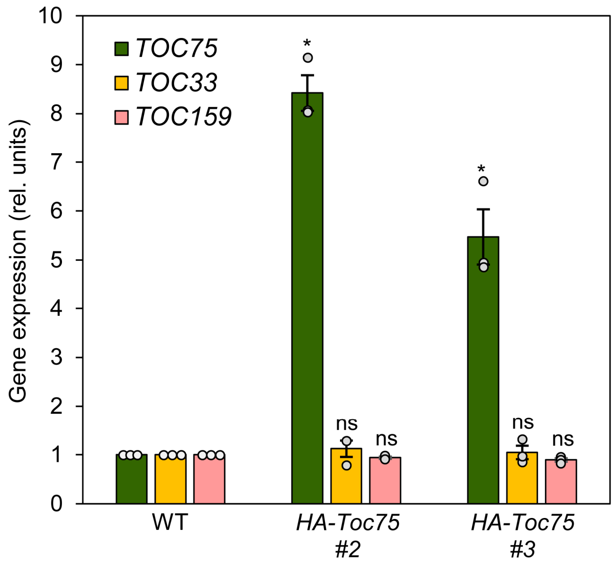

Supplementary Fig. 15. Analysis of *TOC* gene expression in the two *HA-Toc75* over-expressor lines.

Gene expression analysis was performed by qRT-PCR using RNA samples extracted from 2-week-old plants. The relevant transgenic plant lines were identified in a screen for complemented *HA-Toc75* lines, and are also presented in **Fig. 1b-d** (lines #2 and #3). Expression data for *TOC* genes were normalized using data for *ACTIN2*. Asterisks indicate significance according to paired one-tailed Student's t tests comparing the transgenic lines with WT (*$p < 0.01$; ns, not significant). All values are means ± SEM (n = 3 experiments). The data show that *TOC* genes (apart from *TOC75*) are not generally overexpressed in the *HA-Toc75* lines.

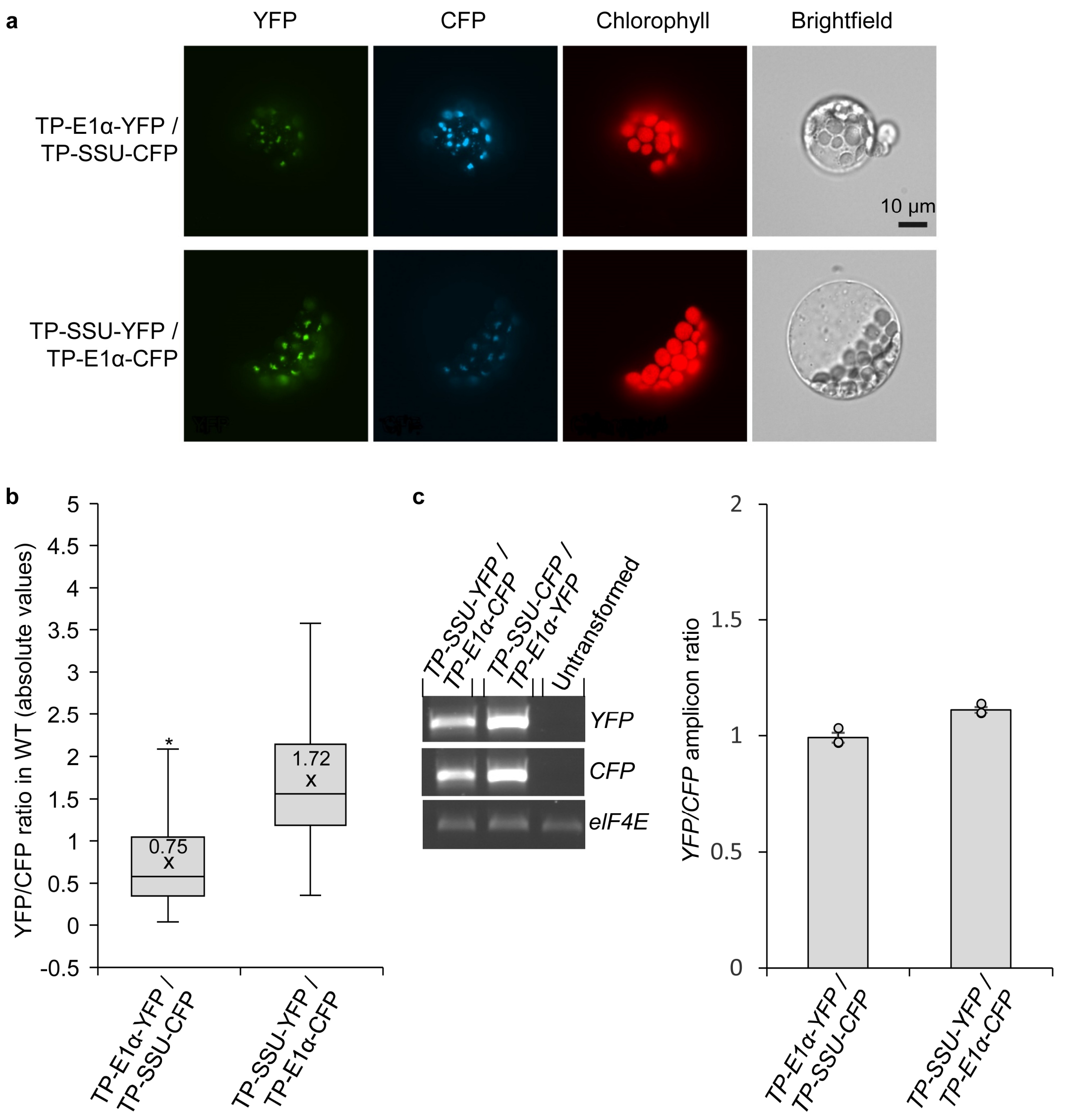

Supplementary Fig. 16. Ratiometric imaging analysis reveals that transit peptide identity influences chloroplast protein import efficiency in vivo.

a. Qualitative analysis of the relative chloroplast-targeting efficiencies of the E1α and SSU transit peptides. Protoplasts from WT were co-transfected with plasmids encoding the indicated transit peptide (TP) fusions, and fluorescence microscopy was used to detect the transiently-expressed proteins. The YFP (green; left panels) and CFP (cyan; centre-left panels) fluorescence signals were recorded, alongside chlorophyll autofluorescence (red; centre-right panels) and brightfield images (right panels). Each set of images is of the same cell, and is representative of a large number of images derived from four independent experiments.

b. Quantitative analysis of the experiments presented in **a**. The YFP/CFP fluorescence intensity ratios within individual chloroplasts were quantified using image analysis software; the data values shown here are absolute ratio values that have not been normalized. For each combination of constructs, the data shown comprise ~1000-2000 individual ratio measurements, derived from a total of up to 59 different protoplasts from four separate experiments. Asterisks indicate significance according to two-tailed Student's t tests comparing TP-E1α-YFP / TP-SSU-CFP with TP-SSU-YFP / TP-E1α-CFP (*$p < 0.001$). In the

box-whisker plot, the box, centre line, and cross-mark indicate interquartile range, median, and mean, respectively; outlier points are not shown (n = 1201-1865).

c. Analysis of the gene expression of the transfected plasmids in **a** and **b**. Samples of protoplasts that were either untransformed or had been co-transfected with the construct-pairs described above were subjected to RT-PCR analysis using construct-specific primers; similar analysis using *eIF4E1*-specific primers provided a positive control for the untransformed sample. Control PCR reactions containing the RNA samples as template (i.e., no reverse transcription) produced no amplification using the construct-specific primers. Typical stained agarose gels are shown at left, while quantitative analysis of the relevant amplicons in repeated experiments is shown at right. Corresponding values for *YFP* and *CFP* were used to calculate *YFP/CFP* ratios, which were then used to calculate means. Presented values are mean ratios ± SEM (n = 5 experiments). The data show that the two transfected genes (*YFP* and *CFP*) were expressed at a similar level in co-transfected cells of the same experiment.

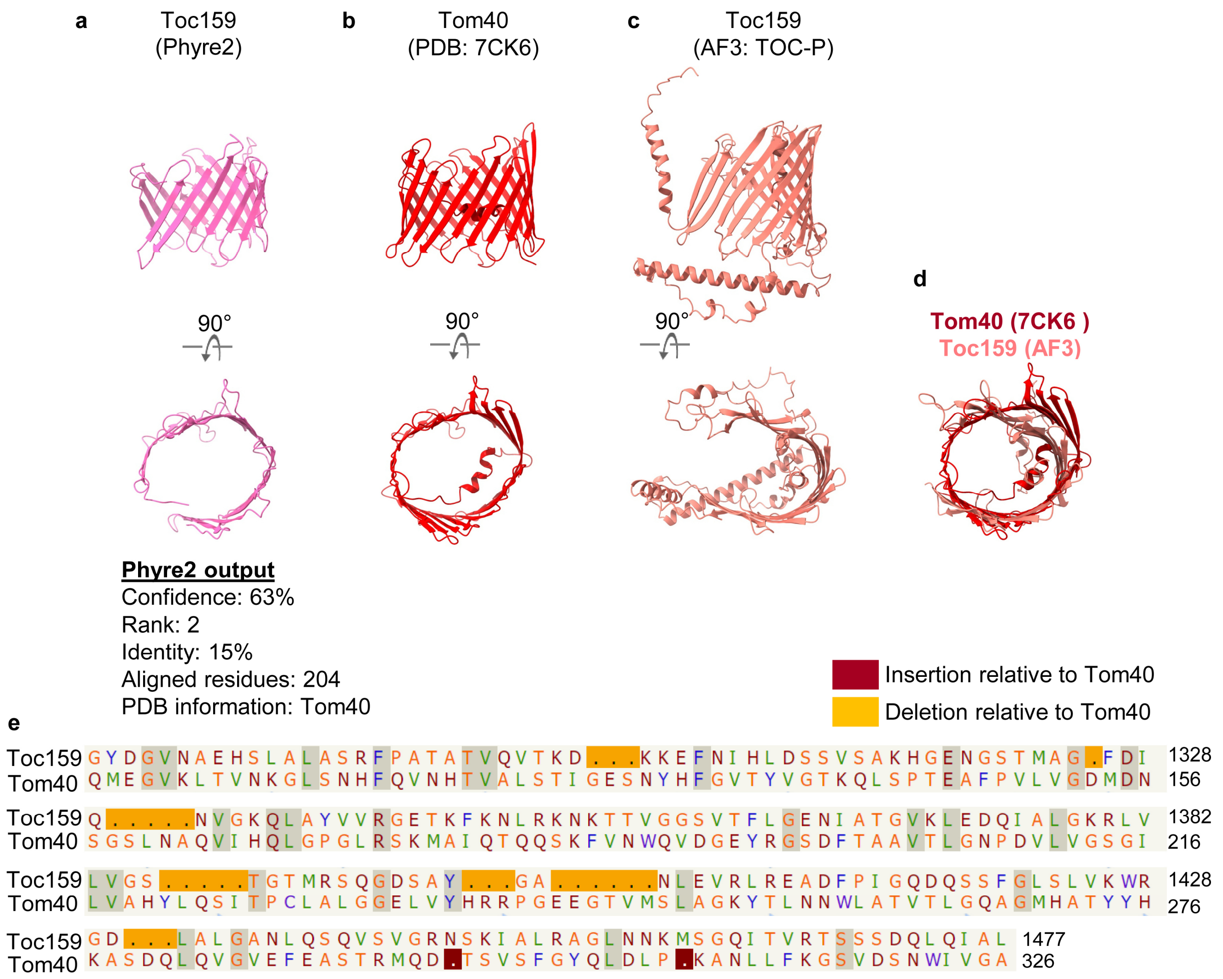

Supplementary Fig. 17. Phyre2 identifies similarity between the Toc159 β-barrel and the mitochondrial protein import channel Tom40.

The amino acid sequence of the membrane β-barrel domain of Toc159 (residues 1045-1503) was submitted to the Phyre2 protein threading server[7] to identify similar database structures. The second most similar hit identified (the first one being a homologous chloroplast translocon receptor from algae) was the mitochondrial protein import channel Tom40 (PDB: 7CK6). We compared the Toc159 structural model built by Phyre2 (using the Tom40 structure as a template; confidence, 63%) (**a**) with the original Tom40 structure (**b**), and with the membrane domain of Toc159 from our AF3-generated TOC-P structure **(see Fig. 3)** (**c**). In each case, front and top-down views are shown; outputted data from Phyre2 are also shown in **a**. Superposition (top view only) of the Tom40 structure with our AF3-generated Toc159 β-barrel structure supports the structural similarity identified by Phyre2 (**d**). The amino acid sequence alignment generated by Phyre2, covering the area of Toc159-Tom40 structural similarity, is also shown (**e**); identical residues are shaded. Together, these analyses revealed that the overall β-sheet folding pattern within the β-barrel domain of Toc159 (14 strands) is similar to that seen in the larger β-barrel of Tom40 (19 strands), in relation to both the arrangement of the β-strands and the size and arrangement of the inter-strand loops. It is noteworthy that both barrels (Toc159 and Tom40) have an open structure in the sense that the channel openings are not occluded.

Supplementary Fig. 18. Mass spectra identifying TOC crosslinks.

The MS/MS spectra shown correspond to crosslinks between two peptides within Toc75 (#1-13), Toc159 (#14-20), or Toc33 (#21). The crosslink spectra of #5, #6, #8 and #15 are from MetaMorpheus 1.0.9, and all others are from pLink 3.

Please note that the spectra appear at the end of this document.

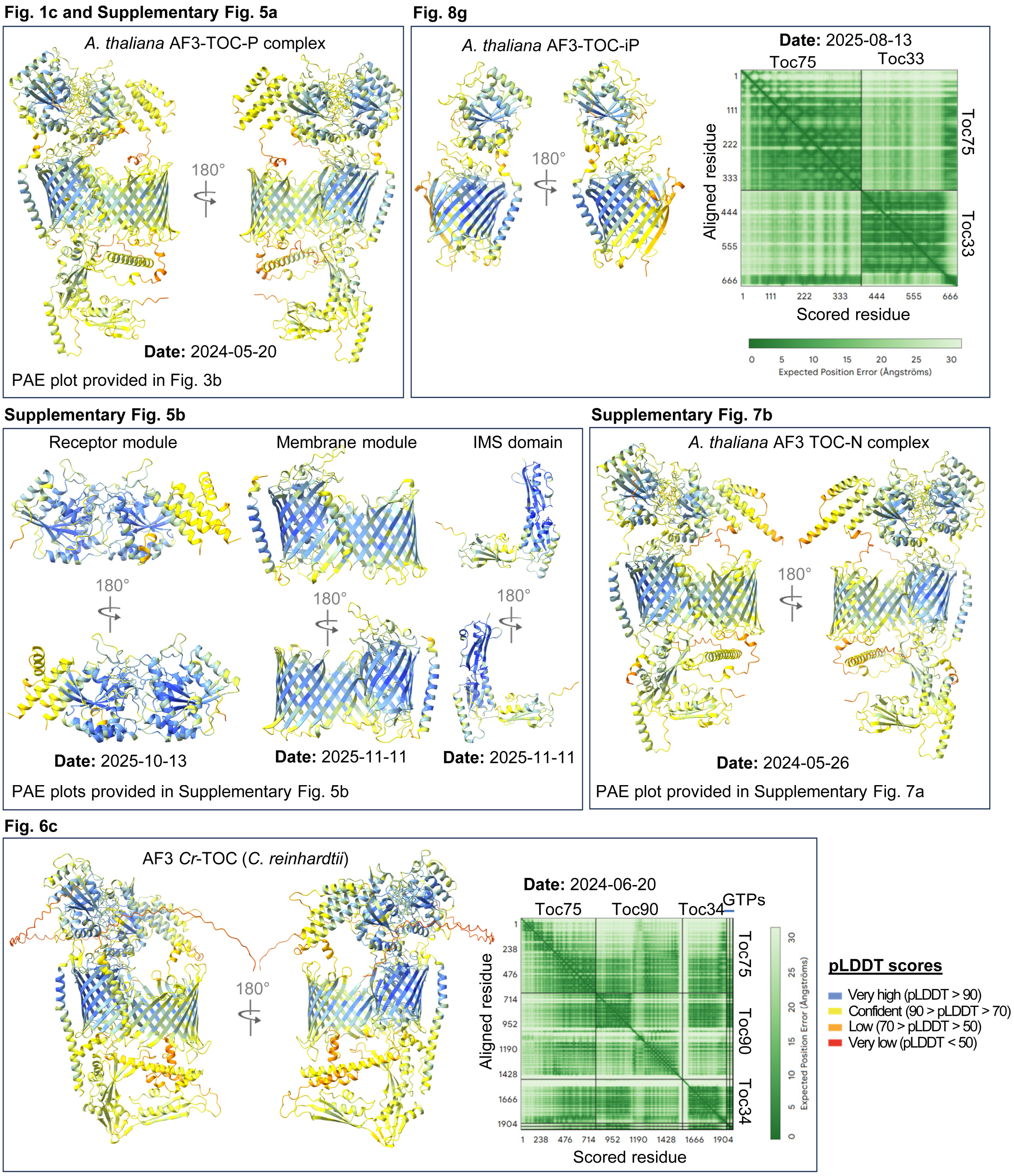

Supplementary Fig. 19. AF3 predictions of TOC complexes.

These data support the figures indicated over each panel. The predictions (shown from two viewing angles) are coloured according to their pLDDT scores. PAE plots are shown for the TOC-iP and *Cr*-TOC complexes.

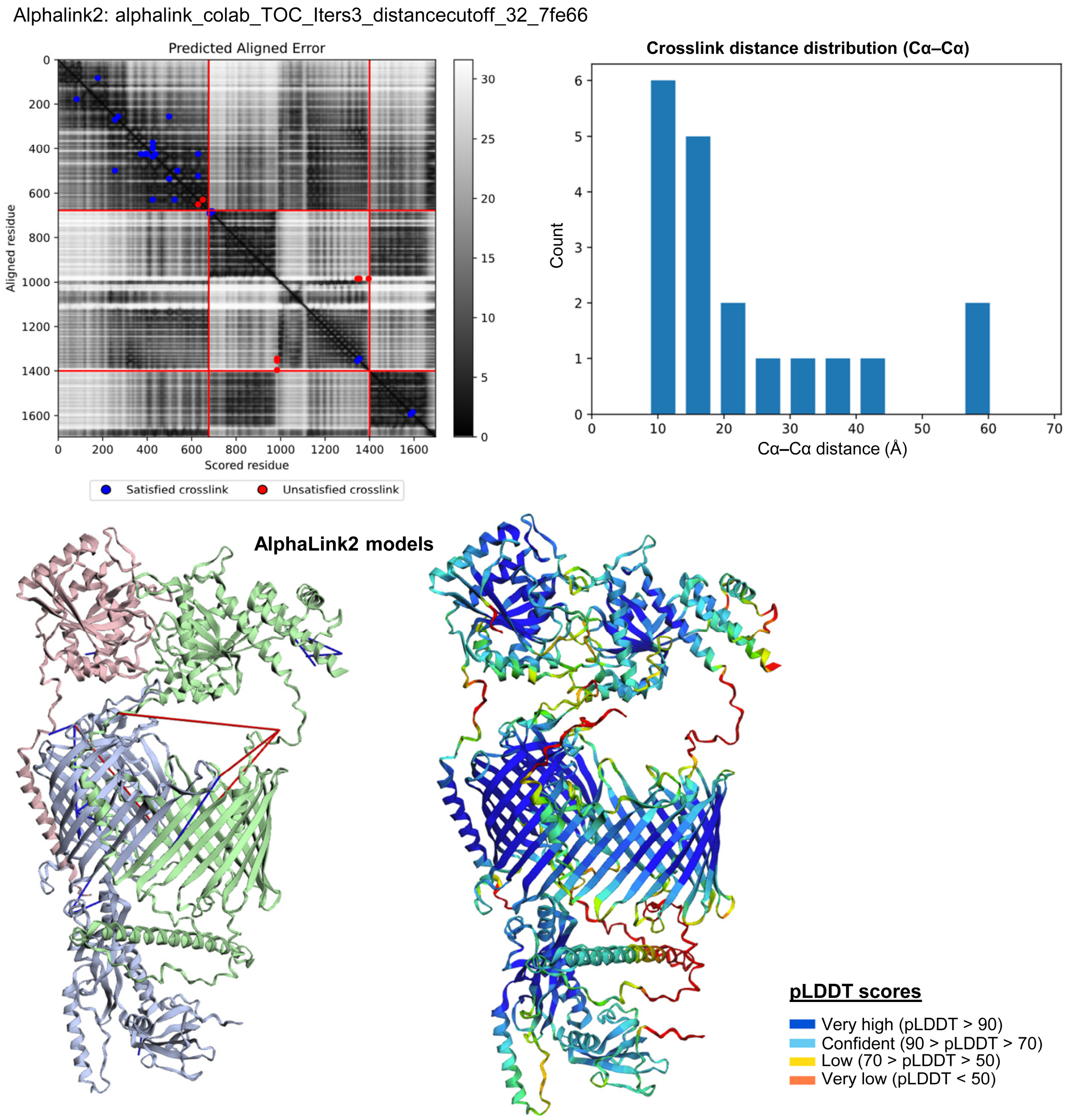

Supplementary Fig. 20. AlphaLink2 prediction of the TOC-P complex.

These data support **Fig. 3g** and **Supplementary Fig. 6d.** The chemical crosslinks (blue dots [satisfied], red dots [unsatisfied]) are shown in the PAE plot. Crosslink distance distribution (Cα-Cα) is shown in the bar chart. The AlphaLink2 model is coloured according to polypeptide chain (left) (Toc33, pink; Toc159, green; Toc75, grey blue) and pLDDT scores (right); crosslinks are marked in the former following the colour coding used in the PAE plot.

REFERENCES

1. Koenig, P. et al. The GTPase cycle of the chloroplast import receptors Toc33/Toc34:: Implications from monomeric and dimeric structures. *Structure* **16**, 585–596 (2008).
2. Sun, Y.J. et al. Crystal structure of pea Toc34, a novel GTPase of the chloroplast protein translocon. *Nat. Struct. Biol.* **9**, 95–100 (2002).
3. Weibel, P., Hiltbrunner, A., Brand, L. & Kessler, F. Dimerization of Toc-GTPases at the chloroplast protein import machinery. *J. Biol Chem.* **278**, 37321–37329 (2003).
4. Jin, Z.Y. et al. Structure of a TOC-TIC supercomplex spanning two chloroplast envelope membranes. *Cell* **185**, 4788–4800 (2022).
5. Sievers, F. et al. Fast, scalable generation of high-quality protein multiple sequence alignments using Clustal Omega. *Mol. Syst. Biol.* **7**(2011).
6. Liu, H., Li, A., Rochaix, J.D. & Liu, Z. Architecture of chloroplast TOC-TIC translocon supercomplex. *Nature* **615**, 349–357 (2023).
7. Kelley, L.A., Mezulis, S., Yates, C.M., Wass, M.N. & Sternberg, M.J. The Phyre2 web portal for protein modeling, prediction and analysis. *Nat. Protoc.* **10**, 845–58 (2015).

Supplementary Fig. 18 (part 1)

Toc75 (Crosslink #1)

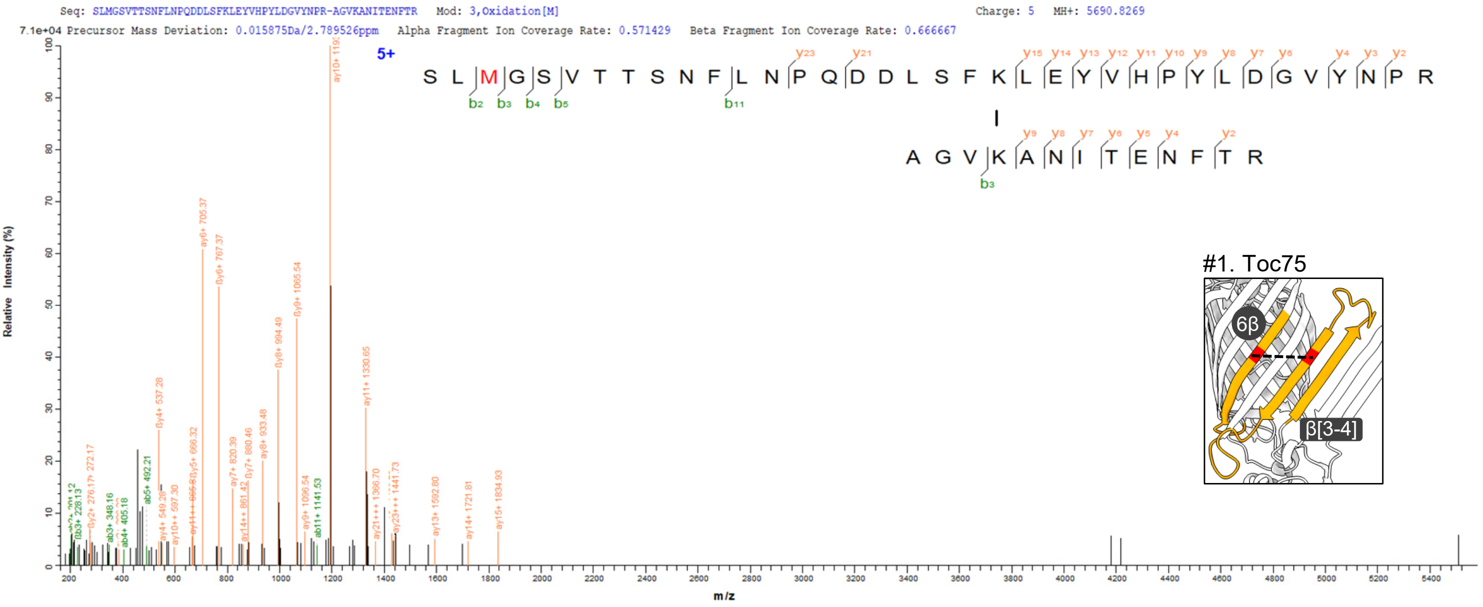

Toc75 (Crosslink #2)

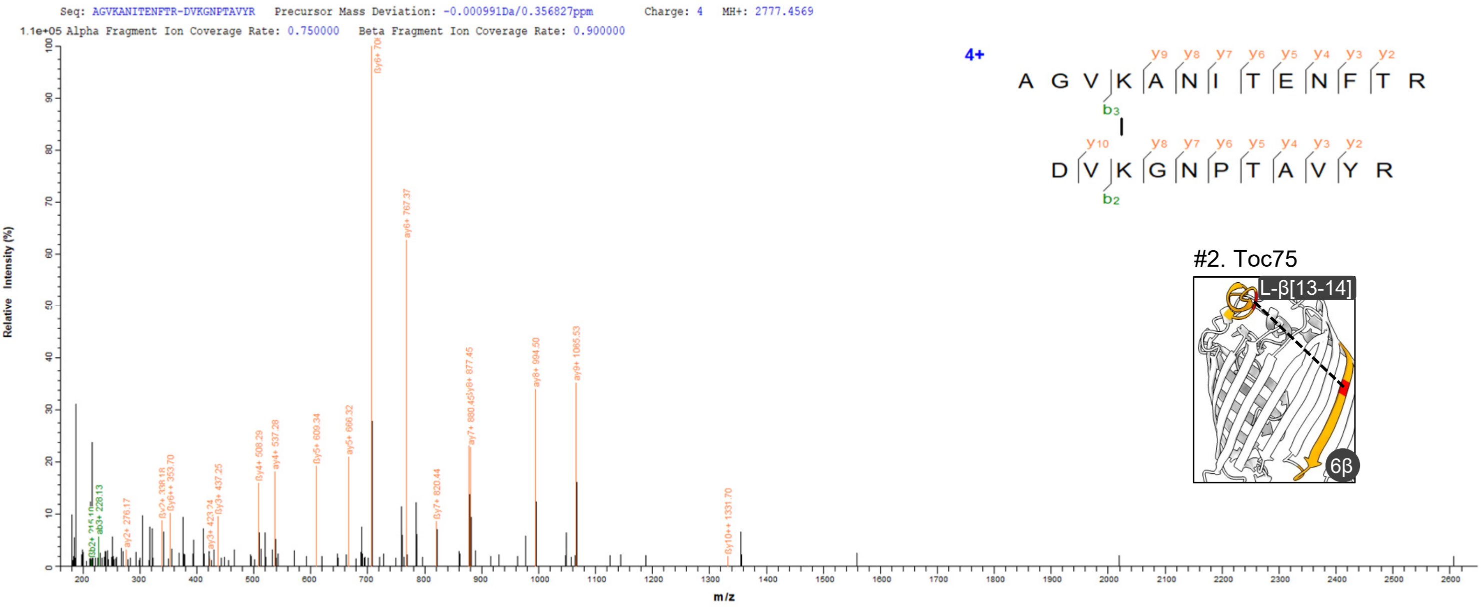

Supplementary Fig. 18 (part 2)

Toc75 (Crosslink #3)

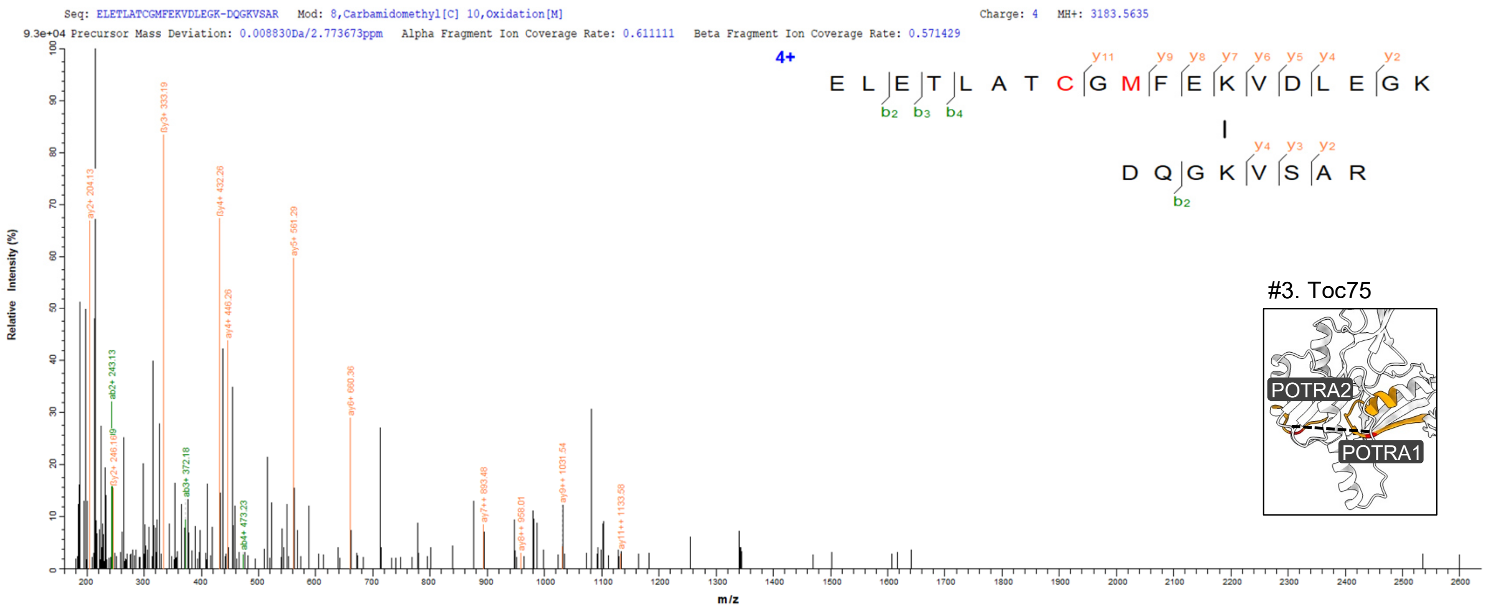

Toc75 (Crosslink #4)

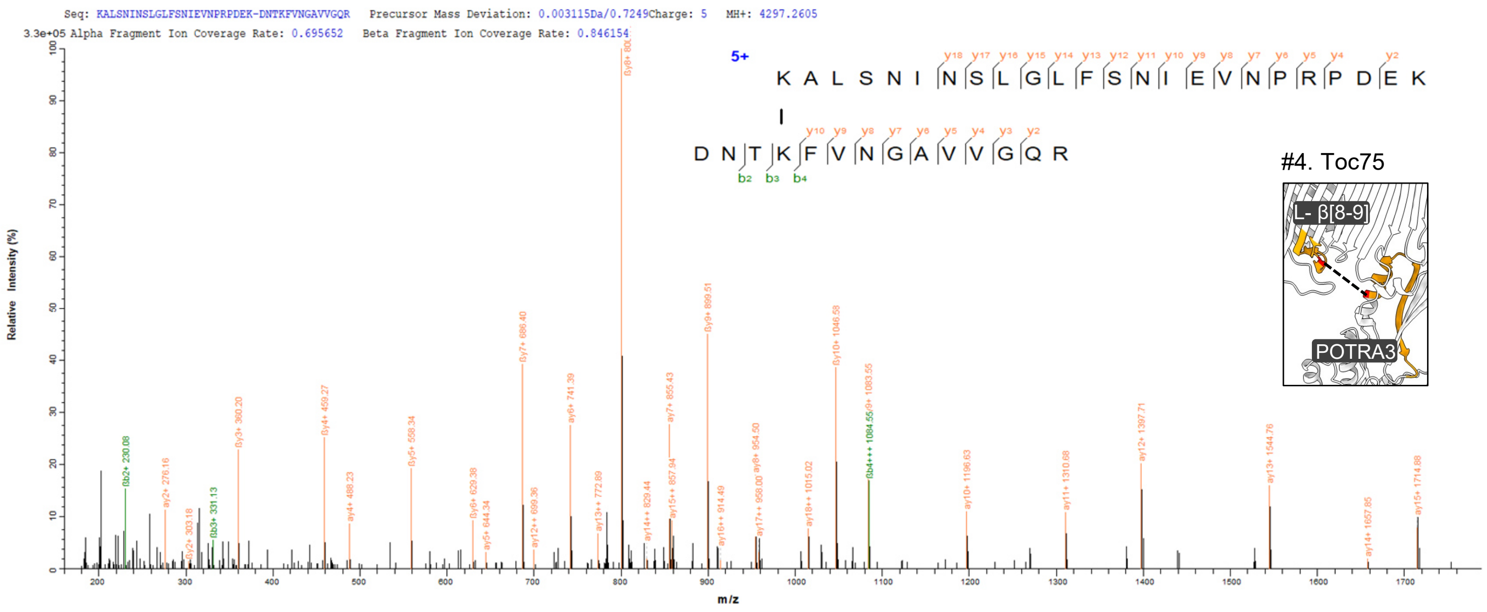

Supplementary Fig. 18 (part 3)

Toc75 (Crosslink #5)

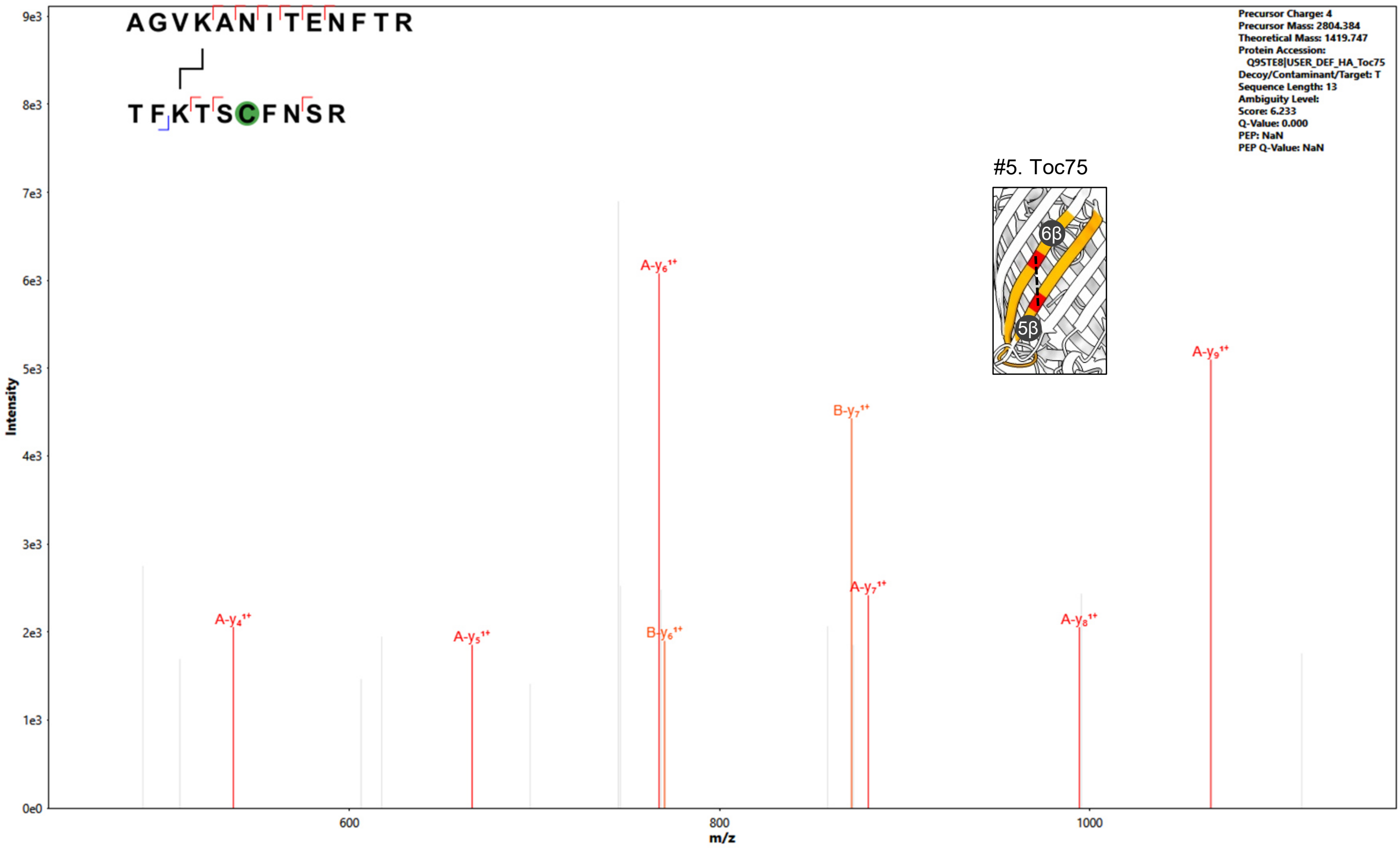

Supplementary Fig. 18 (part 4)

Toc75 (Crosslink #6)

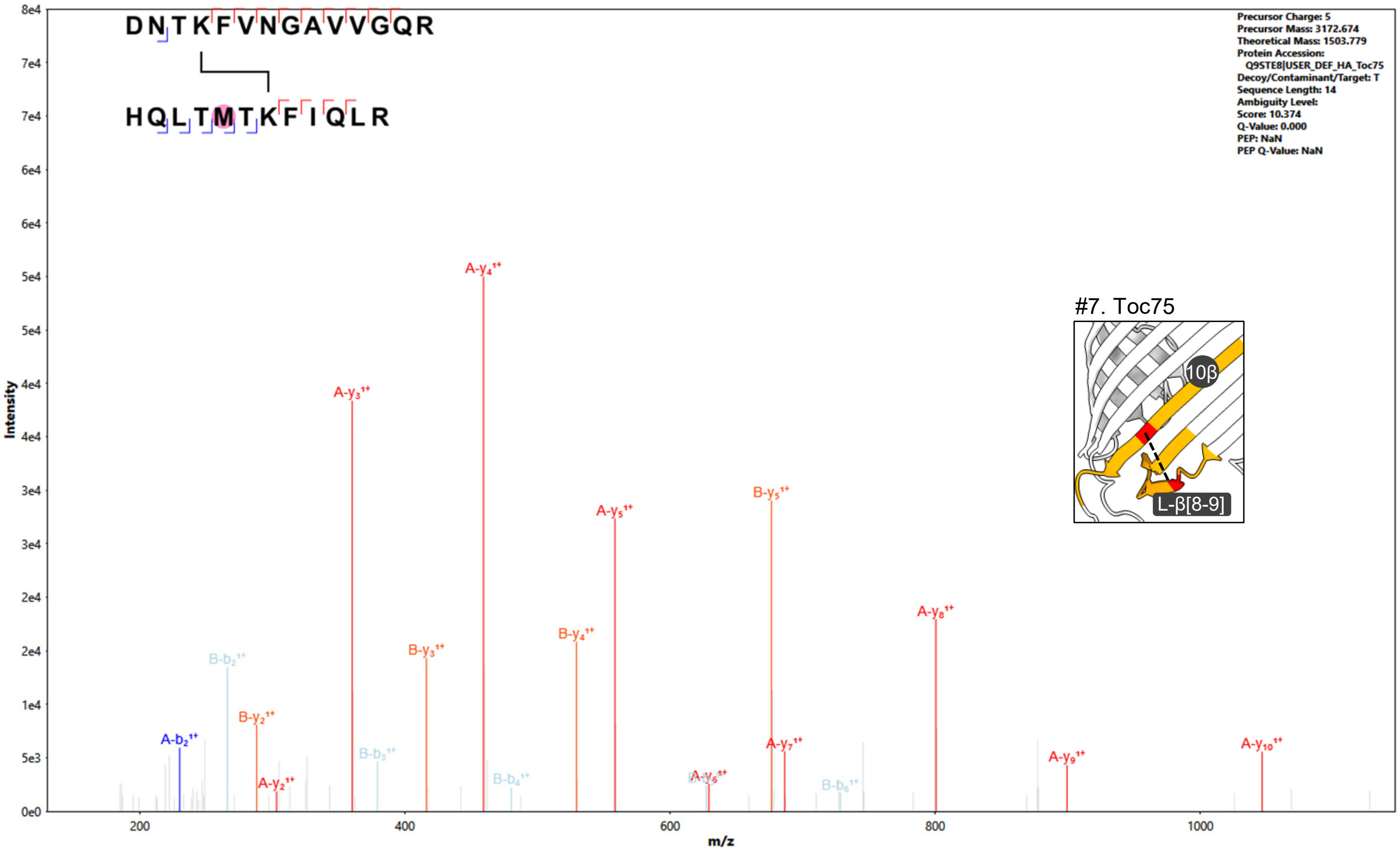

Supplementary Fig. 18 (part 5)

Toc75 (Crosslink #7)

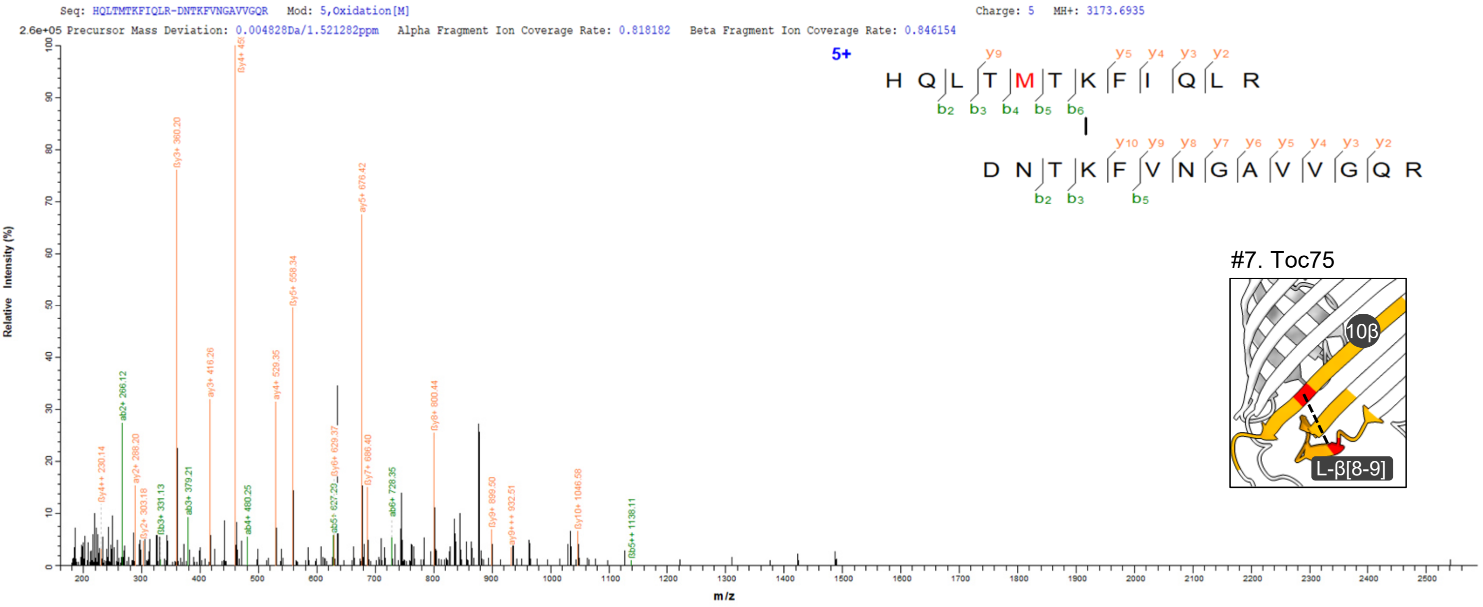

Toc75 (Crosslink #8)

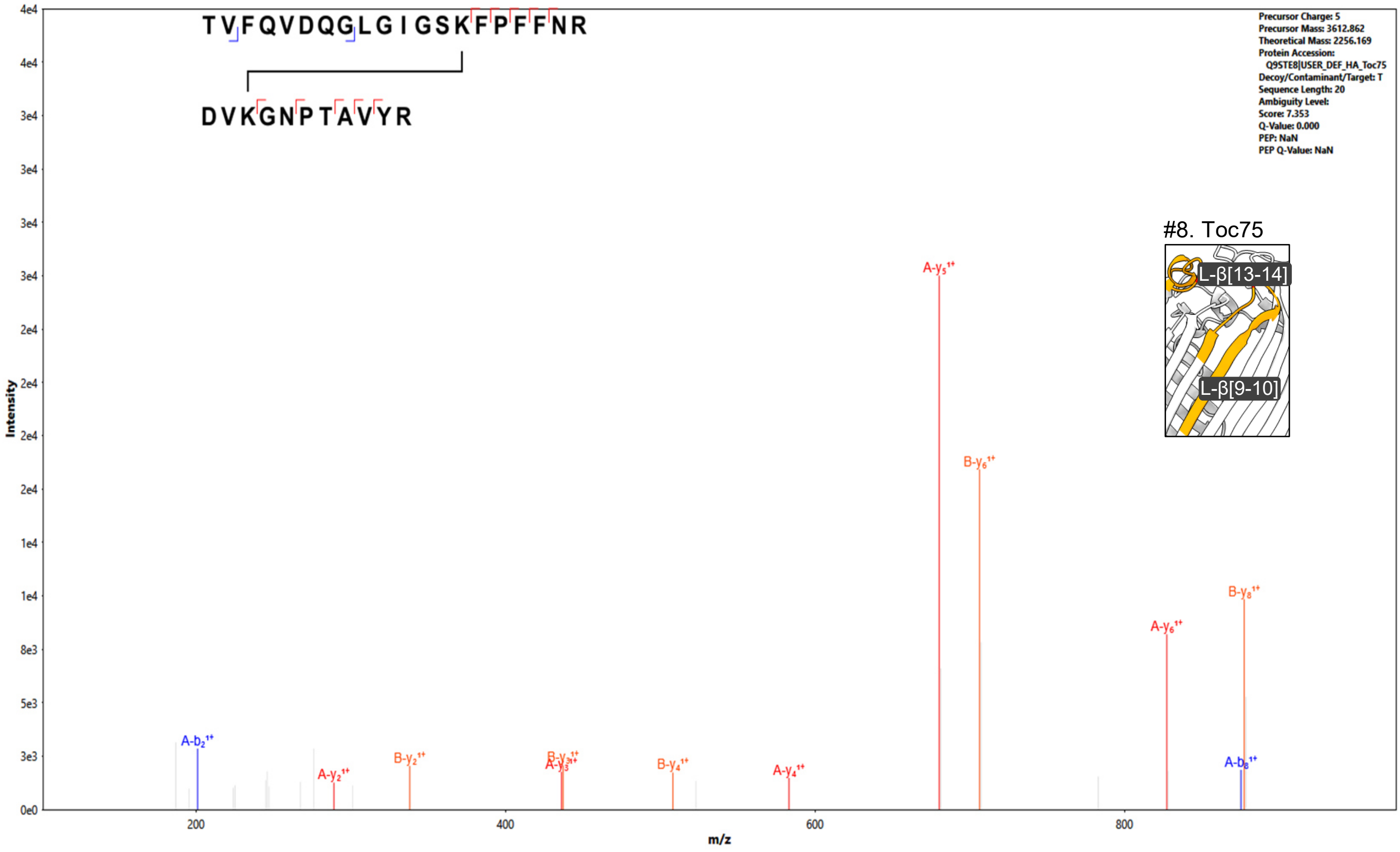

Supplementary Fig. 18 (part 6)

Toc75 (Crosslink #9)

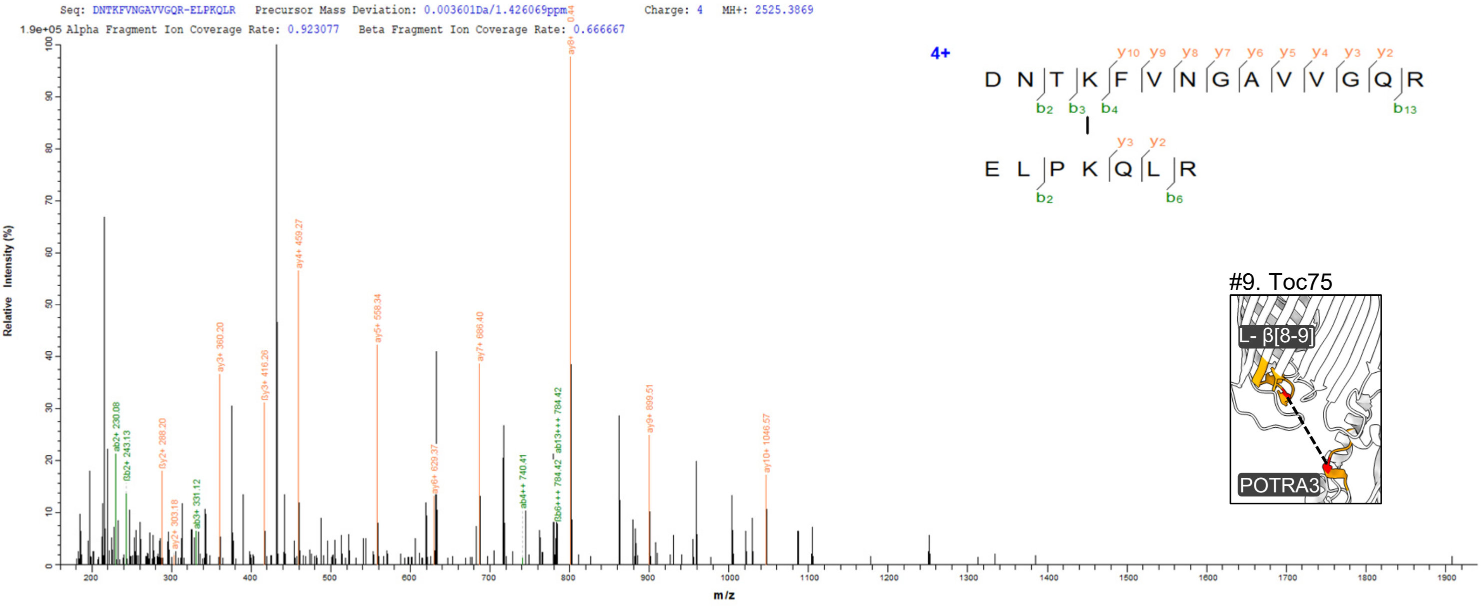

Toc75 (Crosslink #10)

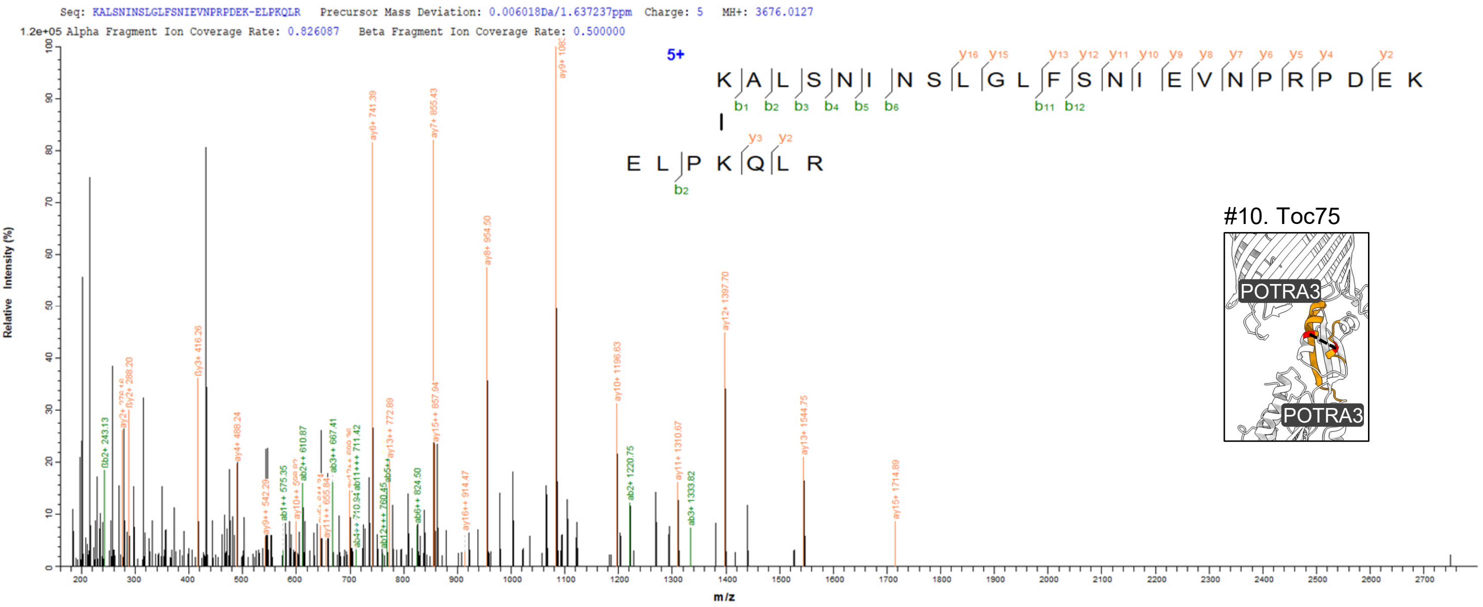

Supplementary Fig. 18 (part 7)

Toc75 (Crosslink #11)

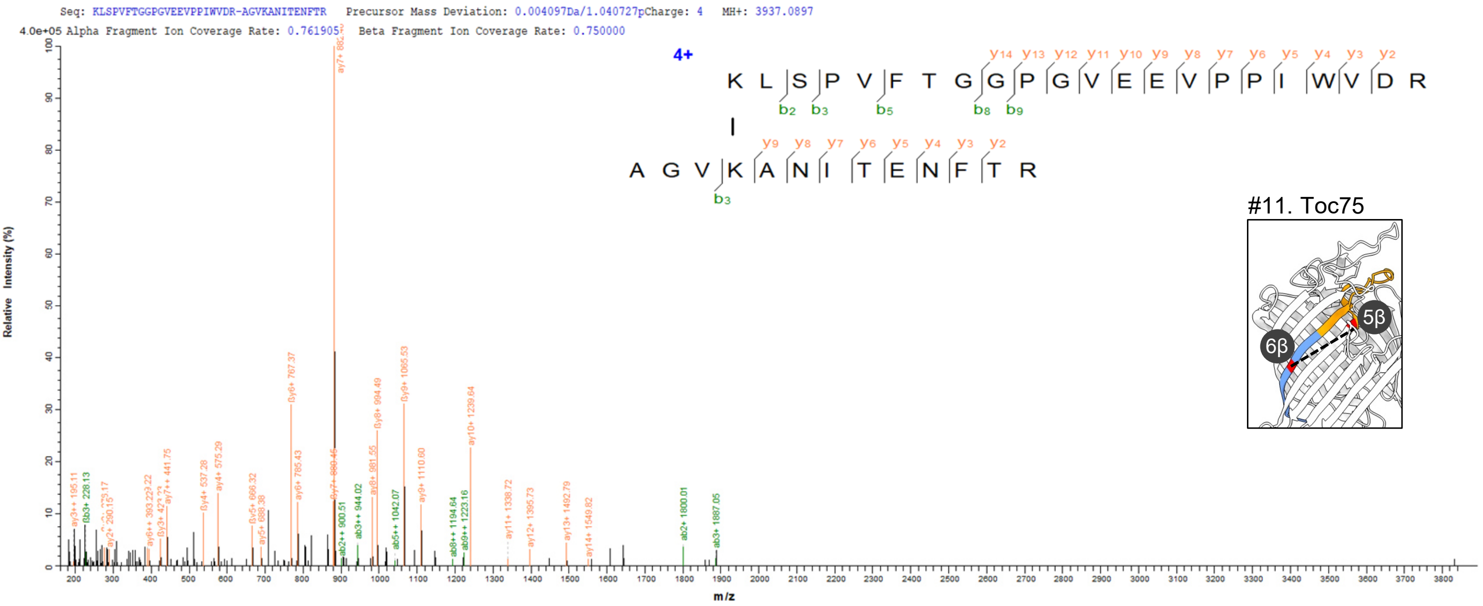

Toc75 (Crosslink #12)

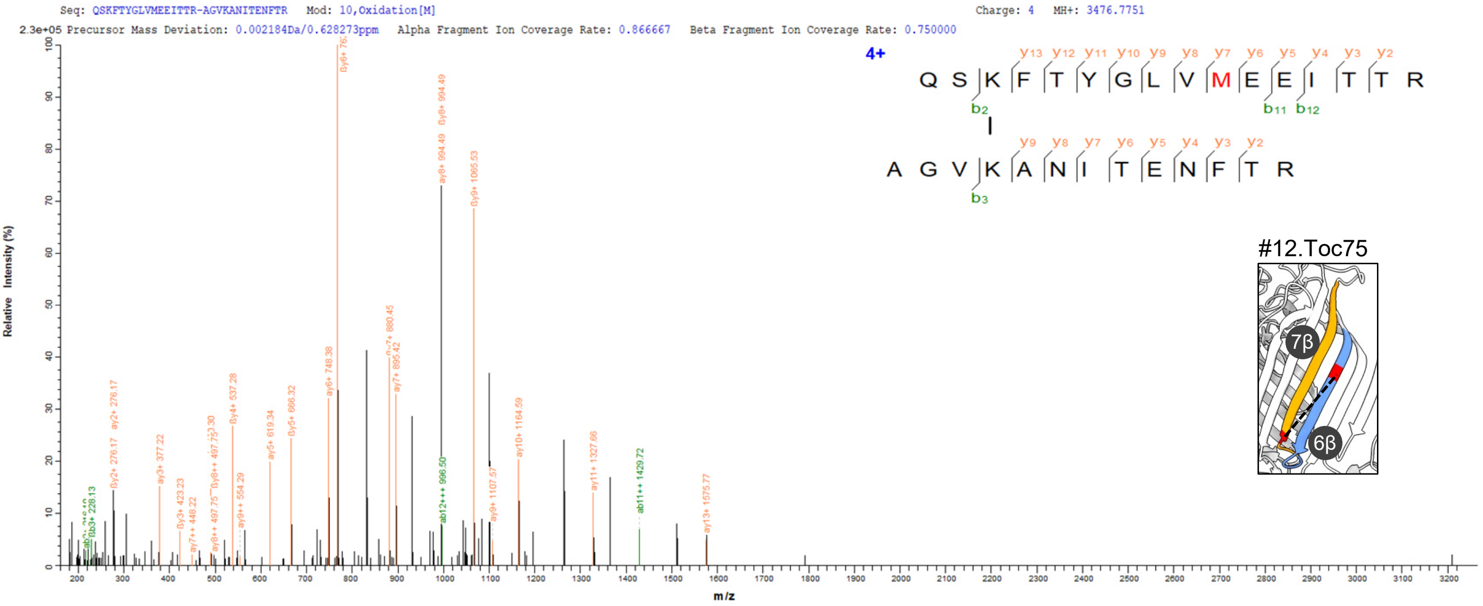

Supplementary Fig. 18 (part 8)

Toc75 (Crosslink #13)

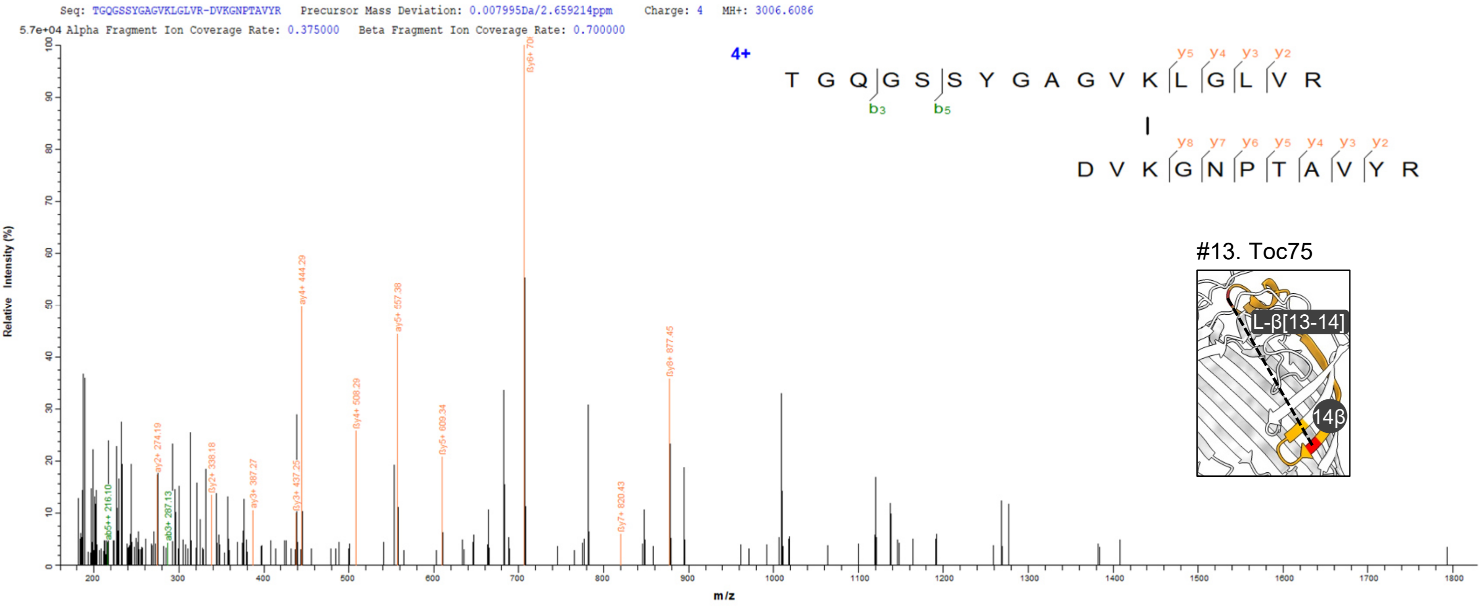

Toc159 (Crosslink #14)

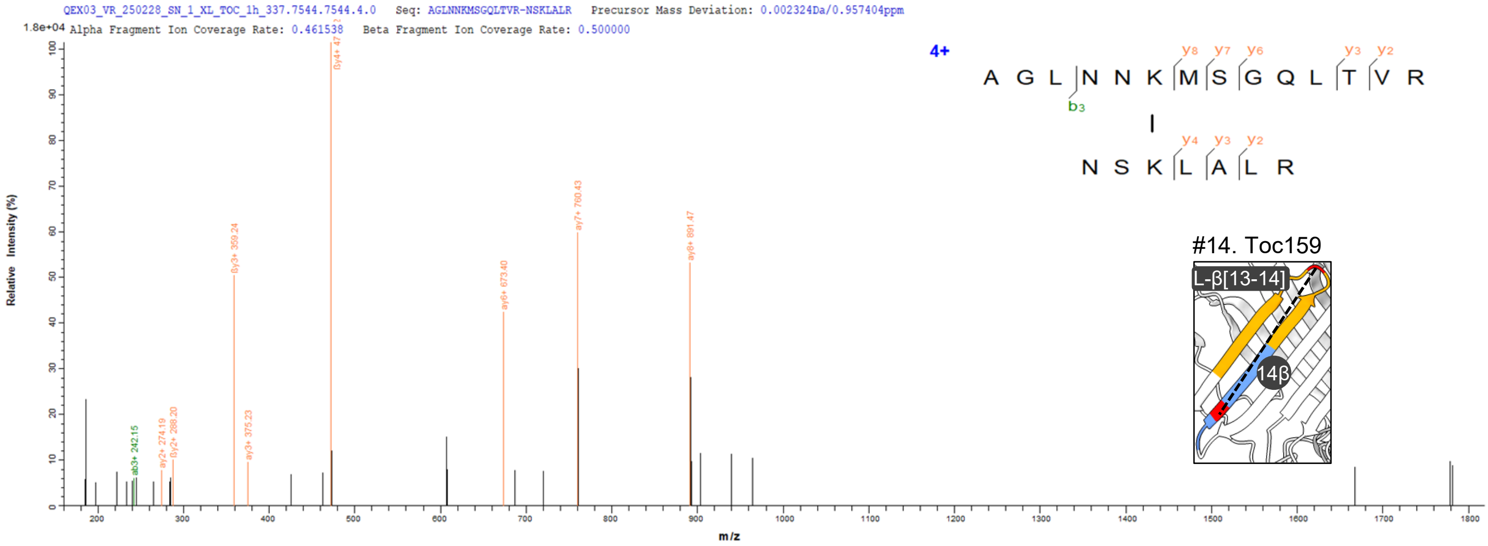

Supplementary Fig. 18 (part 9)

Toc159 (Crosslink #15)

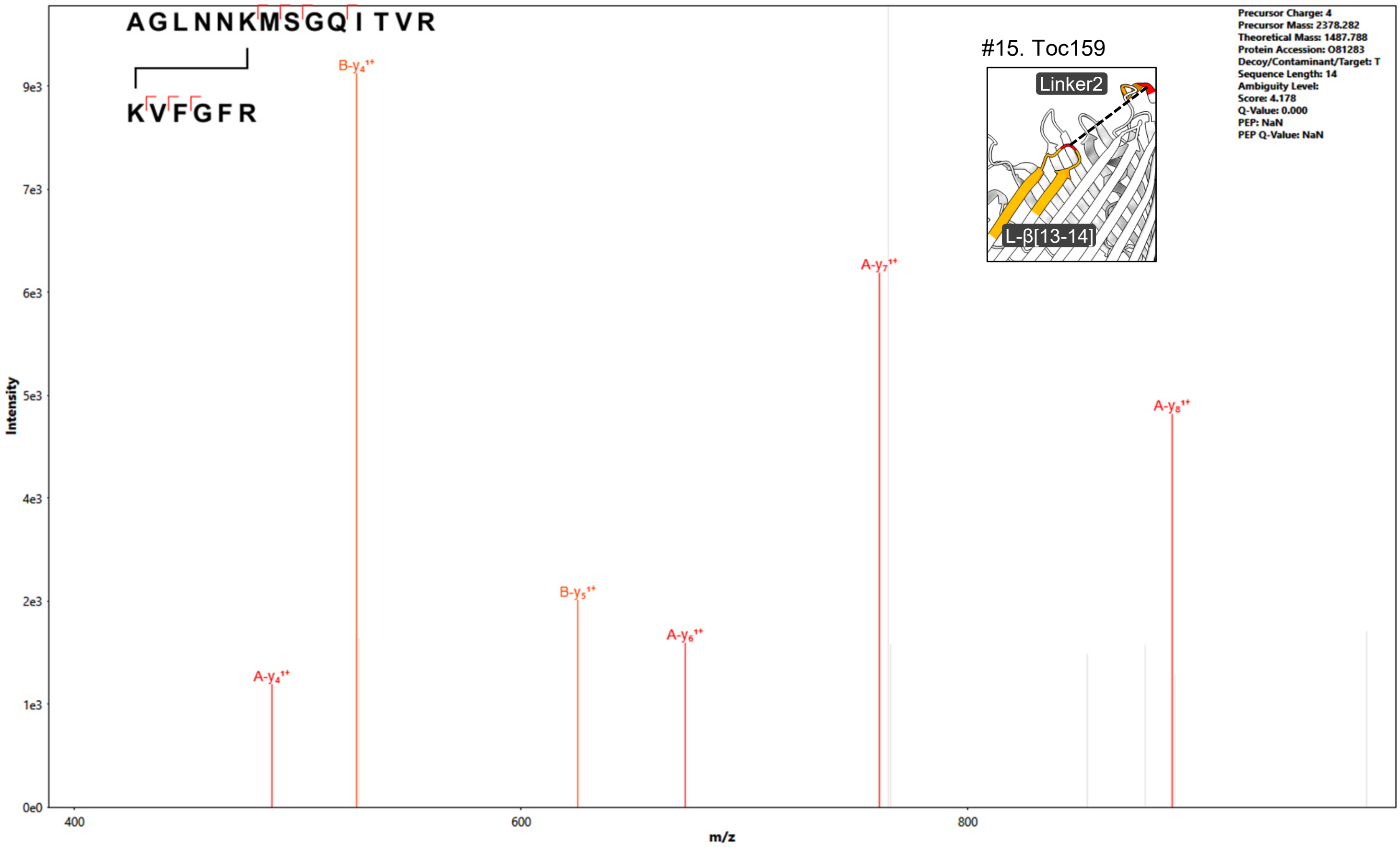

Toc159 (Crosslink #16)

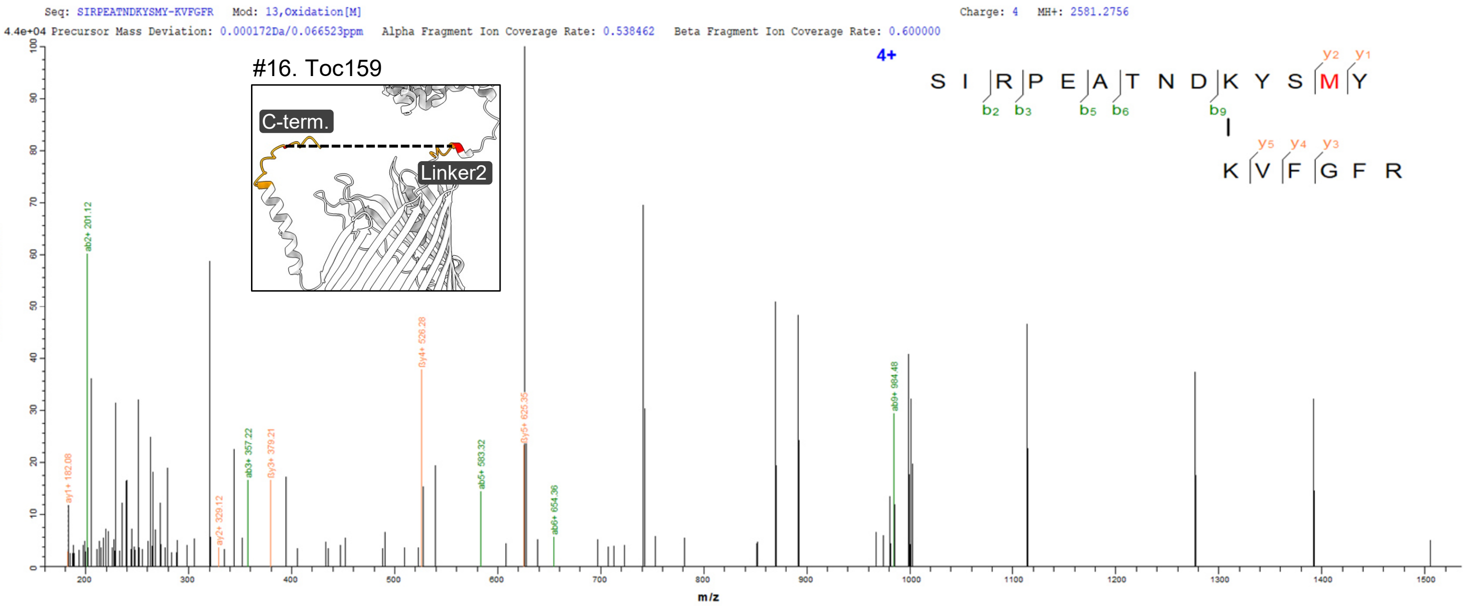

Supplementary Fig. 18 (part 10)

Toc159 (Crosslink #17)

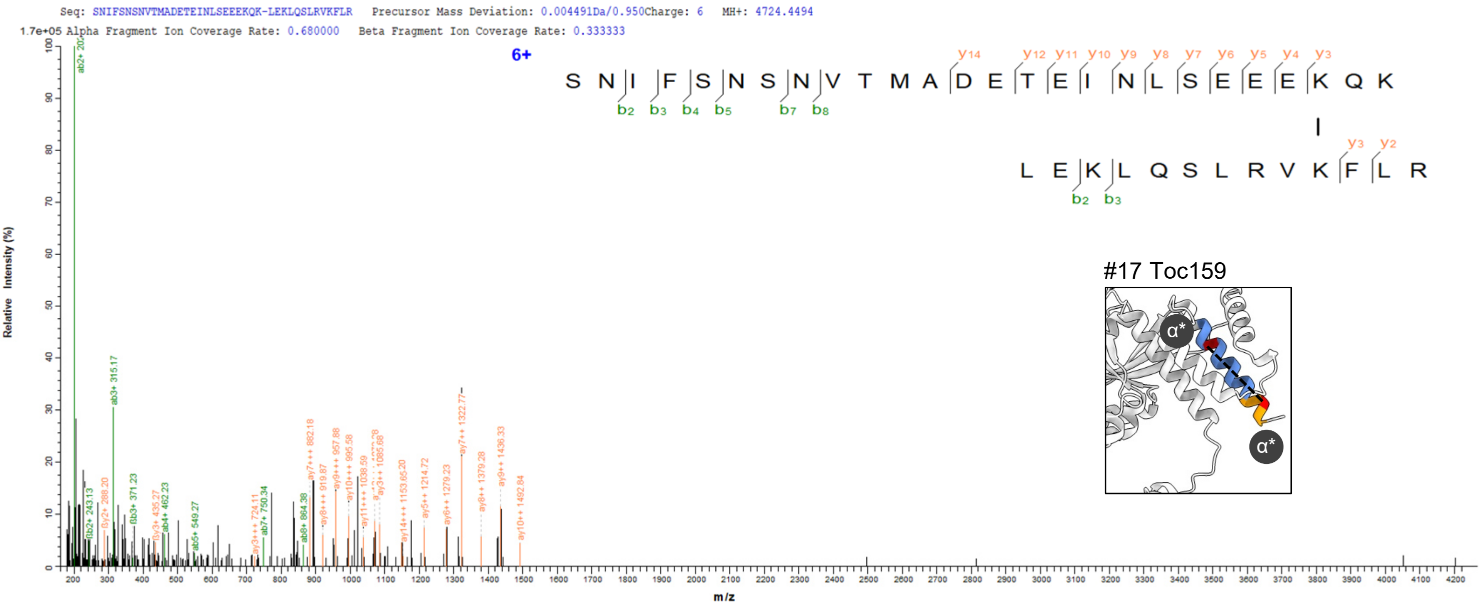

Toc159 (Crosslink #18)

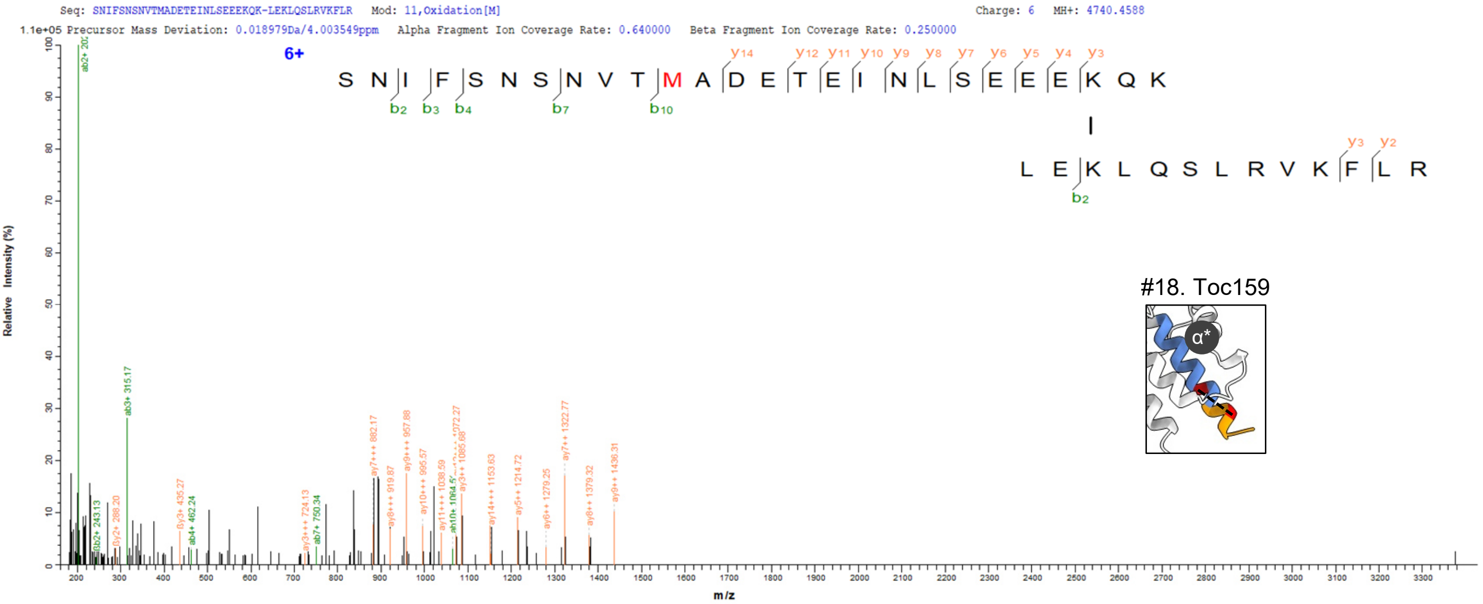

Toc159 (Crosslink #19)

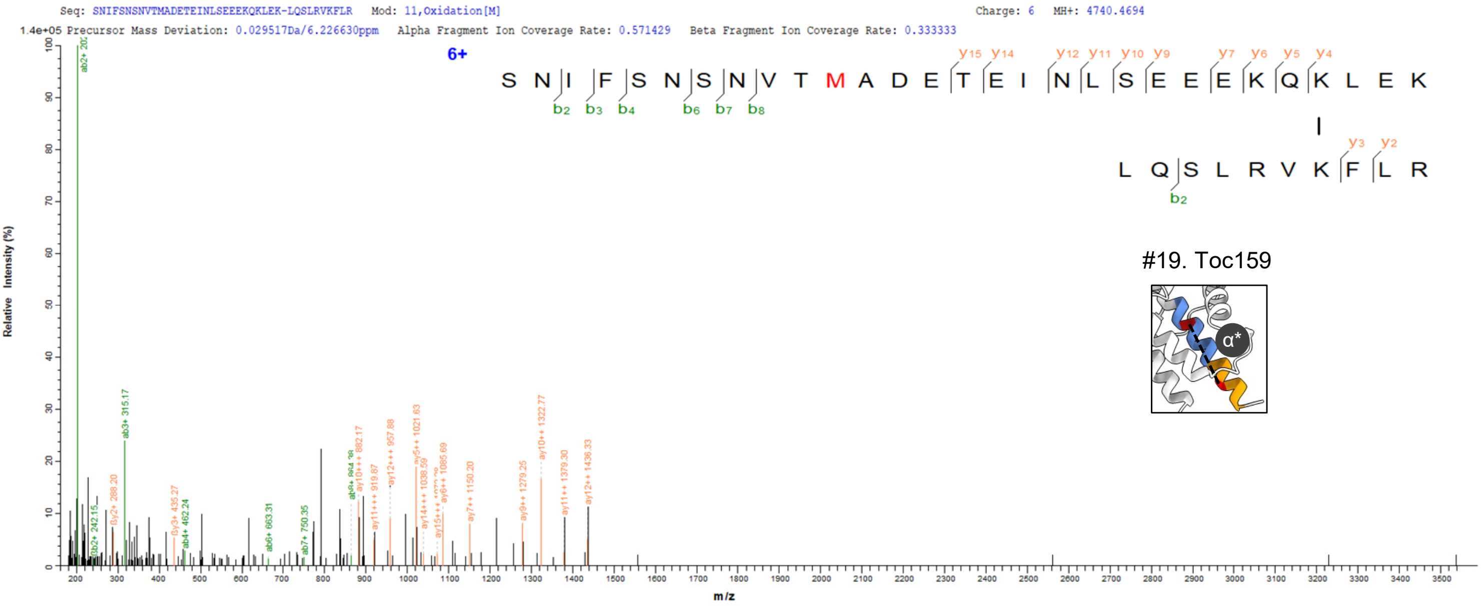

Supplementary Fig. 18 (part 11)

Toc159 (Crosslink #20)

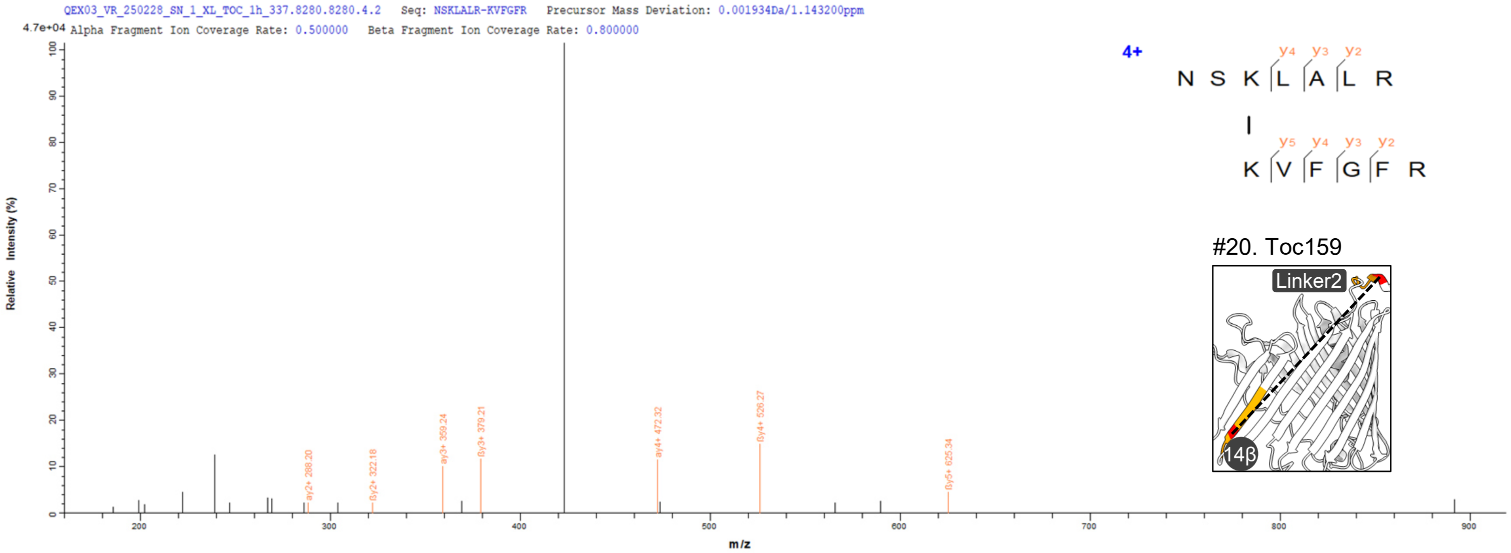

Toc33 (Crosslink #21)

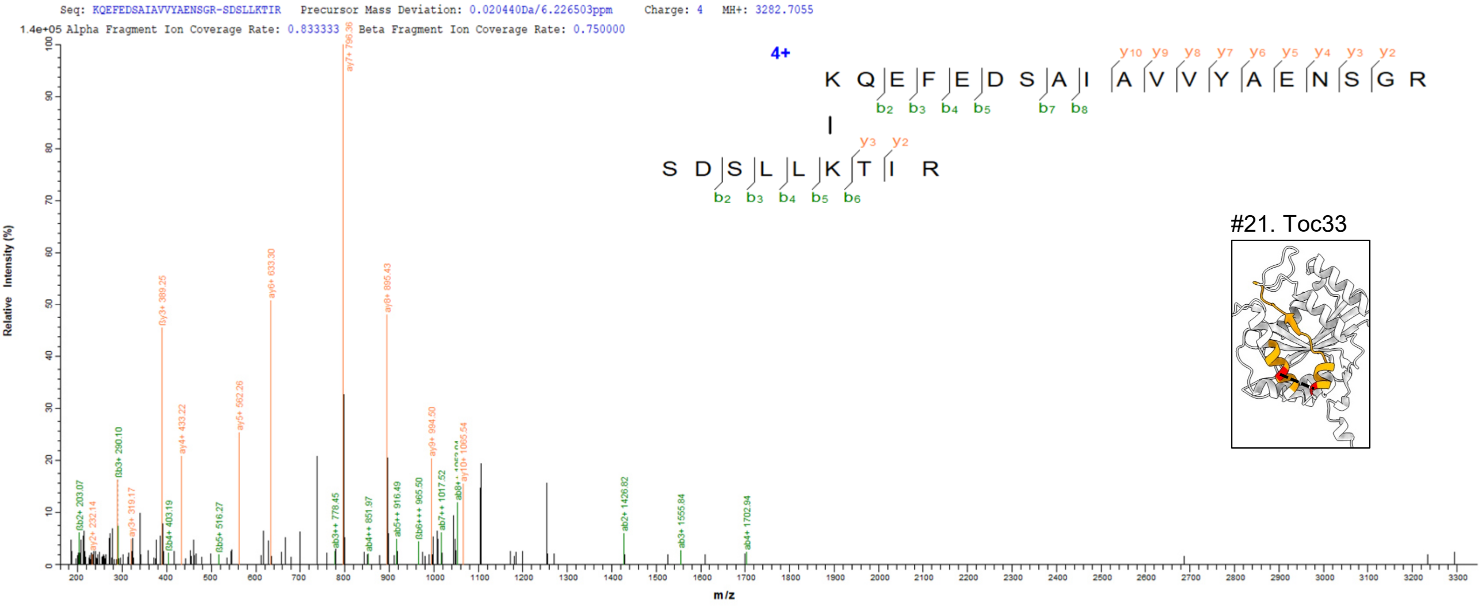

Supplement: Supplementary file 1 — Supplementary Information [file 41467_2026_71676_MOESM1_ESM.pdf]
