# Supplementary material for: Assembly of two functionally-distinct protein import complexes in the outer membrane of plant chloroplasts
Source: Nat Commun. 2026 Apr 20;17:5433. doi: 10.1038/s41467-026-71676-6 (PMC13279931; doi:10.1038/s41467-026-71676-6)
Supplement: Supplementary file 2 — Description of Additional Supplementary Files [file 41467_2026_71676_MOESM2_ESM.pdf]

Description of Additional Supplementary Files

File Name: Supplementary Data 1

Description: Mass spectrometry analysis of stained polypeptides and in-solution digestion of purified HA-Toc75 complexes.

File Name: Supplementary Data 2

Description: List of peptides including BS3 crosslinks identified by LC-MS/MS.

File Name: Supplementary Data 3

Description: Label-free quantitative mass-spectrometry analysis of affinity-purified HA-Toc75 samples.

File Name: Supplementary Data 4

Description: Primers used in this study.

a. Primers used to generate the *HA-Toc75* transgene.

b. Primers used for *TOC* gene expression analysis by RT-PCR.

c. Primers used to construct transit peptide fusions of *SSU* or *E1α* to *YFP/CFP* for protoplast expression.

d. Primers used for gene expression analysis by RT-PCR following protoplast expression.

e. Primers used to make the Toc33 GTPase domain construct for co-IP assays.

File Name: Supplementary Movie 1

Description: Front view from molecular dynamics simulation of the TOC-P complex.

File Name: Supplementary Movie 2

Description: Top-down view from molecular dynamics simulation of the TOC-P complex.
