# Supplementary material for: Assembly of two functionally-distinct protein import complexes in the outer membrane of plant chloroplasts
Source: Nat Commun. 2026 Apr 20;17:5433. doi: 10.1038/s41467-026-71676-6 (PMC13279931; doi:10.1038/s41467-026-71676-6)
Supplement: Supplementary file 9 — Reporting Summary [file 41467_2026_71676_MOESM9_ESM.pdf]

nature portfolio

Corresponding author(s): Paul Jarvis

Last updated by author(s): Mar 23, 2026

Reporting Summary

Nature Portfolio wishes to improve the reproducibility of the work that we publish. This form provides structure for consistency and transparency in reporting. For further information on Nature Portfolio policies, see our Editorial Policies and the Editorial Policy Checklist.

Statistics

For all statistical analyses, confirm that the following items are present in the figure legend, table legend, main text, or Methods section.

n/a	Confirmed	
☐	☒	The exact sample size (*n*) for each experimental group/condition, given as a discrete number and unit of measurement
☐	☒	A statement on whether measurements were taken from distinct samples or whether the same sample was measured repeatedly
☐	☒	The statistical test(s) used AND whether they are one- or two-sided<br>*Only common tests should be described solely by name; describe more complex techniques in the Methods section.*
☒	☐	A description of all covariates tested
☒	☐	A description of any assumptions or corrections, such as tests of normality and adjustment for multiple comparisons
☐	☒	A full description of the statistical parameters including central tendency (e.g. means) or other basic estimates (e.g. regression coefficient) AND variation (e.g. standard deviation) or associated estimates of uncertainty (e.g. confidence intervals)
☐	☒	For null hypothesis testing, the test statistic (e.g. *F*, *t*, *r*) with confidence intervals, effect sizes, degrees of freedom and *P* value noted<br>*Give P values as exact values whenever suitable.*
☒	☐	For Bayesian analysis, information on the choice of priors and Markov chain Monte Carlo settings
☒	☐	For hierarchical and complex designs, identification of the appropriate level for tests and full reporting of outcomes
☒	☐	Estimates of effect sizes (e.g. Cohen's *d*, Pearson's *r*), indicating how they were calculated

Our web collection on statistics for biologists contains articles on many of the points above.

Software and code

Policy information about availability of computer code

Data collection	Immunoblots were collected using an ImageQuant LAS-4000 imager. The protein bands (staining/radiolabelling) were quantified using NIH ImageJ and the raw data were exported to Microsoft Excel for analysis.<br><br>Data on radiolabelled polypeptides were collected using a Storm Molecular Imager, Molecular Dynamics.<br><br>Chlorophyll levels of mature plants were measured using a SPAD-502 meter (Konica Minolta).<br><br>Protein sequences for sequence alignment were obtained from a variety of sources including Phytozome and Uniprot.<br><br>The 3D structures of complexes were predicted by AlphaFold3 and AlphaLink2. The top-scoring models were studied using Chimera-1.17.<br><br>Tandem mass (MS/MS) spectra were analysed using Proteome Discoverer, pLink3, and MetaMorpheus 1.0.9.<br><br>Electron micrographs were captured using a 200 kV electron microscope (JEM-2100Plus). The EM raw images were processed in CryoSPARC, where CTFFIND4 was used for Contrast Transfer Function (CTF) estimation.<br><br>The coarse-grained molecular dynamics (MD) simulations were run in GROMACS v2021.4.
Data analysis	Statistical analyses (mean, standard error of the mean, and Student's t-test) were conducted using Microsoft Excel.

Image analysis was conducted using NIH ImageJ.

For MD simulations, ad hoc Python 3 scripts (containing MD analysis and NumPy libraries) were used during post-processing and data analysis.

Multiple sequence alignments were performed using Clustal Omega.

For manuscripts utilizing custom algorithms or software that are central to the research but not yet described in published literature, software must be made available to editors and reviewers. We strongly encourage code deposition in a community repository (e.g. GitHub). See the Nature Portfolio guidelines for submitting code & software for further information.

Data

Policy information about availability of data

All manuscripts must include a data availability statement. This statement should provide the following information, where applicable:

- Accession codes, unique identifiers, or web links for publicly available datasets
- A description of any restrictions on data availability
- For clinical datasets or third party data, please ensure that the statement adheres to our policy

Sequences were obtained from the TAIR (http://www.arabidopsis.org/), Phytozome (https://phytozome.jgi.doe.gov/pz/portal.html), Uniprot (https://www.uniprot.org/), or National Center for Biotechnology Information (NCBI) (https://www.ncbi.nlm.nih.gov/) databases.

The following Data availability statement is included in the manuscript: "All data generated or analysed during this study are included in this published article or its supplementary information, apart from the following. The mass spectrometry proteomics data have been deposited to the ProteomeXchange Consortium via the PRIDE partner repository94; specifically, the HA-Toc75 proteomic data (experiments i, ii and v in the Methods) and the crosslinking mass spectrometry data (experiments iii and iv in the Methods) have been deposited under accession codes PXD072672 and PXD064453, respectively (https://www.ebi.ac.uk/pride/archive/projects/). Coordinates and molecular dynamics parameters from the molecular dynamics simulations have been deposited in the Zenodo repository under the accession code 18467186 (https://zenodo.org/records/18467186). All unique materials are readily available from the authors. Source data are provided with this paper."

Research involving human participants, their data, or biological material

Policy information about studies with human participants or human data. See also policy information about sex, gender (identity/presentation), and sexual orientation and race, ethnicity and racism.

Reporting on sex and gender	N/A
Reporting on race, ethnicity, or other socially relevant groupings	N/A
Population characteristics	N/A
Recruitment	N/A
Ethics oversight	N/A

Note that full information on the approval of the study protocol must also be provided in the manuscript.

Field-specific reporting

Please select the one below that is the best fit for your research. If you are not sure, read the appropriate sections before making your selection.

[x] Life sciences [ ] Behavioural & social sciences [ ] Ecological, evolutionary & environmental sciences

For a reference copy of the document with all sections, see nature.com/documents/nr-reporting-summary-flat.pdf

Life sciences study design

All studies must disclose on these points even when the disclosure is negative.

Sample size	No statistical measures were used to predetermine sample size. The sample sizes were determined based on those used in previous, similar reports in the literature. Unless specifically noted otherwise in the figure legends or Methods, we performed at least three biological replicates for each experiment, which is typical for this type of experimental analysis and usually sufficient for reliable conclusions to be drawn. The phenotypes of Arabidopsis plants were highly consistent, with minimal variation.
Data exclusions	No data were excluded from the analyses.
Replication	For physiological, molecular, biochemical, and structural experiments, plants of the different genotypes were randomly selected to mitigate potential variation due to environmental factors. Samples were allocated into experimental groups dependent on the genotype.
Randomization	For physiological, molecular and cell biological experiments, plants of the different genotypes were randomly selected to mitigate potential variation due to environmental factors. Samples were allocated into experimental groups dependent on the genotype.

Blinding	For physiological experiments, plants were selected randomly without consideration of genotype until the point of data analysis, and similar results were obtained with independent samples. For molecular and cell biological experiments, blinding was not required because the results of such measurements are directly obtained through software, and thus are not affected by knowledge of sample identities.

Reporting for specific materials, systems and methods

We require information from authors about some types of materials, experimental systems and methods used in many studies. Here, indicate whether each material, system or method listed is relevant to your study. If you are not sure if a list item applies to your research, read the appropriate section before selecting a response.

Materials & experimental systems

n/a	Involved in the study
☐	☒ Antibodies
☒	☐ Eukaryotic cell lines
☒	☐ Palaeontology and archaeology
☒	☐ Animals and other organisms
☒	☐ Clinical data
☒	☐ Dual use research of concern
☐	☒ Plants

Methods

n/a	Involved in the study
☒	☐ ChIP-seq
☒	☐ Flow cytometry
☒	☐ MRI-based neuroimaging

Antibodies

Antibodies used

The following antibodies were used in this study:

Commercial antibodies:
anti-actin (AS132640, Agrisera)
anti-histone H3 (ab1791, Abcam)
anti-HA (H6908, Sigma)
anti-atToc34 (AS07 238, Agrisera)
anti-rabbit IgG (12-348, Sigma)

Homemade/custom-made antibodies:
anti-atToc75-III POTRA-domain
anti-atToc159 A-domain
anti-atToc33 peptide
anti-atToc132 A-domain
anti-atToc120 A-domain
anti-atTic110 stromal domain
anti-atTic40 stromal domain
anti-atTic56
anti-atTic100
anti-atTic214

Validation

All antibodies were validated by immunoblotting in previously published work, and relevant citations have been provided.

Commercial antibodies were validated by the manufacturers as indicated on their web sites:

Rabbit anti-Toc34 was validated in Arabidopsis for immunoblotting by the manufacturer (https://www.agrisera.com/en/artiklar/toc34-arabidopsis-thliana.html).

Rabbit anti-actin antibody was validated in Arabidopsis for immunoblotting by the manufacturer (https://www.agrisera.com/en/artiklar/act-actin.html).

Rabbit anti-histone H3 antibody was validated in Arabidopsis for immunoblotting by the manufacturer (https://www.abcam.com/histone-h3-antibody-nuclear-marker-and-chip-grade-ab1791.html).

Rabbit anti-HA was validated for immunoblotting by the manufacturer (https://www.sigmaaldrich.com/GB/en/product/sigma/h6908).

Goat Anti-Rabbit IgG Antibody, HRP-conjugate was validated for immunoblotting by the manufacturer (https://www.sigmaaldrich.com/GB/en/product/mm/12348).

Dual use research of concern

Policy information about dual use research of concern

Hazards

Could the accidental, deliberate or reckless misuse of agents or technologies generated in the work, or the application of information presented in the manuscript, pose a threat to:

No	Yes	
x		Public health
x		National security
x		Crops and/or livestock
x		Ecosystems
x		Any other significant area

Experiments of concern

Does the work involve any of these experiments of concern:

No	Yes	
x		Demonstrate how to render a vaccine ineffective
x		Confer resistance to therapeutically useful antibiotics or antiviral agents
x		Enhance the virulence of a pathogen or render a nonpathogen virulent
x		Increase transmissibility of a pathogen
x		Alter the host range of a pathogen
x		Enable evasion of diagnostic/detection modalities
x		Enable the weaponization of a biological agent or toxin
x		Any other potentially harmful combination of experiments and agents

Plants

Seed stocks	All seed stocks were available in the laboratory or were obtained as gifts from other laboratories.
Novel plant genotypes	The HA-Toc75 transgenic lines were generated by Agrobacterium-mediated transformation of Arabidopsis thaliana using the floral dip method.
Authentication	Genotypes were authenticated by PCR and sequencing as appropriate.
