# Supplementary material for: Assembly of two functionally-distinct protein import complexes in the outer membrane of plant chloroplasts
Source: Nat Commun. 2026 Apr 20;17:5433. doi: 10.1038/s41467-026-71676-6 (PMC13279931; doi:10.1038/s41467-026-71676-6)
Supplement: Supplementary file 10 — Transparent Peer Review file [file 41467_2026_71676_MOESM10_ESM.pdf]

nature portfolio

Peer Review File

Assembly of two functionally-distinct protein import complexes in the outer membrane of plant chloroplasts

Corresponding Author: Professor R. Jarvis

This file contains all reviewer reports in order by version, followed by all author rebuttals in order by version.

Version 0:

Reviewer comments:

Reviewer #1

(Remarks to the Author)
In this manuscript, the authors examined and compared the protein abundance of various TOC and TIC components across different tissues, in various toc mutants, and in seedlings undergoing the de-etiolation process. They also created an HA-Toc75 transgene to rescue the pale-green kd-toc75 (mar1) mutant and used HA-Toc75 plants for affinity purification and in vivo labeling to investigate the sequential assembly of the TOC-P complex. By combining these experiments with structural predictions of TOC complexes using AlphaFold3 (AF3), molecular dynamics (MD) simulation analyses, and in vivo import assays, the authors conclude that they have revealed the presence of two distinct TOC complexes—TOC-P and TOC-N—with unique structural organizations and functions. They also propose that Toc75 serves as the central hub and driver in TOC complex biogenesis.

The manuscript presents substantial data supporting the existence of the TOC-P complex. The use of AF3 and MD simulations suggests the presence and importance of two linker regions in TOC structural dynamics. Observed changes in protein abundance allow reasonable inference for Toc75 functioning as the primary driver of TOC assembly. These results offer further insights into the existence and functions of multiple TOC complexes. However, the concept of distinct TOC complexes—one associated with photosynthetic substrates and another with non-photosynthetic or housekeeping substrates, each preferentially distributed in photosynthetic and non-photosynthetic tissues—has been previously demonstrated by multiple groups, including the authors' own earlier work. Notably, Ivanova et al. (MBC 15, 3379–3392, 2004) used immunoprecipitation to show that Toc159 preferentially forms complexes with Toc33 and Toc75, while Toc132 preferentially associates with Toc34 and Toc75. Additionally, a model for stepwise TOC complex assembly (Toc75 followed by Toc33, then Toc159) has already been proposed and partially validated. Several parts of the manuscript also require more detailed explanation, and some interpretations of the data appear overstated and should be revised. Detailed comments follow below.

Major Comments:
1. Using Fig. 1e and Supplementary Fig. 1, the authors conclude (line 88):
"Receptor isoforms of Toc33 and Toc159 were enriched in leaves, whereas Toc34 and Toc132/120 … were enriched in roots and petals. All TOC components were present at relatively low levels in etiolated seedlings …, revealing clear differentiation of TOC composition in different plastid types."

To support these conclusions, the authors should specify the total protein amount loaded per lane, include internal loading controls (e.g., housekeeping proteins), and provide quantitative analyses of the immunoblot data. For example, in Fig. 1e, the immunoblot signal intensities for Toc159 in the 50% and 100% protein loadings of etiolated (Et) samples appear nearly identical. In Supplementary Fig. 1, the Toc159 signal in leaf (Lf) samples is also comparable regardless of protein loading. In Supplementary Fig. 1, the "Rubisco" signal for the 50% Lf sample is stronger than the signal of the 100% sample, which raises concerns about linearity and normalization. Furthermore, in Supplementary Fig. 1, the Toc34 signal is indeed stronger in petals than in leaves, but since twice the amount of petal proteins was loaded, enrichment in petals cannot be concluded without normalization.

2. Regarding Fig. 1f (HA-Toc75 purification), the authors state (line 96): "The analysis showed that Toc75 is mainly associated with Toc33 and Toc159 in leaf chloroplasts, and to a lesser extent with Toc34, Toc132, and Toc120. In contrast, Toc75 is mainly associated with Toc34, Toc132, and Toc120 in root leucoplasts, and to a lesser extent with Toc33 and

Toc159." This conclusion is further illustrated schematically in Fig. 1g.

(1) The data in Fig. 1f only support the conclusion that TOC-P is more abundant in leaves than roots, and TOC-N is more abundant in roots than leaves. The data cannot be used to compare the relative abundance of TOC-P versus TOC-N within the same tissue, as immunoblot signals for different proteins are not directly comparable. Variability in antibody affinities, epitope accessibility, protein transfer efficiency, and chemiluminescence detection time must be considered. To support the conclusions in Fig. 1g, additional experimental controls are needed—for instance, quantification by comparing to known amounts of recombinant proteins to allow accurate comparison between different proteins.

(2) The data also do not rule out the possibility of a Toc34–Toc75–Toc159 complex, nor do they confirm the proposed Toc75–Toc34–Toc132 (TOC-N) complex. To validate the presence of the TOC-N complex, immunoprecipitation using Toc132/Toc120 and Toc34 as bait proteins should be conducted.

3. In Fig. 2, the authors conclude that: "Toc75 and Toc33/34 are key players in stabilizing the integrity of the TOC-P and TOC-N complexes," based on immunoblot signals of TOC components in toc mutants. However, the current data primarily demonstrate that kd-toc75, ko-toc33, and ko-toc34 mutations reduce the abundance of individual TOC components, not the integrity of TOC complexes. To support conclusions about complex integrity, co-immunoprecipitation assays should be performed to test whether Toc75 still interacts with Toc33, Toc34, Toc159, and Toc132 in these mutant backgrounds.

4. The authors describe purification of the TOC-P complex from HA-Toc75 plants for use in negative-stain EM analysis (Fig. 3e and Extended Data Fig. 1).

(1) However, as shown in Fig. 1f and Supplementary Table 1, both TOC-P and TOC-N components are present in the purified sample. It is unclear how the authors selectively purified only the TOC-P complex for structural analysis. This point requires clarification.

(2) Additionally, scale bars are missing in the EM images (Extended Data Fig. 1 and Fig. 3e). Their inclusion is necessary for interpretation.

5. Referring to Extended Data Fig. 7, the authors state (line 252): "The missing TMH is needed to properly anchor and stabilize the Toc159 β-barrel in the membrane." In the figure legend, they add: "The mutant protein was extracted much more readily than the WT protein, indicating instability."

To strengthen this conclusion, the authors should provide immunoblots of the supernatant fraction following alkaline extraction, along with appropriate internal controls. These should include markers for soluble, peripheral, and membrane-associated proteins to validate extraction efficiency and normalize protein loading. Ponceau S staining has limited sensitivity; in particular, LHC staining is not visible in WT samples, making it unreliable for normalization.

6. AF3 uses structural data from known PDB entries—including the Chlamydomonas TOC-TIC supercomplex and Toc34/Toc33 dimers—to predict the Toc33–Toc75–Toc159 assembly. The authors state (line 263): "Comparing the different structures, we found that TOC-P from A. thaliana resembles the cryo-EM and AF3-predicted structures of the algal TOC complex, indicating that structural organization of the TOC complex is broadly conserved in plants and green algae."

This raises concerns about circular reasoning, as the referenced structures were incorporated into the model generation itself. The authors should clarify how this potential bias was controlled or, alternatively, revise the statement to acknowledge the limitation.

7. Using Extended Data Fig. 8, the authors state (line 267): "However, affinity-purified TOC-P complexes from our HA-Toc75 lines, as well as from TAP-tagged transgenic lines (TAP-Toc159, TAP-Toc33), showed negligible interaction with inner membrane TIC components (Extended Data Fig. 8). This revealed a clear and remarkable distinction from the algal TOC apparatus, which is tightly integrated with the TIC apparatus in an interlocked TOC-TIC supercomplex. This implies that the plant and algal protein import systems are substantially different."

(1) While this observation is intriguing, it is important to consider the effects of different detergent conditions on the integrity and composition of membrane protein complexes. In this study, 1% DDM was used to solubilize chloroplasts, whereas previous studies on the algal TOC-TIC supercomplex (e.g., doi.org/10.1038/s41586-023-05744-y and doi.org/10.1016/j.cell.2022.10.030) employed milder conditions, such as 1% LMNG supplemented with 0.2% digitonin or 0.12% CHS. These gentler detergents are known to better preserve labile or transient interactions between membrane complexes. Moreover, the current study used TOC components (HA-Toc75, TAP-Toc159, TAP-Toc33) as purification baits, whereas the algal complex was purified using a tagged TIC component. It is likely that the TOC-TIC supercomplex represents only a minor subpopulation when purified via TOC components, but becomes enriched when isolated via TIC components. Therefore, statements such as "a clear and remarkable distinction from the algal TOC apparatus" and "plant and algal protein import systems are substantially different" should be revised to reflect these methodological differences and limitations.

(2) Before concluding that "affinity-purified TOC-P complexes showed negligible interaction with inner membrane TIC components," the authors should perform immunoblotting or mass spectrometry to detect TIC proteins such as Tic110 or Tic20.

(3) The authors should clarify how the identities of the protein bands visualized by Flamingo staining in Extended Data Fig. 8a were determined.

8. In Figs. 5b and 5c, the authors should clarify how the identities of the proteins detected by autoradiography were confirmed. Aside from the immunoblot in Fig. 1f, the composition of the TOC-P complex has not been comprehensively characterized. It remains unclear how many proteins were purified and in what relative amounts. The Coomassie-stained gel in Fig. 5c is extremely faint and lacks molecular weight markers, making it difficult to support the identification of the top two bands as Toc159 and Toc75. The authors state that Toc33 labeling could not be analyzed due to a co-migrating nonspecific band. However, a similar co-migrating band appears in the wild-type sample at the position labeled as Toc75, albeit at reduced intensity. This raises the question of whether the band labeled as Toc75 is indeed Toc75, or how much of it corresponds to Toc75.

9. The model for stepwise assembly of TOC complexes (Toc75 first, followed by Toc33, and then Toc159) is the same as the one proposed by Richardson et al. in 2014 (doi: 10.3389/fpls.2014.00269), based on earlier data. Multiple studies prior to 2014 have shown that the GTPase domain of Toc33 is required for the targeting and assembly of Toc159, indicating that Toc33 must assemble with Toc75 before Toc159. What was not previously known was how Toc33 assembles with Toc75. The current manuscript does not provide any new insight into this, as both toc75 and toc33 mutants reduce the abundance of all components, and the in vivo radiolabeling experiment did not allow for Toc33 analysis.

Minor Comments:
1. The age of the plants shown in Fig. 1b and Supplementary Fig. 2 should be indicated.

2. The meaning of the 200%, 100%, and 50% values in Fig. 1e, Fig. 4b, and Supplementary Fig. 1 should be clarified. For example, in Fig. 1e, does “100%” for roots and etiolated seedlings refer to an equal amount by fresh weight, or by micrograms of extracted protein?

3. For more accurate comparison of TOC protein abundance across tissues and mutants, internal controls such as actin or histone H3 (as used in Fig. 4) should also be included in Fig. 1e, Fig. 2a, and Supplementary Fig. 1.

4. The specific Rubisco subunit detected in Supplementary Fig. 1 should be identified (large or small subunit).

5. The naming of HA-Toc75 transgenic lines should be consistent. In Fig. 1, the lines are labeled HA-Toc75 #2 and #3, while in the Methods section, Fig. 4, and Extended Data Fig. 9, they are referred to as Ox-HA-Toc75 #1 and #2. This inconsistency should be addressed. The specific HA-Toc75 line used for TOC-P purification for negative-stain EM (Fig. 2e, Extended Data Fig. 1), in vivo labeling (Fig. 5), and Extended Data Fig. 8 experiments should also be clearly stated.

6. The number of cysteine and methionine residues in Toc75 is inconsistent: the main text (line 326) states 20, while the Figure 5 legend (line 573) lists 21. Additionally, for Toc159, the count of cysteine + methionine residues is 26 if the initiating methionine is included, not 25 as stated. This discrepancy should be clarified.

7. In line 233, the authors state that the Toc75–Toc159 complex “resembles the hybrid β-barrel of a BamA–substrate complex (Extended Data Fig. 5f).” In addition to reference 32, the authors should cite Jin et al. (Cell, 2022, https://doi.org/10.1016/j.cell.2022.10.030), which was the first report of this structural resemblance.

8. In line 234, the authors state: “A part of an embedded segment of Toc159 extends across this junction and underneath the Toc75-side of the β-barrel (Extended Data Fig. 5c,d). This overall arrangement of the hybrid β-barrel likely allows the protein import channel to expand and contract flexibly in order to accommodate (un)folded protein domains of different sizes.”

(1) In Extended Data Fig. 5c,d, the “embedded segment” of Toc159 should be shown in a distinct color to better differentiate it from other parts of Toc159. Also, note the inconsistency between the singular “segment” used in the text and the plural “segments” in the figure legend.

(2) The rationale for the claim that this hybrid β-barrel arrangement allows flexibility in channel size needs clarification. According to the Chlamydomonas TOC-TIC structure, the two sides of the hybrid barrel are stabilized by strong hydrogen bonds and hydrophobic interactions. Are hybrid β-barrels generally known to be flexible or capable of expansion/contraction? If so, please cite references; otherwise, the authors should explain the basis for this hypothesis or rephrase accordingly.

9. Typos:
Line 197: Should read "(Toc33-GTP2: KSS [aa 49–51])" instead of "[49–50]".
Line 927 and Fig. 5a: The light intensity unit should be “$\mu mol\ m^{-2}\ s^{-1}$,” not “$\mu mol\ m^{-2}\ s^{-2}$.”
Line 928: The concentration of Na_2SO_4 should be expressed in mM, not “Mm.”

Reviewer #2

(Remarks to the Author)
Nellaepalli and colleagues has combined various experimental approaches to demonstrate the presence of two distinct

forms of protein translocon at the outer envelope of chloroplast, namely TOC-P and TOC-N, which participate in import of different types of preproteins, namely photosynthetic and non-photosynthetic proteins, respectively. While the receptor GTPases included in TOC-P and TOC-N are different from each other, the central component TOC75 is commonly used and appear to be a key component for the assembly of these two distinct TOC isoforms. Thus study already contains enormous amounts of experimental data, which might be acceptable for publication without any revision. However, there still seems to be some concerns that the authors might need to address or reconsider.

The manuscript results section contains, as a considerably large part, AF3-predicted TOC structures and discussions based on this prediction. Although the authors have tried to add several experimental as well as MD data to support this prediction. However, the prediction still remains as a prediction. So, some of the descriptions should be transferred to discussion section or to supplementary discussion.

In relation to this notion, they describe in the text that “Comparing the different structures, we found that TOC-P from A. thaliana resembles the cryo-EM and AF3-predictedstructures of the algal TOC complex, indicating that structural organization of the TOC complex is broadly conserved in plants and green algae”. This seems to be a bit peculiar. While this reviewer is not sure when the authors actually performed this AF3 prediction, this was carried out probably after Chlamydomonas TOC-TIC supercomplex cryoEM structures had been available in public. This means AF3 predicted the TOC-P and TOC-N structures based on the deposited experimentally determined Chlamydomonas TOC structure as a kind of template as well as some typical GTP-binding proteins for GTPase domains of TOC receptors which were invisible for the Chlamydomonas TOC.

If my memory is correct, the reported Chlamydomonas TOC contains several previously unidentified TOC components associated with the TOC core and most strikingly the peculiarly integrated protein strands derived from the largest component of TIC, namely Tic214. However, the present discussion or description in Results section has not at all any mention for those. This reviewer would like to know how the spaces in the AF3 predicted structure corresponding to those occupied by Tic214 strands or by other TOC components are filled with what(?) or remain completely empty. More detailed structural comparison between the Chlamydomonas entire TOC and the AF3 predicted TOC with only TOC75 and two GTPase receptors.

Additional obvious lack of this study may be MS investigation of purified TOC75 fractions, TOC159 fraction, and TOC33, fractionm, which will tell us whether the fractions only contain TOC75 and two TOC GTP receptors proteins as the authors have proposed entirely in this study or contain any other proteins which might have been missing in their AF3 predictions.

The predicted close contact between the two GTPase domains of receptors is interesting. But again, this still seems to be as a predicted interaction. Although they performed XL-MS of the purified complexes, have they identified actual XL peptide(s) derived from both GTPase domains?

Minor points:

Why their roots fraction contains visible amount of Rubisco?

The TOC and TIC may be separately purified from Arabidopsis. But there seems to be some TIC component bands in the immunoblots of purified TOC fractions. Have they tried different conditions, e.g. different detergents, for TOC-TIC supercomplex purification?

Reviewer #3

(Remarks to the Author)
I co-reviewed this manuscript with one of the reviewers who provided the listed reports. This is part of the Nature Communications initiative to facilitate training in peer review and to provide appropriate recognition for Early Career Researchers who co-review manuscripts.

Reviewer #4

(Remarks to the Author)
In this manuscript, Nellaepalli et al. report the assembly of TOC complexes from photosynthetic chloroplasts and non-photosynthetic plastids. The authors revealed that TOC75 is the key component of TOC complexes and acts as the driving force behind its assembly. By integrated cross-linking, genetic, and in vivo labeling approaches, they successfully characterized structure of TOC-P. They also showed that TOC-N shares similar overall structure of TOC-P while exhibiting distinct differences. My comments primarily focused on the cross-linking experiments as below.

1.The authors only identified intra-protein crosslinks. The identification of inter-protein cross-links would significantly enhance the structural characterization of the TOC complex. The use of enrichable cross-linkers could facilitate the detection of such inter-protein interactions (Tan D. et al., eLife, 2016; Jiang P.L. et al., Angewandte Chemie, 2021).

2.Integrating cross-linking data with AlphaFold predictions has the potential to improve the accuracy of TOC complex structure modeling (Stahl K. et al., Nature Communications, 2024; Stahl K. et al., Nature Biotechnology, 2023). Such a combination is recommended to strengthen structural insights.

3.In Supplementary Fig. 8, the labeling styles of the mass spectra are inconsistent. Standardizing the labeling format across all spectra would improve clarity and readability.

4.For cross-link #4 in Supplementary Fig. 8, the number of assigned peaks is limited, particularly for the beta chain of the cross-linked peptides. Generally, the identification of cross-linked peptides is evaluated based on the following criteria: (1) Continuous fragment ions should be assigned to both the alpha and beta chains of the cross-linked peptides; (2) All major/strong peaks in the spectrum should be matched to the cross-linked peptides; (3) The mass spectrum should exhibit a high signal-to-noise ratio. Based on these standards, the identification quality of cross-link #4 appears to be low.

5.According to my knowledge, more cross-linked peptides should be identified for TOC complexes. Maybe, the authors could optimize their cross-linking procedures.

6.In page 41, line1000, the authors describe their cross-linking conditions: "The HA-purified TOC complex was crosslinked with 3 mM bis(sulfosuccinimidyl)suberate (BS3) (Thermo Scientific) at room temperature for 3 h". Typically, cross-linking reactions with BS3 are performed using 1 mM BS3 for 1 hour. The crosslinking conditions used here may lead to over-crosslinking, potentially resulting in non-specific cross-link identifications.

7.For cross-linking mass spectrometry experiments, have the authors performed technical replicates or biological replicates? Replicates are essential to validate data reproducibility and ensure overall data quality.

8.Depositing mass spectrometry data into public database (such as proteomeXchange) is encouraged

9.It is good to label the Cα-Cα distances of crosslinking sites on Fig. 3g.

10.Supplementary Table 1 should be expanded to include additional key information, such as scores and Cα-Cα distances

11."pLINK3" should be "pLink 3".

12."in vivo" should be "*in vivo*".

Reviewer #5

(Remarks to the Author)
This study combines biochemical analysis with structural modeling to provide insights into the TOC-P and TOC-N complexes from Arabidopsis. While this study is certainly interesting, there are several concerns regarding the modeling results, given that they form a key component of this study. Specific comments are given below.

1. The authors note that the AF3 models for the TOC-P and TOC-N complexes are "high confidence" (lines 149-150, 177-178), but the ipTM scores are not high (0.72, 0.70). As noted by the AlphaFold developers on the AlphaFold3 server site (https://alphafoldserver.com/faq), "ipTM values between 0.6 and 0.8 are a gray zone where predictions could be correct or incorrect." The authors should adjust their language and descriptions to note that the models have moderate rather than high confidence scores.

2. Rather than or in addition to the PAE plot of the AF3 model shown in Fig. 3b, showing the modeled structure colored by pLDDT scores (b-factor of the model) would be helpful to show readers the local and residue-level confidence levels in the structural model. Such a structural representation should be added.

3. In Fig. 3j regarding the MD simulation results, it is suggested that the authors use units of Angstroms rather than nanometers, as that is a much more commonly used unit for protein structural analysis and would be more familiar to readers.

4. The rationale and interpretation of the Phyre2 homology model of Toc159 noted on lines 442-446 should be described by the authors more, at least briefly. The structural agreement between the identified template structure (Tom40) and the homology model of Toc159 seems to be a direct result of the homology modeling procedure, which by definition will generate models that will resemble the template. Thus, this does not seem to represent robust evidence of actual structural similarity between the proteins.

5. While AF3 is a powerful modeling approach that can generate accurate models of monomers and multimers outside of its training set, one potential source of bias of the AF3 modeling is the use of template structures for individual chains, as well as PDB structures that have been seen during AF3 model training (structures before Sept. 30, 2021 PDB release date). This could in principle lead to high observed structural similarities with older PDB structures, for instance what is noted in Supplemental Figure 4. The authors should clearly note where the AF3 modeling is first described in the Results that the AF3 server uses PDB structural templates in modeling, along with the template date cutoff. These possible sources of bias in the models should also be noted in the Discussion.

Reviewer #6

(Remarks to the Author)
This manuscript provides a detailed characterization of two distinct TOC complexes, TOC-P from photosynthetic

chloroplasts and TOC-N from non-photosynthetic plastids, and identifies Toc75 as a central hub in TOC biogenesis. The integrative structural analysis and functional assays presented here advance our understanding of plastid protein import machinery. The work is timely, addresses an important knowledge gap, and is overall well-executed. However, several major points require clarification, additional data, or more rigorous interpretation before the manuscript can be accepted.
Major Comments
1. The structure shown in Fig. 3c suggests a very tight interaction between Toc75 and Toc159. This makes it puzzling why toc159 knockdown does not affect the protein level of Toc75 or Toc33. The authors should reconcile this apparent discrepancy, either with additional experimental data, literature context, or a discussion of potential compensatory mechanisms.
2. The authors believe that no experimental structure is provided for TOC-N due to flexibility seen in MD simulations. However, the reported flexibility does not appear particularly prominent. Please explain more clearly why structural characterization was not possible, was this due to sample heterogeneity, instability during purification, or technical limitations?
3. The integrative structural analysis is a strength of the paper, yet the specific key differences between TOC-P and TOC-N remain insufficiently clear. The authors should explicitly describe which structural features are unique to each complex and how these may relate to the observed functional differences.
4. In Fig. 3e, the single negative-stain EM 2D class shown for At-TOC-P is not convincing. Please provide a panel showing all 2D classes alongside a representative raw micrograph so readers can assess particle quality and sample heterogeneity.
5. In Suppl. Fig. 2, toc159 knockdown phenotypes appear more prominent than those of toc75, despite the conclusion that Toc75 is the key player in complex stability. This discrepancy requires explanation.
6. Can Toc75 and Toc33 form a stable complex in the absence of Toc159? If experimental data are unavailable, have the authors attempted to predict such a structure using AlphaFold 3?
7. Fig. 2's immunoblotting results are clear. However, the conclusions could be strengthened by complementary immunofluorescence to confirm protein localization and abundance in situ.
8. The microscopy-based ratiometric protein import assay in ko-toc33 and ko-toc34 mutants is elegant, but as it relies on protoplasts, the authors should discuss whether results may differ in whole tissue, given potential physiological differences.

Minor Comments
1. Line 149:"AF3 predicted the organization of the TOC-P complex with high confidence (ipTM = 0.72; pTM = 0.74)": An ipTM of 0.72 falls into the AlphaFold "gray zone" and indicates only moderate confidence in the predicted interface. Please acknowledge this limitation and, if possible, provide supporting evidence from additional models or experiments.
2. Line 202: Regarding Toc33/Toc34 homodimerization: Is there any known physiological function for this homodimer in vivo? If unknown, please state explicitly.
3. Line 282: Toc75 expression parallels LHCB1 induction, whereas other TOC genes are only moderately upregulated. Could Toc75 have additional roles beyond its function in TOC-P and TOC-N complexes?
4. Fig. 5b: It is unclear whether the purified chloroplasts shown are intact with outer membranes. Please provide a marker for the outer chloroplast membrane to confirm.

Version 1:

Reviewer comments:

Reviewer #1

(Remarks to the Author)
The authors have appropriately addressed most of my previous concerns, and the manuscript has been substantially improved. I have only a few remaining comments that can be addressed through rewriting.

1. Concerning my previous major comment 7 (current manuscript, "Conserved and distinct features of the TOC complex in plants and algae," line 247):
The authors cite a reference reporting purification of a TIC complex without TOC proteins (Kikuchi et al., 2013). I would like to remind the authors that these experiments were performed under extremely high salt conditions (1 M NaCl). Even considering this reference, there are substantial published data showing that TOC proteins can co-immunoprecipitate TIC proteins and vice versa, and that the amount of co-immunoprecipitated protein does not change in the presence or absence of bound precursor proteins. These findings suggest that stable TOC–TIC complexes can exist even in the absence of translocating precursor proteins (Akita et al., 1997, J. Cell Biol. 136:983–994; Nielsen et al., 1997, DOI: 10.1093/emboj/16.5.935; Kouranov et al., 1998, J. Cell Biol.).

Notably, the authors' Fig. R5 (Extended Data Fig. 9a in the current revised manuscript) shows the presence of TOC proteins in fractions 14–18, which are the major fractions containing Tic214/56/100. This result could indicate that an independent TOC complex is present in fractions 9–12, while fractions 14–20 contain a TIC–TOC supercomplex, or a combination of a TIC–TOC supercomplex and a TIC complex. This interpretation is consistent with the results of Akita et al. (1997) and Chen and Li (2017; DOI: 10.1111/tpj.13643).

It is possible that, when pull-downs are performed using a TOC protein, the TOC complex is preferentially isolated because most TOC proteins reside in the TOC complex, with only a smaller fraction present in the TIC–TOC supercomplex. Conversely, when pull-downs are performed using a TIC component, as in the Chlamydomonas studies, the TIC–TOC

supercomplex may be preferentially purified because a larger proportion of TIC proteins are present in that complex.

In this section of the Results (“Conserved and distinct features of the TOC complex in plants and algae,” line 247) and based on Extended Data Fig. 9, the authors conclude that “TOC and TIC components form distinct complexes of different sizes (~300 and ~600 kDa, respectively), suggesting weak or transient interactions between the TOC and TIC complexes in plants.” This conclusion is not supported by the data. The results show only that a greater proportion of TOC proteins is present in the ~300 kDa fractions and a smaller proportion in the ~600 kDa fractions. This is a matter of relative abundance and does not reflect interaction strength. Accordingly, this section should be re-written and Extended Data Fig. 9e should be removed.

2. Concerning my previous major comment 9:
The new data on TOC-iP are interesting. However, this reviewer still thinks that the review by Richardson et al. (2014; reference 17 in the current manuscript) should be cited when discussing Fig. 6 in the Discussion section. Citing this work would not diminish the novelty of the current study, but would appropriately acknowledge that a similar model has been proposed previously.

3. Inconsistency in naming of HA-Toc75 transgenic lines:
The nomenclature used for the HA-Toc75 transgenic lines is inconsistent and should be corrected.
Line 328: “(HA-Toc75, #2 and #3)” versus line 333: “in the Ox-HA-Toc75 lines”
Figure 4d,e: “Ox-HA-Toc75 #1, #2” versus the Fig. 4d,e legend: “HA-Toc75 over-expressor plants”

Reviewer #2

(Remarks to the Author)
Nellaepalli et al. now have extensively and adequately revised their previous manuscript with enormous amounts of additional high quality data in order to address most of the concerns raised by all the reviewers. I have no more concerns on this revised version. With these alterations, this manuscript will surely advance our understanding of assembly of TOC involved in chloroplast protein import.

Reviewer #3

(Remarks to the Author)
I co-reviewed this manuscript with one of the reviewers who provided the listed reports. This is part of the Nature Communications initiative to facilitate training in peer review and to provide appropriate recognition for Early Career Researchers who co-review manuscripts.

Reviewer #4

(Remarks to the Author)
Based on my knowledge, the cross-linked peptides are difficult to be extracted from SDS-PAGE gel,which often results in lower identification efficiency for in-gel digested cross-linking samples compared to in-solution digestion. I have no other comments.

Reviewer #5

(Remarks to the Author)
The authors have addressed most comments satisfactorily. While it is helpful that the authors have now colored the models by pLDDT score in Extended Data Fig. 2 and elsewhere, the scale bar annotating the colors does not give any values at all and just notes "min" and "max" for the pLDDT range. Thus the pLDDT values could hypothetically range for instance from 20-50, or from 50-95, which would be a big difference. For Extended Data Fig. 3, in contrast, the pLDDT colors are more helpfully annotated. In Extended Data Fig. 2 and other figures with pLDDT color keys, the authors should instead note the pLDDT values for "min" and "max" for the model shown, or if they prefer, use a convention like in Extended Data Fig. 3. This would help readers to better understand both the pLDDT variability as well as the overall range of values being represented.

Reviewer #6

(Remarks to the Author)
The authors have adequately addressed all of my points, and I have no further comments or suggestions.

Open Access This Peer Review File is licensed under a Creative Commons Attribution 4.0 International License, which permits use, sharing, adaptation, distribution and reproduction in any medium or format, as long as you give appropriate credit to the original author(s) and the source, provide a link to the Creative Commons license, and indicate if changes were made.
In cases where reviewers are anonymous, credit should be given to 'Anonymous Referee' and the source.
The images or other third party material in this Peer Review File are included in the article’s Creative Commons license, unless indicated otherwise in a credit line to the material. If material is not included in the article’s Creative Commons license and your intended use is not permitted by statutory regulation or exceeds the permitted use, you will need to obtain permission directly from the copyright holder.
To view a copy of this license, visit https://creativecommons.org/licenses/by/4.0/

REVIEWER COMMENTS

Reviewer #1 (Remarks to the Author):

In this manuscript, the authors examined and compared the protein abundance of various TOC and TIC components across different tissues, in various toc mutants, and in seedlings undergoing the de-etiolation process. They also created an HA-Toc75 transgene to rescue the pale-green kd-toc75 (mar1) mutant and used HA-Toc75 plants for affinity purification and in vivo labeling to investigate the sequential assembly of the TOC-P complex. By combining these experiments with structural predictions of TOC complexes using AlphaFold3 (AF3), molecular dynamics (MD) simulation analyses, and in vivo import assays, the authors conclude that they have revealed the presence of two distinct TOC complexes—TOC-P and TOC-N—with unique structural organizations and functions. They also propose that Toc75 serves as the central hub and driver in TOC complex biogenesis.

The manuscript presents substantial data supporting the existence of the TOC-P complex. The use of AF3 and MD simulations suggests the presence and importance of two linker regions in TOC structural dynamics. Observed changes in protein abundance allow reasonable inference for Toc75 functioning as the primary driver of TOC assembly. These results offer further insights into the existence and functions of multiple TOC complexes. However, the concept of distinct TOC complexes—one associated with photosynthetic substrates and another with non-photosynthetic or housekeeping substrates, each preferentially distributed in photosynthetic and non-photosynthetic tissues—has been previously demonstrated by multiple groups, including the authors' own earlier work. Notably, Ivanova et al. (MBC 15, 3379–3392, 2004) used immunoprecipitation to show that Toc159 preferentially forms complexes with Toc33 and Toc75, while Toc132 preferentially associates with Toc34 and Toc75.

Response: First, we thank the reviewer for these positive comments on our work. We are especially grateful for the constructive comments on how the manuscript may be further improved. We have addressed all of the issues satisfactorily in this revision, based on the reviewers suggestions, and believe that the manuscript is now very much improved.

We of course agree that our study builds on initial insights from previous work, such as the mentioned Ivanova et al. report[1]. In that study, immunoprecipitation analysis of wild-type plants using anti-Toc120, -132 and -159 antibodies supported the proposal that these TOC receptors exhibit association preferences. Our manuscript takes things further by using transgenic plant lines expressing functionally-verified transgenes (HA-Toc75, TAP-Toc33, TAP-Toc139) to perform robust affinity chromatography analyses. Using this approach, we demonstrated the differential accumulation of two distinct TOC complexes—TOC-P and TOC-N—in green and non-green tissues (containing chloroplasts and leucoplasts, respectively). We have additionally conducted mass-spectrometry analysis of HA-Toc75 affinity purifications to reveal the abundance of TOC-P in leaves (Fig. R1), and these data are shown in the revised manuscript (Extended Data Fig. 1; and the related mass spectrometry data are included as Supplementary Table 1). These data support the description of TOC-P composition already included in the manuscript. Moreover, unlike previous studies[1-4], our study provides direct evidence that Toc75 is a central player in

forming at least two distinct TOC complexes, coordinating complex dynamics in different plastid types including chloroplasts, leucoplasts and etioplasts.

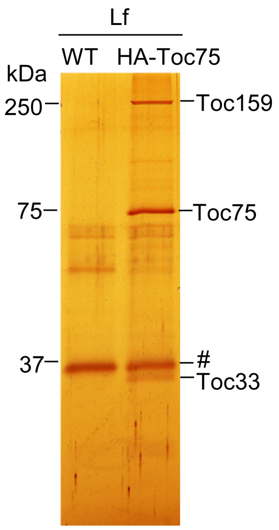

Fig. R1. *The TOC-P complex is the major TOC complex in chloroplast-enriched leaf samples. Polypeptide composition of the affinity-purified HA-Toc75 complexes, alongside a wild-type sample included as a negative control. Polypeptides were visualized by silver staining. Identities of the strongly-stained bands were determined by in-gel digestion mass-spectrometry; and in addition, the affinity-purified HA-Toc75 complexes were analysed by in-solution digestion mass-spectrometry (Supplementary Table 1). A non-specific band is indicated (#).*

Additionally, a model for stepwise TOC complex assembly (Toc75 followed by Toc33, then Toc159) has already been proposed and partially validated. Several parts of the manuscript also require more detailed explanation, and some interpretations of the data appear overstated and should be revised. Detailed comments follow below.

Response: We agree that there are some previous studies that provided important insights concerning TOC assembly (e.g., [4-6] in this document). Although these papers were cited in the original manuscript (in the Discussion, lines 422-428), we accept that more could have been done to highlight their relevance, and so we have expanded our explanations in the revised text.

Firstly, we have cited the key Wallas et al.[4] study in the Introduction (ref. 16, line 68), as it was previously mentioned only in the Discussion.

Secondly, we have explained the relevance of these studies in the Results section as follows "*These observations align with previous data*[16,45,46] *obtained through analysis of in vitro translation products with reconstituted proteoliposomes showing that the insertion of Toc159 requires the actions of Toc75 and Toc33*" (this is in a new Section entitled "*Identification of a TOC intermediate complex, TOC-iP*" on page 10).

However, we would like to emphasize here that those earlier studies were based exclusively on in vitro analysis using isolated components, and so, as the reviewer notes above, they were not able to deliver conclusive hypothesis validation. In the previous studies, in vitro reconstitution experiments suggested that Toc75 and Toc33 are required for Toc159 integration. In our study, we employed native protein biochemistry and genetic analyses to

provide a more comprehensive and physiologically-relevant picture of TOC assembly, delivering a deeper and more robust understanding of the assembly process. Thus, thirdly, we elaborated on these points in the fourth paragraph of the revised Discussion.

Major Comments:
1. Using Fig. 1e and Supplementary Fig. 1, the authors conclude (line 88):
"Receptor isoforms of Toc33 and Toc159 were enriched in leaves, whereas Toc34 and Toc132/120 … were enriched in roots and petals. All TOC components were present at relatively low levels in etiolated seedlings …, revealing clear differentiation of TOC composition in different plastid types."

To support these conclusions, the authors should specify the total protein amount loaded per lane, include internal loading controls (e.g., housekeeping proteins), and provide quantitative analyses of the immunoblot data. For example, in Fig. 1e, the immunoblot signal intensities for Toc159 in the 50% and 100% protein loadings of etiolated (Et) samples appear nearly identical. In Supplementary Fig. 1, the Toc159 signal in leaf (Lf) samples is also comparable regardless of protein loading.

Response: Thank you for this helpful comment. We agree that immunoblot signal intensities can sometimes be difficult to compare, such as with low amounts of protein. To overcome these difficulties, we have reanalysed our data as follows:

First, we loaded samples based on equal fresh weight of the starting sample material, and then compared the signals for Toc75 by immunoblotting. Second, we re-loaded the samples to deliver equal Toc75 signals in all three samples, including the dark-grown seedlings which are enriched in etioplasts. Third, we verified that the protein abundance profiles were closely comparable across all three relevant tissue types by using CBB staining (note: RbcL was excluded from consideration since it is absent in root samples).

Once these three things had been achieved, we repeated our analysis (Fig. R2 below). Overall, our reanalysis showed that TOC-P receptor components are enriched in leaves, and that TOC-N receptor components are enriched in roots. These results are in fact consistent with what was shown in the first version of the manuscript (in Fig. 1). Interestingly, the improved sample normalization in our new analysis (Fig. R2) revealed that both TOC-P and TOC-N are present at appreciable levels in the etioplasts indicating the pre-existence of the TOC components even before the establishment of photosynthesis. These new data have been added to the revised manuscript (replacing panel e in Fig. 1). Please note that standard loading controls like actin cannot be used here for sample normalization, because of the huge proteome differences between the analysed tissues.

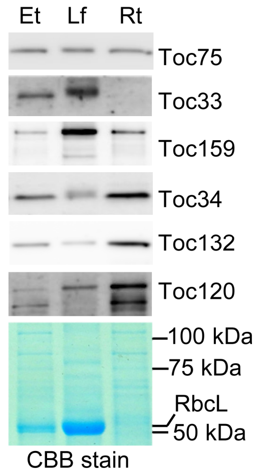

Fig. R2. Immunoblotting analysis of protein extracts from dark-grown wild-type seedlings containing etioplasts (Et); from chloroplast-enriched leaf fractions (Lf); and from roots containing leucoplasts (Rt).

In Supplementary Fig. 1, the "Rubisco" signal for the 50% Lf sample is stronger than the signal of the 100% sample, which raises concerns about linearity and normalization.

Response: Thank you for point this out. The immunoblotting signal for RbcL was not displaying linearity due to saturation owing to the very high abundance of this protein. To resolve this problem, we have included CBB staining of the gel, which clearly shows linearity when comparing the 100% and 50% loadings (Fig. R3 below). In parallel, we removed the problematic RbcL immunoblotting panel. Moreover, this staining image clearly indicates that we have comparable loadings of the leaf and petal samples. In summary, we think that linearity and normalization were both good in the original data, and that this is supported and better demonstrated by the newly added CBB staining image.

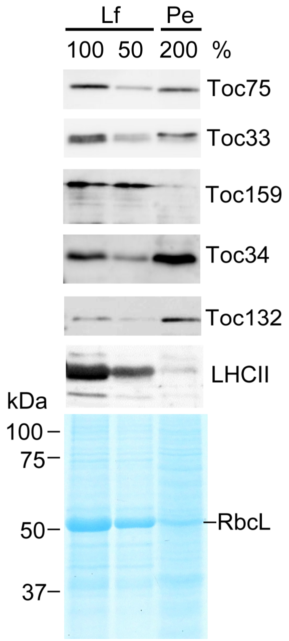

Fig. R3. *Total protein extract from the petals (Pe) of 6-week-old WT plants was analysed by immunoblotting alongside the chloroplast-enriched leaf fraction (Lf) described in Fig. 1e in the manuscript. Twice the amount of the petal sample (200% based on fresh weight) was needed to achieve normalized levels of the Toc75 component, and of the overall proteome as revealed by CBB staining. Two different loading amounts (%) of the Lf sample are shown.*

Furthermore, in Supplementary Fig. 1, the Toc34 signal is indeed stronger in petals than in leaves, but since twice the amount of petal proteins was loaded, enrichment in petals cannot be concluded without normalization.

Response: We apologize for the lack of clarity here. The samples were loaded on a tissue fresh weight basis, not according to protein amounts. We found that it was necessary to load twice the amount of the petal sample relative to the chloroplast-enriched leaf sample (i.e., 200% on a per fresh weight basis) in order to achieve normalized levels of the Toc75 component. This was noted in the relevant figure legend, but we accept that this was not sufficiently clear. We have edited the legend to improve its clarity.

Moreover, as noted in our response above, we now include CBB staining of the gel, which very clearly shows that the protein loadings of the leaf (100%) and petal (200%) samples were similar (Fig. R3 above). Thus, it is clear that the observed higher levels of Toc34 in petals are not due to sample overloading.

2. Regarding Fig. 1f (HA-Toc75 purification), the authors state (line 96): "The analysis showed that Toc75 is mainly associated with Toc33 and Toc159 in leaf chloroplasts, and to a lesser extent with Toc34, Toc132, and Toc120. In contrast, Toc75 is mainly associated with Toc34, Toc132, and Toc120 in root leucoplasts, and to a lesser extent with Toc33 and Toc159." This conclusion is further illustrated schematically in Fig. 1g.

(1) The data in Fig. 1f only support the conclusion that TOC-P is more abundant in leaves than roots, and TOC-N is more abundant in roots than leaves. The data cannot be used to compare the relative abundance of TOC-P versus TOC-N within the same tissue, as immunoblot signals for different proteins are not directly comparable. Variability in antibody affinities, epitope accessibility, protein transfer efficiency, and chemiluminescence detection time must be considered. To support the conclusions in Fig. 1g, additional experimental controls are needed—for instance, quantification by comparing to known amounts of recombinant proteins to allow accurate comparison between different proteins.

Response: Many thanks for this important comment. We agree that the immunoblotting data cannot be used to compare the relative abundancies of proteins within the same samples, without reference to known standards. To overcome this problem, we stained a TOC purification from a chloroplast-enriched sample to show the polypeptide profile of the TOC components and subsequently analysed the sample by mass spectrometry (Fig. R1 above). These results show clear enrichment of Toc159 relative to Toc132/120. This provides clear evidence that TOC-P is more abundant in leaves than TOC-N. These data have been included in the revised manuscript (Fig. R1a has become Extended Data Fig. 1; and the related mass spectrometry data are included in Supplementary Table 1). Moreover, we edited the text to make it clear that only appropriate comparisons were made (see third and fourth paragraphs of the Results, page 4). The model in Fig. 1g was accordingly revised to

emphasize only appropriate comparisons (i.e., TOC-P enrichment in leaves compared to roots; TOC-N enrichment in roots compared to leaves).

(2) The data also do not rule out the possibility of a Toc34–Toc75–Toc159 complex, nor do they confirm the proposed Toc75–Toc34–Toc132 (TOC-N) complex. To validate the presence of the TOC-N complex, immunoprecipitation using Toc132/Toc120 and Toc34 as bait proteins should be conducted.

Response: Thank you for this comment. We agree that this is an important point, but actually we believe that our affinity purification data in Fig. 1f already argued strongly against the existence of putative Toc159-Toc34-containing complexes; and instead supported the existence of Toc132-Toc34-containing TOC-N complexes, especially in roots. Nevertheless, in further support of this conclusion, we refer here again to the new staining and mass spectrometry analysis presented above (Fig. R1). These data clearly demonstrate co-enrichment of Toc159 and Toc33 in the purified HA-Toc75 complexes, with the Toc132, -120 and -34 being undetectable by staining, and present at low levels according to mass spectrometry. Similarly, in our TAP-Toc159 and TAP-Toc33 purifications (Extended Data Fig. 9b), staining of Toc132 and Toc120 was not detectable.

We appreciate the suggestion to conduct immunoprecipitation analysis using the TOC-N components as bait proteins. However, we unfortunately do not possess in sufficient quantities the specific antibodies needed for such analysis. We are in the process of generating HA-Toc34 and HA-Toc132 transgenic lines that could be used as an alternative strategy here, but this work extends beyond the scope of the present study as it will require another 6-8 months to complete; and in any case believe that the new data provided above substantially address the reviewer's concern, and are furthermore in accordance with previous results from Ivanova et al.[1]

3. In Fig. 2, the authors conclude that: "Toc75 and Toc33/34 are key players in stabilizing the integrity of the TOC-P and TOC-N complexes," based on immunoblot signals of TOC components in toc mutants. However, the current data primarily demonstrate that kd-toc75, ko-toc33, and ko-toc34 mutations reduce the abundance of individual TOC components, not the integrity of TOC complexes. To support conclusions about complex integrity, co-immunoprecipitation assays should be performed to test whether Toc75 still interacts with Toc33, Toc34, Toc159, and Toc132 in these mutant backgrounds.

Response: Thank you for raising this interesting question. Our affinity purifications in Fig. 1 clearly demonstrated the interactions between the relevant TOC components, and then the immunoblotting data in Fig. 2 revealed what happens to those interaction partners when one of the components of a particular complex is removed in different mutant genotypes. We inferred that secondary depletion of interacting proteins in the mutants is indicative of reduced complex stability or integrity, but accept that this was not directly demonstrated. We have therefore softened our conclusions on page 5 (second paragraph), making it clear that loss of complex stability is a hypothesis (and not excluding other possible interpretations of the data). We are grateful for the suggestion to perform additional immunoprecipitation experiments, but at this time we lack the necessary resources to do this in a timely way, and so feel that this falls outside of the scope of the present study.

4. The authors describe purification of the TOC-P complex from HA-Toc75 plants for use in negative-stain EM analysis (Fig. 3e and Extended Data Fig. 1).

(1) However, as shown in Fig. 1f and Supplementary Table 1, both TOC-P and TOC-N components are present in the purified sample. It is unclear how the authors selectively purified only the TOC-P complex for structural analysis. This point requires clarification.

Response: Thank you for raising this important issue. The new staining analysis of affinity-purified HA-Toc75 complexes from leaf chloroplasts (see Fig. R1 above) clearly shows that the sample is enriched in TOC-P, with TOC-N present only at low levels.

Moreover, for our negative-staining structural analysis (Fig. 3e), the affinity-purified samples were further separated by size exclusion chromatography, meaning that TOC-N was likely present at only negligible levels. Thus, our negative-staining results are highly likely to represent the TOC-P complex only. We cannot completely rule out that TOC-N is minimally represented in these results, but given that this complex is present at much lower levels in the samples, and furthermore is rather similar TOC-P (see Extended Data Fig. 4 of the revised manuscript), we do not think this is a significant concern for these low-resolution analyses. It is also noteworthy that the presented particle classes were selected based on the 2D projections of AF3-predicted TOC-P complex.

(2) Additionally, scale bars are missing in the EM images (Extended Data Fig. 1 and Fig. 3e). Their inclusion is necessary for interpretation.

Response: Please accept our apologies for this oversight. Scale bars are now shown in the EM images in the revised manuscript (see Fig. 3e and Extended Data Fig. 3a).

5. Referring to Extended Data Fig. 7, the authors state (line 252): “The missing TMH is needed to properly anchor and stabilize the Toc159 β-barrel in the membrane.” In the figure legend, they add: “The mutant protein was extracted much more readily than the WT protein, indicating instability.”

To strengthen this conclusion, the authors should provide immunoblots of the supernatant fraction following alkaline extraction, along with appropriate internal controls. These should include markers for soluble, peripheral, and membrane-associated proteins to validate extraction efficiency and normalize protein loading. Ponceau S staining has limited sensitivity; in particular, LHC staining is not visible in WT samples, making it unreliable for normalization.

Response: Thank you for this very helpful advice. As suggested, we repeated this analysis in order to test for the presence of Toc159 in the soluble fractions after alkaline treatment, and to add data for control proteins. Briefly, crude chloroplasts of WT and *kd-toc159* plants were subjected to alkaline treatment for 30 min, and subsequently separated into membrane and soluble fractions. Immunoblot analysis of the samples revealed reduced stability of the mutant Toc159 protein compared to that in WT, consistent with our observations in the original manuscript. Interestingly, signals corresponding to the destabilized Toc159 protein were not clearly detected in the soluble fractions, likely due to rapid degradation upon extraction. These new data are included in the revised manuscript as Extended Data Fig. 8.

6. AF3 uses structural data from known PDB entries—including the Chlamydomonas TOC-TIC supercomplex and Toc34/Toc33 dimers—to predict the Toc33–Toc75–Toc159 assembly. The authors state (line 263): “Comparing the different structures, we found that TOC-P from A. thaliana resembles the cryo-EM and AF3-predicted structures of the algal TOC complex, indicating that structural organization of the TOC complex is broadly conserved in plants and

green algae."

This raises concerns about circular reasoning, as the referenced structures were incorporated into the model generation itself. The authors should clarify how this potential bias was controlled or, alternatively, revise the statement to acknowledge the limitation.

Response: We are grateful to the reviewer for raising this very important point. Unlike structure prediction applications like Phyre2 and iTASSER, which use homology modelling based on a user provided protein sequence and a template (often an experimental Protein Data Bank [PDB] structure), AlphaFold (AF) needs only a primary amino acid sequence as an input. Therefore, it is important to stress that AF is not a homology modelling tool and can successfully predict 3D protein structures (including those with a novel fold) with no prior template structure. As an artificial intelligence tool, it has already learned folding patterns of proteins after being trained on experimental structures deposited in PDB up until September 2021. To predict a 3D structure, AF aligns an input protein sequence against thousands of sequences from various organisms, and works out co-evolutionary signals between each pair of residues and accurately maps them in 3D space using a neural network (e.g., if two regions of a protein have been evolving independently, they are probably not in a direct physical contact). Prediction of query structures by AF is not dependent on a PDB template, although such can be used to facilitate prediction in difficult cases.

Significantly, we ran the AF3 predictions of the Arabidopsis TOC complex structures using the AlphaFold3 server, either by using PDB templates with a default cut-off date of 30/09/2021, or without using PDB templates at all. In both cases our structure predictions converged to near-identical models of the TOC complexes with high confidence metrics as described. Moreover, the release of the algal TOC-TIC complex cryo-EM structures occurred *after* the configuration of the AF3, as the following dates clearly indicate.

The following are the training and inference regime cut-off dates for AF3:

- *Training regime:* No structural data used during training were released after **30 September 2021** and, for the model used in PoseBusters evaluations, PDB structures released after **30 September 2021** were filtered out[7].
- *Inference regime:* No inference time templates or reference ligand position features were released after **30 September 2021**, and in the case of PoseBusters evaluation, an earlier cut-off date of **30 September 2019** was used[7].

The following are the release dates of the algal TOC-TIC cryo-EM structures:

1. 7VCF (*Cell* 185: 4788-4800.e13 [2022]) release date: **30 November 2022.**
2. 7XZI (*Nature* 615: 349-357 [2023]) release date: **11 January 2023.**

In conclusion, we can confidently eliminate the concern about possible circular reasoning that was raised by the referee. We have provided clarification of these points in the revised manuscript (in the Methods).

7. Using Extended Data Fig. 8, the authors state (line 267): "However, affinity-purified TOC-P complexes from our HA-Toc75 lines, as well as from TAP-tagged transgenic lines (TAP-Toc159, TAP-Toc33), showed negligible interaction with inner membrane TIC components

(Extended Data Fig. 8). This revealed a clear and remarkable distinction from the algal TOC apparatus, which is tightly integrated with the TIC apparatus in an interlocked TOC-TIC supercomplex. This implies that the plant and algal protein import systems are substantially different."

(1) While this observation is intriguing, it is important to consider the effects of different detergent conditions on the integrity and composition of membrane protein complexes. In this study, 1% DDM was used to solubilize chloroplasts, whereas previous studies on the algal TOC-TIC supercomplex (e.g., doi.org/10.1038/s41586-023-05744-y and doi.org/10.1016/j.cell.2022.10.030) employed milder conditions, such as 1% LMNG supplemented with 0.2% digitonin or 0.12% CHS. These gentler detergents are known to better preserve labile or transient interactions between membrane complexes. Moreover, the current study used TOC components (HA-Toc75, TAP-Toc159, TAP-Toc33) as purification baits, whereas the algal complex was purified using a tagged TIC component. It is likely that the TOC-TIC supercomplex represents only a minor subpopulation when purified via TOC components, but becomes enriched when isolated via TIC components. Therefore, statements such as "a clear and remarkable distinction from the algal TOC apparatus" and "plant and algal protein import systems are substantially different" should be revised to reflect these methodological differences and limitations.

Response: This is another highly significant point. We agree that detergent choice can have a significant bearing on the results of such experiments. For this reason, we performed additional purifications using mild detergents, LMNG and α-DM, alongside a stronger detergent, Triton X-100. However, even under the mild conditions, co-purification of TIC components was very low (undetectable by staining) in comparison with the TOC components in these experiments (Fig. R4 below). Mass spectrometry analysis of our preparations performed using 1% β-DM confirmed that the TIC proteins were present only at low levels (see Fig. R1 above; and Extended Data Figs. 1 and 9 in the revision, with the related mass spectrometry data shown in Supplementary Table 1).

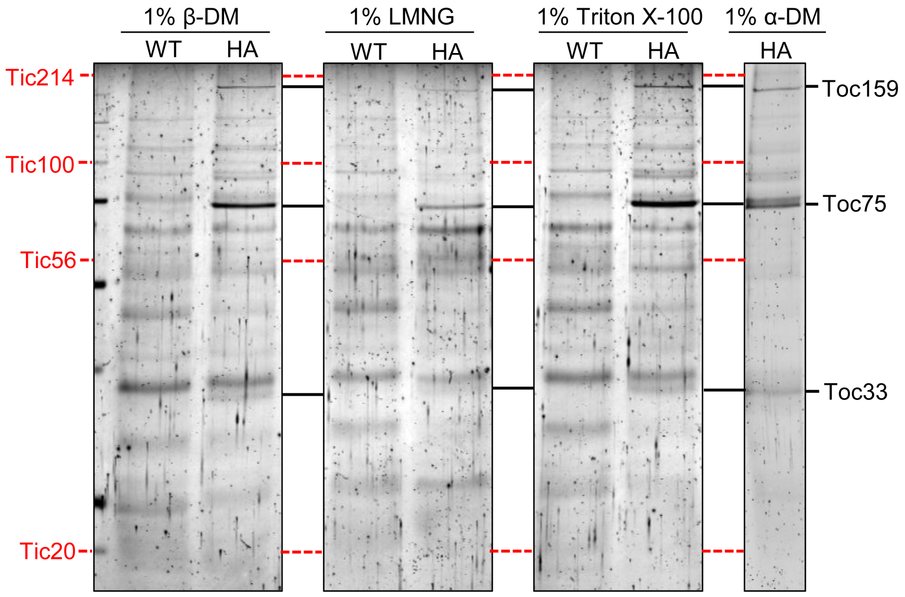

Fig. R4. Purification of TOC complexes under mild (1% β-DM, 1% LMNG) and stronger (1% Triton X-100) detergent conditions, alongside the detergent conditions used in the manuscript (1% α-DM). Gels were stained using Flamingo. Black lines indicate the positions of the TOC-P components, which were clearly detectable. Red dotted lines indicate the expected positions of TIC components based on their established migration properties, all of which were undetectable.

Moreover, we further addressed this issue by performing new density gradient ultracentrifugation experiments to elucidate TOC-TIC component interactions (Fig. R5 below). The results clearly reveal how the TOC and TIC components form distinct complexes of different sizes (~300 and ~600 kDa, respectively), supporting the notion that there are only weak or transient interactions between TOC and TIC complexes in Arabidopsis. These new data are now included in the revised manuscript (as Extended Data Fig. 9a). Moreover, this conclusion aligns with our proteomics analysis of purified HA-Toc75 complexes (see Extended Data Fig. 9d) and with the other data shown in Extended Data Fig. 9; as well as with the results of a previous study in which a tagged TIC component, Tic20, was used to pull down the whole TIC complex without co-purifying the TOC complex[8].

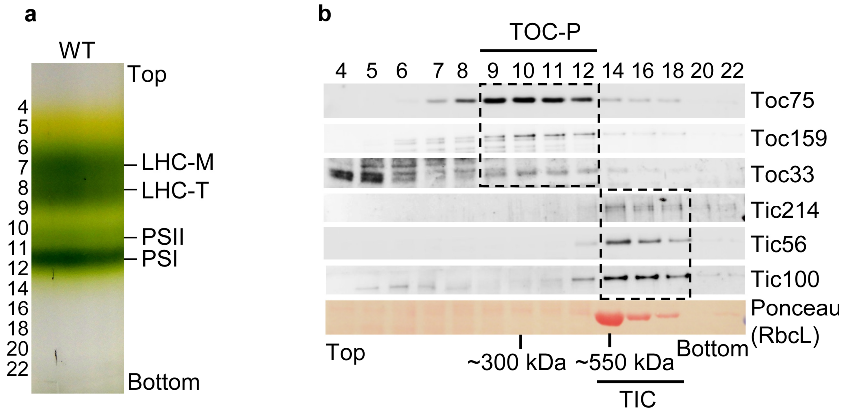

*Fig. R5. Separation of protein complexes in chloroplast-enriched leaf fractions of wild-type plants by sucrose density gradient ultracentrifugation. **a.** Image of the gradient showing the separation of pigment-protein complexes. **b.** Fractions from the gradient were analysed by immunoblotting and Ponceau staining.*

Based on these data, we feel confident that TOC and TIC are not deeply integrated as the published cryo-EM structures seem to show is the case in green algae. Nonetheless, we do accept that there are certain caveats with interpretation here, and so we have revised the text to moderate our conclusions as suggested by the referee, for example by using the word "weak" in place of "negligible" in relevant descriptions (i.e., the second and third paragraphs in the section entitled "*Conserved and distinct features of the TOC complex in plants and algae*", on pages 8-9).

(2) Before concluding that "affinity-purified TOC-P complexes showed negligible interaction with inner membrane TIC components," the authors should perform immunoblotting or mass spectrometry to detect TIC proteins such as Tic110 or Tic20.

Response: We have addressed this point thoroughly in our response to the previous point above.

(3) The authors should clarify how the identities of the protein bands visualized by Flamingo staining in Extended Data Fig. 8a were determined.

Response: The bands of interest (from HA-Toc75 preparations) were excised and analysed by mass spectrometry (see Supplementary Table 1 of the revision). This information has been added to the relevant figure in the revised manuscript to improve clarity (see Extended Data Fig. 1).

8. In Figs. 5b and 5c, the authors should clarify how the identities of the proteins detected by autoradiography were confirmed. Aside from the immunoblot in Fig. 1f, the composition of the TOC-P complex has not been comprehensively characterized. It remains unclear how many proteins were purified and in what relative amounts. The Coomassie-stained gel in Fig. 5c is extremely faint and lacks molecular weight markers, making it difficult to support the identification of the top two bands as Toc159 and Toc75. The authors state that Toc33 labeling could not be analyzed due to a co-migrating nonspecific band. However, a similar co-migrating band appears in the wild-type sample at the position labeled as Toc75, albeit at reduced intensity. This raises the question of whether the band labeled as Toc75 is indeed Toc75, or how much of it corresponds to Toc75.

Response: Thank you for raising the need for improved clarity here. We took great care to confidently assign the labelling of Toc75 and Toc159 in Fig. 5. As noted in our response to the point above, the identity of the proteins in our TOC-P purifications was verified by analysing excised gel bands using mass spectrometry. Moreover, the characterization of our preparations is now fully presented in this response (see Fig. R1 above) and included in the revised manuscript (see Extended Data Fig. 1 and Supplementary Table 1), as already noted earlier.

Regarding the referee's comment that there is a co-migrating band at the position of Toc75 in the wild-type sample (Fig. 5b): We are confident that there is no Toc75 in the wild-type negative control sample. The non-specific band that the reviewer mentioned actually migrates slightly faster than the Toc75 band in HA-Toc75 sample. Moreover, we verified that there is no detectable Toc75 in the wild-type control here by immunoblotting.

Regarding the further point that the band intensities here are weak: These samples are derived from very young seedling (7-8 days old), and the analysis was furthermore performed at a low scale (~40 seedlings) due to the need to conduct radiolabelling. Thus, it is entirely expected that the final yields here were low in comparison with our other preparations; these details are now explained in the revised manuscript (see paragraph 2 on page 10, second paragraph of the section entitled "*Toc75 synthesis precedes that of Toc159 in the TOC assembly process*"). Nonetheless, yields were sufficient to give detectable staining of Toc75 and Toc159 (by CBB), enabling confident assignment of the radiolabelled bands. We apologize that molecular weight markers were originally omitted in this analysis, and we have added them in the revised manuscript.

9. The model for stepwise assembly of TOC complexes (Toc75 first, followed by Toc33, and then Toc159) is the same as the one proposed by Richardson et al. in 2014 (doi: 10.3389/fpls.2014.00269), based on earlier data. Multiple studies prior to 2014 have shown

that the GTPase domain of Toc33 is required for the targeting and assembly of Toc159, indicating that Toc33 must assemble with Toc75 before Toc159. What was not previously known was how Toc33 assembles with Toc75. The current manuscript does not provide any new insight into this, as both toc75 and toc33 mutants reduce the abundance of all components, and the in vivo radiolabeling experiment did not allow for Toc33 analysis.

Response: Thank you for this comment. As noted earlier, previously proposed hypotheses were based on in vitro experiments, where the native conformations of TOC components were not fully validated[4-6]. In these studies, Toc33 was prepared by expression in bacteria, and Toc159 was synthesized using a rabbit reticulocyte in vitro translation system; such preparations were tested for binding and membrane insertion assays using isolated chloroplasts or proteoliposomes. Importantly, the native conformation of the TOC components was not tested. In contrast, in the present manuscript, to better understand the role of Toc75, particularly its native interactions during TOC assembly, we employed an integrative experimental approach, including mutant analysis (Fig. 2), gene expression analysis (Fig. 4a), in planta *TOC75* expression manipulation (RNAi and overexpression) (Fig. 4b,c), and in vivo labelling using inorganic sulphate ^{35}S (Na_2SO_4) (Fig. 5a-d). These experiments allowed us to confidently establish the native role of Toc75 as a central player in the TOC assembly process.

While we acknowledge that the in vivo radiolabelling experiment did not specifically dissect Toc33 assembly into the TOC complex, other aspects of our work (notably the *ko-toc33* analysis in Fig. 2) nevertheless suggest that Toc33 is important for the accumulation of TOC-P components. The *ko-toc33* mutant shows a defect in the accumulation of Toc159, but the *kd-toc159* mutant did not affect the accumulation of Toc33. We further addressed this concern by performing additional experiments using *kd-toc159* wherein we revealed the presence of an intermediate complex, TOC-iP, in which Toc75 interacts with Toc33 (please see also our second response to Reviewer #6 below for further details). The accumulation of this intermediate complex (Toc75-Toc33) indicated that Toc159 assembly very likely follows the assembly of Toc33. This analysis is now included in the revised manuscript in Fig. 5f-h.

Moreover, in this manuscript we also provided insights into the assembly of the TOC-N complex. For example, Fig. 2 shows that the *ko-toc34* mutant displays specific deficiencies in TOC-N components but not in TOC-P components, indicating the crucial function of Toc34 in the accumulation of the TOC-N complex. However, in the *ko-toc132* mutant the accumulation of Toc34 and Toc75 was not affected, pointing to a terminal role for Toc132 in the assembly of the TOC-N complex. Overall, after the initiation of TOC assembly by Toc75, the receptor components Toc33 and Toc34 act downstream in establishing the TOC-P and TOC-N complexes.

In conclusion, our analyses provide physiologically relevant insights into the assembly of not only the TOC-P complex, but also the TOC-N complex.

Minor Comments:
1. The age of the plants shown in Fig. 1b and Supplementary Fig. 2 should be indicated.

Response: We apologize for this oversight. Four-week-old plants were shown in Fig. 1b. This information is now provided in the figure legend of the revised manuscript. The plants in Supplementary Fig. 2 were 3 weeks old as indicated in the relevant figure legend.

2. The meaning of the 200%, 100%, and 50% values in Fig. 1e, Fig. 4b, and Supplementary Fig. 1 should be clarified. For example, in Fig. 1e, does “100%” for roots and etiolated seedlings refer to an equal amount by fresh weight, or by micrograms of extracted protein?

Response: These values indicate loadings according to fresh weight of tissue. This has been clarified in each of the figure legends in the revised manuscript. However, please note that Fig. 1e has been replaced with Fig. R2 (as noted earlier) where the loadings are based on equal Toc75.

3. For more accurate comparison of TOC protein abundance across tissues and mutants, internal controls such as actin or histone H3 (as used in Fig. 4) should also be included in Fig. 1e, Fig. 2a, and Supplementary Fig. 1.

Response: The issue of loading normalization in Fig. 1e and Supplementary Fig. 1 was thoroughly discussed above in our response to Major Comment 1 of this referee (see also Figs. R2 and R3).

With regard to Fig. 2a, we have included actin as an internal control in the revised manuscript, as suggested

4. The specific Rubisco subunit detected in Supplementary Fig. 1 should be identified (large or small subunit).

Response: The detected Rubisco subunit is RbcL. This is now clarified in the revision. See also our detailed discussion around Fig. R3 above.

5. The naming of HA-Toc75 transgenic lines should be consistent. In Fig. 1, the lines are labeled HA-Toc75 #2 and #3, while in the Methods section, Fig. 4, and Extended Data Fig. 9, they are referred to as Ox-HA-Toc75 #1 and #2. This inconsistency should be addressed. The specific HA-Toc75 line used for TOC-P purification for negative-stain EM (Fig. 2e, Extended Data Fig. 1), in vivo labeling (Fig. 5), and Extended Data Fig. 8 experiments should also be clearly stated.

Response: This inconsistency has now been corrected in the revision.

6. The number of cysteine and methionine residues in Toc75 is inconsistent: the main text (line 326) states 20, while the Figure 5 legend (line 573) lists 21. Additionally, for Toc159, the count of cysteine + methionine residues is 26 if the initiating methionine is included, not 25 as stated. This discrepancy should be clarified.

Response: This discrepancy has now been corrected in the revision.

7. In line 233, the authors state that the Toc75–Toc159 complex “resembles the hybrid β-barrel of a BamA–substrate complex (Extended Data Fig. 5f).” In addition to reference 32, the authors should cite Jin et al. (Cell, 2022, https://doi.org/10.1016/j.cell.2022.10.030), which was the first report of this structural resemblance.

Response: The Jin et al. paper is now cited at this point in the revision.

8. In line 234, the authors state: “A part of an embedded segment of Toc159 extends across this junction and underneath the Toc75-side of the β-barrel (Extended Data Fig. 5c,d). This overall arrangement of the hybrid β-barrel likely allows the protein import channel to expand and contract flexibly in order to accommodate (un)folded protein domains of different sizes.”

(1) In Extended Data Fig. 5c,d, the “embedded segment” of Toc159 should be shown in a distinct color to better differentiate it from other parts of Toc159. Also, note the inconsistency between the singular “segment” used in the text and the plural “segments” in the figure legend.

Response: Thank you for these suggestions. The embedded segment have now been shown in a different colour (black) to improve clarity. Also, the singular/plural inconsistency has been corrected in the revision (plural is now used).

(2) The rationale for the claim that this hybrid β-barrel arrangement allows flexibility in channel size needs clarification. According to the Chlamydomonas TOC-TIC structure, the two sides of the hybrid barrel are stabilized by strong hydrogen bonds and hydrophobic interactions. Are hybrid β-barrels generally known to be flexible or capable of expansion/contraction? If so, please cite references; otherwise, the authors should explain the basis for this hypothesis or rephrase accordingly.

Response: During the biogenesis of β-barrel outer membrane proteins, transient hybrid β-barrel structures can form between insertases (such as Sam50 in mitochondria and BamA in bacterial systems) and their substrate proteins (e.g., VDAC, Tom40, EspP)[9-11]. These short-lived hybrid barrel intermediates eventually enable the substrate to fold into a separated, mature β-barrel. This dynamic behaviour tends to support the view that hybrid β-barrels are structurally flexible.

In the AF3-predicted model of the TOC complex, a similar hybrid β-barrel is formed between Toc75 and Toc159. On one side of this hybrid barrel there is a β-seam that is stabilized a set of hydrogen bonds between β1 of Toc75 and β14 of Toc159. On the opposite side of the barrel, the β-strands curve inward forming a hydrophobic grove and are stabilized mainly by weaker van der Waals (VDW) interactions (see Fig. R6 below). Not just the β-sheets, also, the embedded segment II of Toc159 may play a role in the flexible interactions within the hybrid β-barrel. This asymmetry (strong hydrogen bonding on one side, weaker VDW interactions on the other) may confer the structural plasticity needed to accommodate the translocation of large precursor proteins. However, experimental structural data are still needed to validate this hypothesis and to fully understand the dynamic behaviour of the hybrid β-barrel.

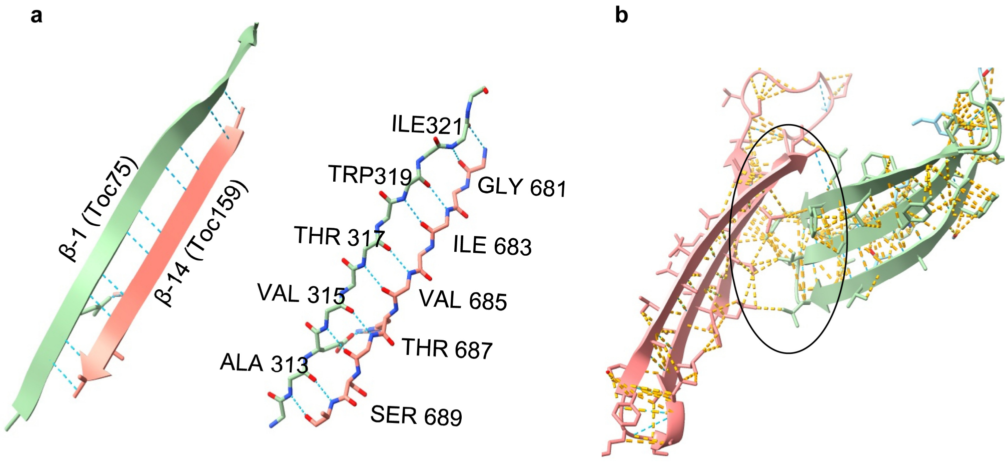

Fig. R6. *Stabilization of the hybrid Toc75-Toc159 β-barrel in the AlphaFold3 (AF3) TOC-P model.* ***a.*** *One side of the hybrid β-barrel is stabilized by a β-seam involving hydrogen bonding between β1 of Toc75 and β14 of Toc159. Only backbone hydrogen bonds are shown (blue).* ***b.*** *The opposite side of barrel, where β-strands curl inwardly, is stabilized primarily by weaker van der Waals (VDW) interactions (orange), with a VDW overlap of approximately −0.4 Å. The structure was analysed using Chimera.*

It is noteworthy that similar observations were made by Jin et al. concerning the TOC hybrid barrel in algae[12]. These structural observations are accordant with previous reports showing that the chloroplast translocon pore exceeds 25.6 Å in diameter and is sufficiently large to allow the translocation of folded proteins, with flexibility around the lateral gate potentially being important for this capability[13,14].

We have addressed this point briefly in the Results section of the revised manuscript (last paragraph on page 7, top of page 8), and added some additional references to support the point.

9. Typos:
Line 197: Should read "(Toc33-GTP2: KSS [aa 49–51])" instead of "[49–50]".
Line 927 and Fig. 5a: The light intensity unit should be "μmol m^{-2} s^{-1}," not "μmol m^{-2} s^{-2}."
Line 928: The concentration of Na_2SO_4 should be expressed in mM, not "Mm."

Response: All of these typos have been corrected in the revision.

Reviewer #2 (Remarks to the Author):

Nellaepalli and colleagues has combined various experimental approaches to demonstrate the presence of two distinct forms of protein translocon at the outer envelope of chloroplast, namely TOC-P and TOC-N, which participate in import of different types of preproteins, namely photosynthetic and non-photosynthetic proteins, respectively. While the receptor GTPases included in TOC-P and TOC-N are different from each other, the central component TOC75 is commonly used and appear to be a key component for the assembly of these two distinct TOC isoforms. Thus study already contains enormous amounts of experimental data, which might be acceptable for publication without any revision. However, there still seems to be some concerns that the authors might need to address or reconsider.

Response: We thank the reviewer for this positive feedback and constructive commentary.

The manuscript results section contains, as a considerably large part, AF3-predicted TOC structures and discussions based on this prediction. Although the authors have tried to add several experimental as well as MD data to support this prediction. However, the prediction still remains as a prediction. So, some of the descriptions should be transferred to discussion section or to supplementary discussion.

Response: We have with this revision substantially expanded the experimental verification of the initial structural prediction, and so believe that what we have now is an integrative structural analysis. Nonetheless, we accept that more could have been done to streamline the presentation, and so we took the referee's advice and transferred some of the analysis of the AF data to a new Supplementary Results section. The text on the properties of the

GTPase heterodimer are now shown in the Supplementary Results, alongside information on unique features of the *Chlamydomonas* receptors.

In relation to this notion, they describe in the text that “Comparing the different structures, we found that TOC-P from A. thaliana resembles the cryo-EM and AF3-predictedstructures of the algal TOC complex, indicating that structural organization of the TOC complex is broadly conserved in plants and green algae”. This seems to be a bit peculiar. While this reviewer is not sure when the authors actually performed this AF3 prediction, this was carried out probably after Chlamydomonas TOC-TIC supercomplex cryoEM structures had been available in public. This means AF3 predicted the TOC-P and TOC-N structures based on the deposited experimentally determined Chlamydomonas TOC structure as a kind of template as well as some typical GTP-binding proteins for GTPase domains of TOC receptors which were invisible for the Chlamydomonas TOC.

Response: Please refer to our response to the similar point made by Reviewer #1 (their Major Comment 6).

If my memory is correct, the reported Chlamydomonas TOC contains several previously unidentified TOC components associated with the TOC core and most strikingly the peculiarly integrated protein strands derived from the largest component of TIC, namely Tic214. However, the present discussion or description in Results section has not at all any mention for those. This reviewer would like to know how the spaces in the AF3 predicted structure corresponding to those occupied by Tic214 strands or by other TOC components are filled with what(?) or remain completely empty. More detailed structural comparison between the Chlamydomonas entire TOC and the AF3 predicted TOC with only TOC75 and two GTPase receptors.

Response: Thank you for raising these intriguing issues. In *Chlamydomonas*, as noted, a small segment of Tic214 interacts with and passes into the TOC hybrid β-barrel. In our AF3-predicted Arabidopsis TOC models, the corresponding region of the TOC channel is vacant. This point is now mentioned in the revised manuscript (Results, last paragraph on page 8). We did attempt to model whether Arabidopsis Tic214 might interact in this region using AF3, but in these analyses the predicted models had very poor confidence and the results were inconclusive.

In addition, we have provided a more comprehensive comparison with the algal machinery, as requested. There is now a whole Results section dedicated to this point (entitled “*Conserved and distinct features of the TOC complex in plants and algae*”) on pages 8-9; and we have included additional information in the Supplementary Results which covers the points below.

Notably, the *Chlamydomonas* TOC GTPase domains which are missing in the cryo-EM structures exhibits two distinct characteristics: (i) Toc34 features an additional, short N-terminal region (1-59 aa, pI = 3.38) that is acidic in nature (Fig. R7 and Supplementary Fig. 7d,e in the revised manuscript). (ii) Conversely, the N-terminus of Toc90 is basic in nature (189 aa, pI = 10.9). In contrast, land plants lack an acidic domain in Toc33/-34 but possess large N-terminal domains in the Toc159 isoforms that are acidic in nature (for Toc159, 781 aa, pI = 3.98). However, functional analyses are required to better understand the particular properties of the algal TOC complex.

The *Chlamydomonas* TOC complex[12] is composed of three conserved TOC components (Toc75, Toc90 and Toc34) as well as three algal-specific subunits (Toc52, Toc39 and Toc10). Notably, Toc52 and Toc39 form a distinct TOC complex which is unique to algae. In our AF3 analysis, all of the conserved components (i.e.,Toc75, Toc159-family and Toc33-family) were included.

a

```
TOC34_CHLRE (A8HYJ1)     MAQPPRPAEEYDDDVQEDEDELKEGELDDDESHEAASEGGEAAAGDEEAEDDEQDEEDGD    60
TOC33_ARATH (O23680)     ----------------------------------------------------------MG    2
                                                                                    .
TOC34_CHLRE (A8HYJ1)     EDSQPWAGLNRLPER--DDMLDILNELRAEGRKQLTVLLLGKSSVGKSSLINSLLGEAVV    118
TOC33_ARATH (O23680)     SLVREWVGFQQFPAATQEKLIEFFGKLKQKDMNSMTVLVLGKGGVGKSSTVNSLIGEQVV    62
                         .  : *.*::::*    :.:::::.:*: :. :.:***:***..***** :***:** **
TOC34_CHLRE (A8HYJ1)     RVQAFKLQADTDITTTVVRQVAVGNSEVDGFRLKLIDTCGLEDPEAGDTVNLGALSKIAE    178
TOC33_ARATH (O23680)     RVSPFQAEGLRPV---------MVSRTMGGFTINIIDTPGLVEAGY---VNHQALELIKG    110
                         **. *: :.   :         : .  :.** :::*** ** :      **  **. *
TOC34_CHLRE (A8HYJ1)     DVRGVGIDVVLYCDRLDLYRVDPLDKAIIDAISSTFGRGIWRRTVVALTHANLVQTPPGT    238
TOC33_ARATH (O23680)     FLVNRTIDVLLYVDRLDVYRVDELDKQVVIAITQTFGKEIWCKTLLVLTHAQFS-PPDEL    169
                         : .   ***:** ****:**** *** :: **:.***: ** :*::.****::   *
TOC34_CHLRE (A8HYJ1)     DYDSFVNGRVRLIRGA------VRGPLFFRPSLPVALVENSETCPVSSESGFRVLPDGEP    292
TOC33_ARATH (O23680)    SYETFSSKRSDSLLKTIRAGSKMRKQEFEDSAIAVVYAENSGRCSKN-DKDEKALPNGEA     228
                         .*::* . *   :  :      :*   *   :: *. .***  *   . :.. :.**:**
TOC34_CHLRE (A8HYJ1)     WLVALVSQLVDMAAARRRPYKYHPRLSSK--PSHRFRWLLPVAIAAEVLFYRRFLHPRLD    350
TOC33_ARATH (O23680)     WIPNLVKAITDVATNQRKAIHVDKKMVDGSYSDDKGKKLIPLIIGAQYLIVKMIQG-AIR    287
                         *:  **. :.*:*: :*:  : . :: .    ..: : *:*: *.*: *: : :    :
TOC34_CHLRE (A8HYJ1)     DNQRRVEREEERVWALRGQQRRALGLHRPHRPDKDAAWRLEQMYDDD 397
TOC33_ARATH (O23680)     NDIKTSGK---PL---------------------------------- 297
                         :: :   :    :
```

b

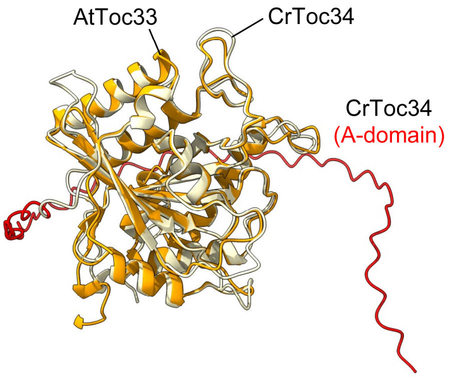

***Fig. R7.** Structural differences and similarities between the plant and algal TOC receptors. **a.** A multiple sequence alignment of Arabidopsis Toc33 (AtToc33) and Chlamydomonas Toc34 (CrToc34) reveals that the N-terminus of CrToc34 (residues 1-59) is notably enriched in acidic residues. The calculated isoelectric points (pI) for AtToc33 and CrToc34 are 9.11 and 4.68, respectively, highlighting the significant differences in their charge properties. **b.** Superposition of the GTPase domains of AF3-generated models of AtToc33 and CrToc34 shows an RMSD of 0.805 Å, indicating a high degree of structural similarity between the two proteins despite their differing N-terminal characteristics.*

Additional obvious lack of this study may be MS investigation of purified TOC75 fractions, TOC159 fraction, and TOC33, fraction, which will tell us whether the fractions only contain TOC75 and two TOC GTP receptors proteins as the authors have proposed entirely in this study or contain any other proteins which might have been missing in their AF3 predictions.

Response: Thank you for this important comment. Mass spectrometry analysis of our HA-Toc75 preparation was already discussed in our first response to Reviewer #1 above (see Fig. R1); are these data are now thoroughly presented in the revised manuscript (see Extended Data Fig. 1 and Supplementary Table 1).

Polypeptides were separated by SDS-PAGE, and abundant proteins (based on staining) were excised from the gel and were identified by mass spectrometry; in addition, the same samples were analysed in-solution prior to electrophoresis (Supplementary Table 1). Moreover, we conducted a quantitative mass-spectrometry analysis of similar samples (Extended Data Fig. 9d). Overall, the data revealed clear enrichment of the core TOC-P components, Toc75, Toc159 and Toc33, with TIC components and assembly factors such as OEP80 identified only at low levels. Thus, we are confident that it was reasonable to predict the TOC-P structure using just the most abundant proteins.

The predicted close contact between the two GTPase domains of receptors is interesting. But again, this still seems to be as a predicted interaction. Although they performed XL-MS of the purified complexes, have they identified actual XL peptide(s) derived from both GTPase domains?

Response: Thank you for this important comment. As we have outlined in our detailed reply to Reviewer #4 below, we have performed additional XL-MS experiments resulting in the identification of 14 new crosslinks in addition to the previously detected crosslinks. However, all of the new crosslinks are either intra-domain or inter-domain but not inter-subunit. To understand the reason for this, we mapped possible crosslink sites relative to the subunit interfaces within the TOC-P complex: relative to all possible K-K crosslink sites in the complex within a 10-30 Å range, only one fifth lie at subunit interfaces. This indicated that identification of inter-subunit crosslinks is predicted to be challenging using BS3.

Thus, we took a different approach, and addressed the specific question raised here about the TOC GTPase dimer by conducting Co-IP assays. Using a tobacco transient expression system, we were able to successfully validate the formation of the GTPase heterodimer in vivo (see Fig. R8 below, and Fig. 3h in the revised manuscript). In this analysis, the two GTPase domains were tagged (Toc33G-Flag, Toc159G-HA) to enable purification and detection. The detected physical interaction between the two GTPases in planta is consistent with and supportive of the AF3 prediction.

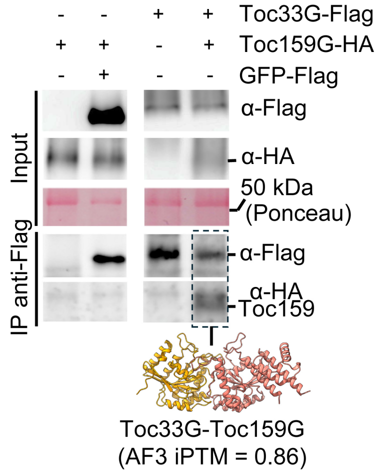

Fig. R8. *Co-immunoprecipitation analysis of the Toc159-Toc33 interaction in planta. Toc159G-HA and Toc33G-Flag were transiently expressed in Nicotiana benthamiana. Anti-Flag immunoprecipitated proteins were analysed by immunoblotting using anti-Flag (α-Flag) and anti-HA (α-HA) antibodies. GFP-Flag was used as a negative control.*

Minor points:

Why their roots fraction contains visible amount of Rubisco?

Response: We believe that this remark refers to the previous Ponceau data in Fig. 1e, which we agree were not sufficiently clear. Importantly, these samples have now been re-analysed based on the comments of Reviewer #1. We now have CBB staining data which are much clearer and show that RbcL is undetectable in root samples (see Fig. R2 above). These new data are now included in Fig. 1e of the revision.

The TOC and TIC may be separately purified from Arabidopsis. But there seems to be some TIC component bands in the immunoblots of purified TOC fractions. Have they tried different conditions, e.g. different detergents, for TOC-TIC supercomplex purification?

Response: Please refer to our detailed response to a similar comment from Reviewer #1 above (Major Comment 7).

Reviewer #3 (Remarks to the Author):

I co-reviewed this manuscript with one of the reviewers who provided the listed reports. This is part of the Nature Communications initiative to facilitate training in peer review and to provide appropriate recognition for Early Career Researchers who co-review manuscripts.

Response: We are grateful to this reviewer for helping to provide such valuable and constructive comments on our work.

Reviewer #4 (Remarks to the Author):

In this manuscript, Nellaepalli et al. report the assembly of TOC complexes from photosynthetic chloroplasts and non-photosynthetic plastids. The authors revealed that TOC75 is the key component of TOC complexes and acts as the driving force behind its assembly. By integrated cross-linking, genetic, and in vivo labeling approaches, they successfully characterized structure of TOC-P. They also showed that TOC-N shares similar overall structure of TOC-P while exhibiting distinct differences. My comments primarily focused on the cross-linking experiments as below.

Response: Once again, we are grateful for the constructive feedback on our work.

1.The authors only identified intra-protein crosslinks. The identification of inter-protein cross-links would significantly enhance the structural characterization of the TOC complex. The use of enrichable cross-linkers could facilitate the detection of such inter-protein interactions (Tan D. et al., eLife, 2016; Jiang P.L. et al., Angewandte Chemie, 2021).

Response: We sincerely thank the reviewer for this helpful comment. In response, we repeated the cross-linking mass spectrometry analysis using a modified approach. This time, we attempted to enrich for inter-protein crosslinks by using an in-gel extraction method after cross-linking. In brief, purified TOC samples were crosslinked using 0.5 mM BS3 for 30 min; the samples were separated via SDS-PAGE; the crosslinked TOC proteins were extracted from the gel (high-molecular weight smears [above 250 kDa]); and then samples were analysed by mass spectrometry to identify potentially new crosslinked peptides. In this new analysis, we obtained 14 new crosslinks in addition to the 7 previously characterized crosslinks (see Fig. R9 below). All of these cross-links could be mapped onto our structural model representing each of the three subunits (Toc75, Toc159 and Toc33) integral to the TOC-P complex. While inter-subunit peptides were unidentified in our experiments, we think the following two reasons are the likely cause:

1. The TOC complex is inherently flexible and this property is likely enhanced in the absence of a lipid bilayer as is the case in our purified samples used for cross-linking mass spectrometry. The three-domain architecture of the complex (cytosol-exposed GTPase dimer [receptor module]; transmembrane hybrid beta-barrel [channel module]; intermembrane space facing POTRA domain) involves interfaces on both sides of the lipid membrane, and in its absence the loose inter-subunit contacts between, e.g., the receptor and channel modules of the TOC complex, inferred from our structural analyses and MD-simulations, will be potentially limited.

2. On mapping lysine residues at the interface of the subunits in the TOC structural model, the lysines were found to be either less abundant or potentially spatially restricted within the relevant 10-30 Å distance window, indicating that identification of inter-subunit crosslinks would be challenging using BS3 alone.

Unfortunately, we are not experienced in implementing the Leiker cross-linking approach to enrich inter-protein interactions as suggested by the reviewer. However, we understand its strength for identifying interlinks and we will standardize and implement this in our future experiments. Nevertheless, our now substantially expanded cross-linking mass spectrometry dataset (alongside additional experimental verifications from our electron microscopy and co-IP analyses) already gives us strong confidence in our TOC structural model. Moreover, the XL-MS data provides reassurance that we can purify the intact TOC complex and that the complex is properly folded. Importantly, our 2D images of the purified TOC-P complex, obtained by electron microscopy using negative-stain, align well with the structure prediction from AlphaFold (please see the new Fig. 3e); and we verified the predicted inter-subunit interactions in the receptor module by conducting co-IP assays using an in planta expression system (please refer our response to the final Major Comment of Reviewer #2 and to Fig. R8 above).

No	Cross-links	TOC subunits	Linkage mapping	Linkage cites	Cα–Cα distance
1	SLMGSVTTSNFLNPQDDLSFKLEYVHPYLDGVYNPR-AGVKANITENFTR	Toc75	β[3-4]-6β	373-426	11.263Å
2	AGVKANITENFTR-DVKGNPTAVYR	Toc75	6β-L-β[13-14]	426-630	26.766Å
3	ELETLATCGMFEKVDLEGK-DQGKVSAR	Toc75	POTRA1-POTRA2	84-179	19.353Å
4	KALSNINSLGLFSNIEVNPRPDEK-DNTKFVNGAVVGQR	Toc75	POTRA3-L-β[8-9]	271-500	15.927Å
5	AGVKANLTENFTR–TFKTSCFNSR	Toc75	6β-5β	393-426	8.129Å
6	DNTKFVNGAVVGQR–HQLTMTKFLQLR	Toc75	10β-L-β(8-9)	500-537	11.709Å
7	HQLTMTKFIQLR-DNTKFVNGAVVGQR	Toc75	L-β(8-9)-10β	537-500	11.709Å
8	TVFQVDQGLGLGSKFPFFNR–DVKGNPTAVYR	Toc75	L-β[13-14]-L-β[9-10]	524-630	14.320Å
9	DNTKFVNGAVVGQR–ELPKQLR	Toc75	POTRA3-L-β[8-9]	256-500	21.175Å
10	KALSNINSLGLFSNIEVNPRPDEK-ELPKQLR	Toc75	POTRA3-POTRA3	256-271	10.645Å
11	KLSPVFTGGPGVEEVPPIWVDR-AGVKANITENFTR	Toc75	5β-6β	401-426	17.613Å
12	QSKFTYGLVMEEITTR(3)-AGVKANITENFTR	Toc75	7β-6β	426-438	17.614Å
13	TGQGSSYGAGVKLGLVR-DVKGNPTAVYR	Toc75	14β-L-β[13-14]	630-651	40.051Å
14	AGLNNKMSGQLTVR–NSKLALR	Toc159	L-β[13-14]-14β	668-678	31.369Å
15	AGLNNKMSGQLTVR–KVFGFR	Toc159	L-β[13-14]-Linker2	307-678	39.88Å
16	SIRPEATNDKYSMY-KVFGFR	Toc159	C-termi.-Linker2	307-719	52.266Å
17	SNIFSNSNVTMADETEINLSEEEKQK-LEKLQSLRVKFLR	Toc159	α*-α*	4-16	18.397Å
18	SNIFSNSNVTMADETEINLSEEEKQK-LEKLQSLRVKFLR	Toc159	α*-α*	4-9	8.679Å
19	SNIFSNSNVTMADETEINLSEEEKQKLEK-LQSLRVKFLR	Toc159	α*-α*	6-16	15.389Å
20	NSKLALR–KVFGFR	Toc159	14β-Linker2	307-668	55.85Å
21	KQEFEDSAIAVVYAENSGR-SDSLLKTIR	Toc33	α5'-α5	184-194	10.289Å

Fig. R9. *Identification of crosslinked peptides by mass spectrometry. Affinity-purified HA-Toc75 samples were crosslinked using 0.5 mM BS3 for 30 minutes. Crosslinked samples were separated by SDS-PAGE, and the high-molecular weight smears (>250 kDa) were excised from the gel. The resulting samples were digested with trypsin for proteomic analysis. The crosslinks highlighted in blue here are new intra-subunit crosslinks identified in this preparation.*

2.Integrating cross-linking data with AlphaFold predictions has the potential to improve the accuracy of TOC complex structure modeling (Stahl K. et al., Nature Communications, 2024; Stahl K. et al., Nature Biotechnology, 2023). Such a combination is recommended to strengthen structural insights.

Response: Thank you for this valuable suggestion. As requested, we have now implemented AlphaLink2 analysis (see Fig. 10 below, as well as Fig. 1g and Extended Data Fig. 3 in the revised manuscript). This approach enables integration of crosslinking mass spectrometry data directly into AlphaFold-Multimer by extending Uni-Fold[15,16]. For the AlphaLink2 predictions, FASTA files of the TOC subunits and a CSV file containing TOC amino-acid coordinates derived from the crosslinking data and false discovery rates (residueFrom, chain1, residueTo, chain2, FDR) were submitted to the AlphaLink2 Notebook. AlphaLink2 predicted the TOC-P structure (AL-TOC-P) with good confidence (iPTM = 0.71, crosslink satisfaction = 0.79), with most crosslinks falling within the predicted distance limits (see blue circles in Fig. 10). A small number of crosslinks exceeded these limits (red circles) and were located in flexible regions of the TOC complex, including Linker-1 and the C-terminus of Toc159, as well as the cytosolic loop of Toc75. It is likely that these dynamic regions were not captured by a single static model. As AlphaLink2 is based on AlphaFold-Multimer, a direct comparison between the AL-TOC-P and AF3-TOC-P predictions is not ideal; however, such an analysis did reveal a very similar structure for TOC-P suggesting that the predictions from AF3 are in agreement with experimental data (the RMSD between the two core structures was 0.937 Å).

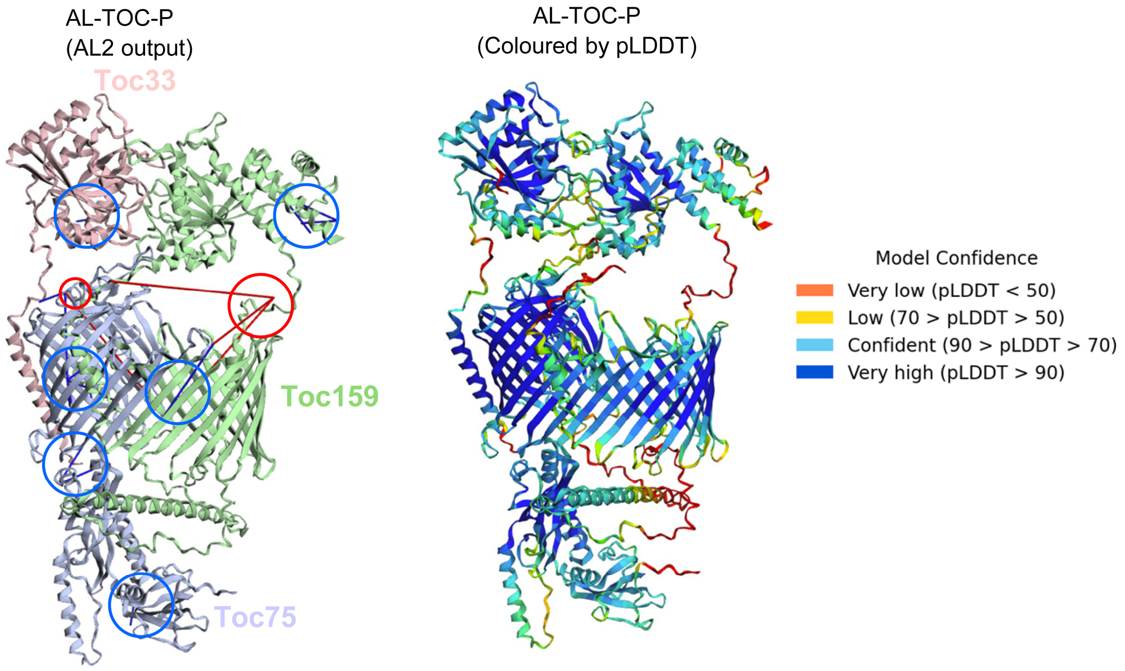

Fig. R10. *AlphaLink2 predication of the TOC-P complex. The AL-TOC-P structure is coloured according to polypeptide identity (left) or pLDDT scores (right). Blue and red circles indicate crosslinks that are satisfied or not satisfied by the Alphalink2 model, respectively. AlphaLink2 predictions satisfied the majority of the experimentally-detected crosslinks (in total 17) suggesting good agreement between experimental data and predicted structure. A few crosslinks (in total 4) were located in flexible regions of the TOC complex and so could not be captured by single static model (Cα-Cα distances of 35-55Å). Overall AL-TOC-P model confidence, 0.71; pTM, 0.72; ipTM, 0.70; crosslink satisfaction, 0.79; mean distance of crosslinked residues, 23.35 Å.*

3.In Supplementary Fig. 8, the labeling styles of the mass spectra are inconsistent. Standardizing the labeling format across all spectra would improve clarity and readability.

Response: The differences in the labelling formats arises from the distinct output styles of pLink3 and MetaMorpheus. Unfortunately, the output files are in PDF format making it difficult to edit the spectra it to a more consistent. Nonetheless, all of these raw data have now been enlarged slightly to improve their clarity and readability.

4.For cross-link #4 in Supplementary Fig. 8, the number of assigned peaks is limited, particularly for the beta chain of the cross-linked peptides. Generally, the identification of cross-linked peptides is evaluated based on the following criteria: (1) Continuous fragment ions should be assigned to both the alpha and beta chains of the cross-linked peptides; (2) All major/strong peaks in the spectrum should be matched to the cross-linked peptides; (3) The mass spectrum should exhibit a high signal-to-noise ratio. Based on these standards, the identification quality of cross-link #4 appears to be low.

Response: We thank the reviewer for this important comment. We agree that the beta chain of crosslink #4 had a limited number of assigned peaks. Fortunately, in our new crosslinking mass spectrometry analysis, we identified this crosslink again (#9), this time with continuous fragmentation for both the alpha and beta chains. Thus, we are now confident that this identification is of good quality.

5.According to my knowledge, more cross-linked peptides should be identified for TOC complexes. Maybe, the authors could optimize their cross-linking procedures.

Response: Please refer to our response to Comment 1 of this reviewer above.

6.In page 41, line1000, the authors describe their cross-linking conditions: “The HA-purified TOC complex was crosslinked with 3 mM bis(sulfosuccinimidyl)suberate (BS3) (Thermo Scientific) at room temperature for 3 h”. Typically, cross-linking reactions with BS3 are performed using 1 mM BS3 for 1 hour. The crosslinking conditions used here may lead to over-crosslinking, potentially resulting in non-specific cross-link identifications.

Response: Please refer to our response to the Comment 1 of this reviewer above.

7.For cross-linking mass spectrometry experiments, have the authors performed technical replicates or biological replicates? Replicates are essential to validate data reproducibilityand ensure overall data quality.

Response: We have performed technical replicates and three biological replicates. The listed crosslinks are a sum of the crosslinks identified in all the replicates.

8.Depositing mass spectrometry data into public database (such as proteomeXchange) is encouraged

Response: We have already deposited our data in the proteomeXchange database (dataset identifier: PXD064453). All of the additional data generated during the revision process will be deposited in a similar way.

9.It is good to label the Cα-Cα distances of crosslinking sites on Fig. 3g.

Response: Thank you for this helpful suggestion. We have indicated the Cα-Cα distances as requested.

10.Supplementary Table 1 should be expanded to include additional key information, such as scores and Cα-Cα distances

Response: We have indicated the scores (Supplementary Table 2) and Cα-Cα distances (Extended Data Fig 3 and Supplemental Figure 9) in the revised manuscript as requested.

11.“pLINK3” should be “pLink 3”.

Response: This was corrected in the revision.

12.“in vivo” should be “*in vivo*”.

Response: According to the Nature portfolio house style, we believe that in vivo should not be in italics.

Reviewer #5 (Remarks to the Author):

This study combines biochemical analysis with structural modeling to provide insights into the TOC-P and TOC-N complexes from Arabidopsis. While this study is certainly interesting, there are several concerns regarding the modeling results, given that they form a key component of this study. Specific comments are given below.

Response: We thank the reviewer for their constructive comments. We believe that we have addressed them all thoroughly, as detailed below, and that doing so has considerably improved the manuscript.

1. The authors note that the AF3 models for the TOC-P and TOC-N complexes are "high confidence" (lines 149-150, 177-178), but the ipTM scores are not high (0.72, 0.70). As noted by the AlphaFold developers on the AlphaFold3 server site (https://alphafoldserver.com/faq), "ipTM values between 0.6 and 0.8 are a gray zone where predictions could be correct or incorrect." The authors should adjust their language and descriptions to note that the models have moderate rather than high confidence scores.

Response: We thank the reviewer for raising this important point. It is important to clarify that the AF3 ipTM (interface predicted template modelling) score quantifies the accuracy of the entire structure of a complex, including all unstructured/no-fixed-structure elements; and in the case of TOC-P, ipTM is 0.72, which is lower than 0.8. Importantly, models predicted with ipTM > 0.8 are deemed high confidence and therefore like real structures.

As we demonstrated in our molecular dynamics (MD) simulations, the TOC-P complex has a high degree of flexibility especially at the interface between the GTPase heterodimer and the hybrid β-barrel which is comprised of mostly-disordered linkers (low local confidence pLDDT score; shown as orange-to-red in Fig. R11A below). This interface would be at the membrane plane, which AF is not aware of; and as AF is not aware of membrane positioning, it cannot accurately model relative orientation between transmembrane and other parts of a membrane protein or complex. Because the ipTM score measures accuracy of the predicted relative positions of subdomains in a complex, we note that the exclusion of long unstructured linkers (which negatively contribute to ipTM scores) can be very informative. Notably, AF3 returns structures of individual subcomplexes of TOC-P, specifically the GTPase heterodimer and the hybrid β-barrel, with high confidence scores of ipTM > 0.8 (or pTM = 0.77 for the POTRA domains) (Fig. 11B). We have included these new data in the revised manuscript (Extended Data Fig. 3), and mention them briefly in the Results (bottom of page 5) with a more detailed discussion in the Methods (page 47, second paragraph of section entitled "*Alphafold3 and AlphaLink2 analysis*").

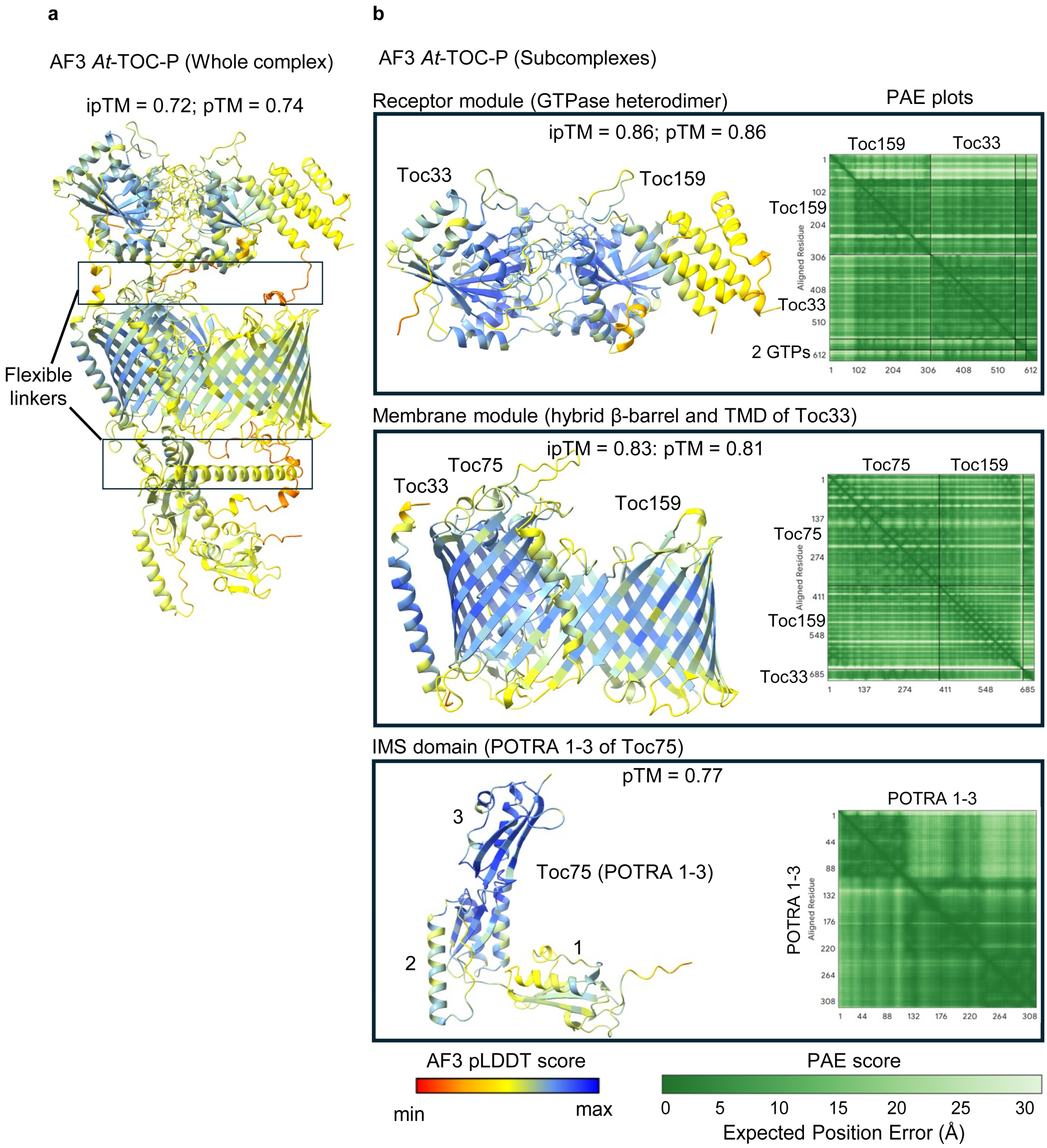

Fig. R11. *AF3 predictions of TOC-P and TOC-P subcomplexes.* ***a.*** *AF3 prediction of the whole TOC-P complex. Flexible regions that negatively influence the confidence scores are highlighted.* ***b.*** *From top to bottom: Toc33-Toc159 (GTPase heterodimer); membrane domain (Toc33-Toc75-Toc159); POTRA domain.*

2. Rather than or in addition to the PAE plot of the AF3 model shown in Fig. 3b, showing the modeled structure colored by pLDDT scores (b-factor of the model) would be helpful to show readers the local and residue-level confidence levels in the structural model. Such a structural representation should be added.

Response: Thank you for this suggestion. We have now added the TOC-P model coloured based on pLDDT scores as requested (Fig. 3c), in addition to PAE plot (Fig. 3b).

3. In Fig. 3j regarding the MD simulation results, it is suggested that the authors use units of Angstroms rather than nanometers, as that is a much more commonly used unit for protein structural analysis and would be more familiar to readers.

Response: We have replaced nm with Å as suggested in Fig. 3j,k and the related Methods section, as requested.

4. The rationale and interpretation of the Phyre2 homology model of Toc159 noted on lines 442-446 should be described by the authors more, at least briefly. The structural agreement between the identified template structure (Tom40) and the homology model of Toc159 seems to be a direct result of the homology modeling procedure, which by definition will generate models that will resemble the template. Thus, this does not seem to represent robust evidence of actual structural similarity between the proteins.

Response: Thank you for this comment. Of course, we agree that the Phyre2 model of Toc159 was based directly on the known Tom40 structure. However, we do think it noteworthy that Phyre2 identified Tom40 as the best-fitting structure for Toc159, although we agree that this observation is not conclusive. Please note that our structural comparison in Supplementary Fig. 8d (formerly Supplementary Fig. 7d) was not done using the Phyre2-generated model of Toc159, but rather using the AF3-generated model of Toc159. This further suggested that the two proteins share similar characteristics regarding channel formation, as the β-strands with short hairpin loops align closely.

5. While AF3 is a powerful modeling approach that can generate accurate models of monomers and multimers outside of its training set, one potential source of bias of the AF3 modeling is the use of template structures for individual chains, as well as PDB structures that have been seen during AF3 model training (structures before Sept. 30, 2021 PDB release date). This could in principle lead to high observed structural similarities with older PDB structures, for instance what is noted in Supplemental Figure 4. The authors should clearly note where the AF3 modeling is first described in the Results that the AF3 server uses PDB structural templates in modeling, along with the template date cutoff. These possible sources of bias in the models should also be noted in the Discussion.

Response: Thank you for this important comment. Please refer to our detailed response to the similar Major Comment 6 of Reviewer #1 earlier.

Reviewer #6 (Remarks to the Author):

This manuscript provides a detailed characterization of two distinct TOC complexes, TOC-P from photosynthetic chloroplasts and TOC-N from non-photosynthetic plastids, and identifies Toc75 as a central hub in TOC biogenesis. The integrative structural analysis and functional assays presented here advance our understanding of plastid protein import machinery. The work is timely, addresses an important knowledge gap, and is overall well-executed. However, several major points require clarification, additional data, or more rigorous interpretation before the manuscript can be accepted.

Response: We thank the reviewer for these encouraging and positive comments.

Major Comments
1. The structure shown in Fig. 3c suggests a very tight interaction between Toc75 and Toc159. This makes it puzzling why toc159 knockdown does not affect the protein level of Toc75 or Toc33. The authors should reconcile this apparent discrepancy, either with additional experimental data, literature context, or a discussion of potential compensatory mechanisms.

Response: This comment raises an intriguing point regarding the stability of a putative Toc75-Toc33 intermediate complex in the absence of Toc159, which we now refer to as TOC-iP.

To address this point, we first employed AF3 to predict the ability of Toc75 and Toc33 to interact and form the proposed intermediate complex. The TOC-iP complex was predicted with moderate confidence (ipTM = 0.71) and exhibited significant similarity with the Toc75-Toc33 part of TOC-P complex (RMSD between 303 pruned atom pairs is 0.62 Å). Interestingly, the model showed that the first four β-sheets of Toc75 fold inwards forming a semi-closed state in the absence of Toc159, rather than outwards to form an open state as in the hybrid β-barrel of the mature TOC-P complex. This suggests that Toc159 is required for the formation of the mature complex.

To further investigate the possibility that TOC-iP may be a stable intermediate, we analysed the interchain contacts responsible for stability of the Toc75-Toc33 complex using the computational energy function and the EvoEF2 tool[17]. The results showed that the total interchain energy for the Toc75-Toc33 complex is -63.8 kcal/mol, indicating that formation of the complex is thermodynamically favourable.

Next, to experimentally test the prediction of a Toc75-Toc33 intermediate complex, we conducted sucrose density gradient ultracentrifugation on total leaf protein extracts from the *kd-toc159* mutant (see Fig. R12 below). The observed co-fractionation of Toc75 and Toc33 in this genotype supports the idea that these components interact. Interestingly, both proteins appear at both the expected size range (fractions 9-12; around 300 kDa) and in heavier fractions (fractions 14-18; >500 kDa). We interpret that the heavier fractions may represent an intermediate complex (i.e., TOC-iP) that is stabilized by auxiliary factors during the assembly process.

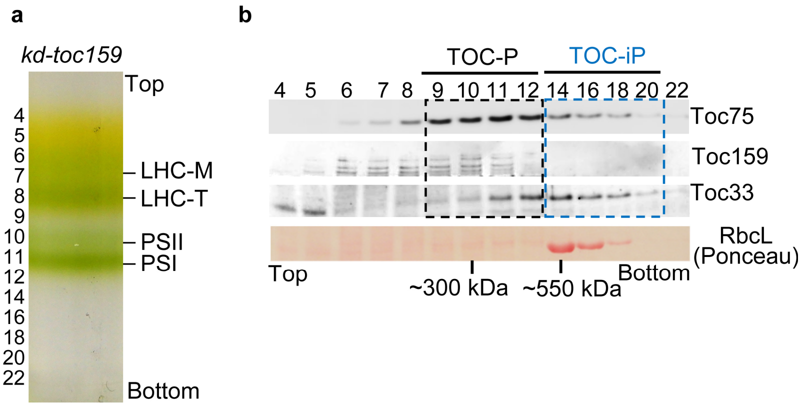

Fig. R12. *Separation of protein complexes in chloroplast-enriched leaf fractions of kd-toc159 mutant plants by sucrose density gradient ultracentrifugation.* ***a.*** *Image of the gradient showing*

the separation of pigment-protein complexes. ***b.*** *Fractions from each gradient were analysed by immunoblotting and Ponceau staining.*

Together, these new data provide evidence for the existence of the proposed intermediate complex, and so they have now been now included in the revised manuscript (Fig. 5 f-h), and are discussed in a new section of the Results entitled "*Identification of a TOC intermediate complex, TOC-iP*" (on pages 10-11).

Interestingly, Shanmugabalaji et al.[18] demonstrated that the integration of Toc159 into the chloroplast membrane is regulated by the phytohormone gibberellic acid (GA) and DELLA proteins during chloroplast biogenesis, with elevated GA levels facilitating Toc159 integration. This finding further supports the view that a Toc75-Toc33 intermediate complex may exist prior to the assembly of Toc159.

2. The authors believe that no experimental structure is provided for TOC-N due to flexibility seen in MD simulations. However, the reported flexibility does not appear particularly prominent. Please explain more clearly why structural characterization was not possible, was this due to sample heterogeneity, instability during purification, or technical limitations?

Response: We wonder if there may have been some misunderstanding here. As outlined also in our response to Comment 1 of Reviewer #5 above, there are inherent flexibilities in the TOC complex (and this applies to both TOC-P and TOC-N) that are problematic for experimental structure determination. This challenge explains the absence of TOC GTPase domain information in the published *Chlamydomonas* cryo-EM structures[12,19].

While we are of course working on different experimental approaches to overcome these obstacles, by using advanced sample preparation methods, these studies will take time and consequently are beyond the scope of the present study. Importantly, it is in this context of experimental challenges that the integrative structural analysis presented in the present manuscript shows its real power – allowing insights to be gained where more traditional, purely experimental approaches have so far been unable to deliver any information.

3. The integrative structural analysis is a strength of the paper, yet the specific key differences between TOC-P and TOC-N remain insufficiently clear. The authors should explicitly describe which structural features are unique to each complex and how these may relate to the observed functional differences.

Response: Thank you for this comment. We did observe some differences between the TOC-P and TOC-N complexes, which may contribute to their functional differences. Most notably, there is a difference in surface electrostatic potential between the two complexes. Notably, in the TOC-P complex, the negative potential on the upper surface of Toc33 and the inner surface of the Toc75 β-barrel is counterbalanced by positive/neutral (non-polar) potential on the upper surface of Toc159 and the inner surface of the Toc159 β-barrel (see Extended Data Fig. 4f). This configuration of TOC-P may facilitate the efficient routing of preproteins, helping to enable the heavy traffic associated with importing photosynthetic preproteins. In contrast, the TOC-N complex exhibits a weaker gradation of surface electrostatic potential, suggesting that a slower import process is better suited or adequate for housekeeping preproteins. We have added some discussion on these interesting points

in the revised manuscript, in the Results (in the section entitled "*AF3 analysis reveals a similar structure for TOC-N*", pages 6-7) and in the legend for Extended Data Fig. 4f.

4. In Fig. 3e, the single negative-stain EM 2D class shown for At-TOC-P is not convincing. Please provide a panel showing all 2D classes alongside a representative raw micrograph so readers can assess particle quality and sample heterogeneity.

Response: A typical negative-stain micrograph of the purified TOC-P complex is shown below (Fig. R13). While a degree of clustering of particles is apparent in the sample, we focused the 2D classification analysis on the single particles only (circled in black) in order to produce the representative 2D view shown in Fig. 3e (and Extended Data Fig. 3a), which in terms of size and overall shape are very similar to 2D projections of the AF-predicted TOC-P model. In response to the reviewer's comment and to make this analysis more convincing, we have now included multiple comparisons in Fig. 3e (with further comparisons in Extended Data Fig. 3a), all of which support the presented conclusion. We believe that this analysis presents unprecedented structural insights into the Arabidopsis TOC-P complex, and we are confident that our experimental 2D classification analysis supports the presented structural model.

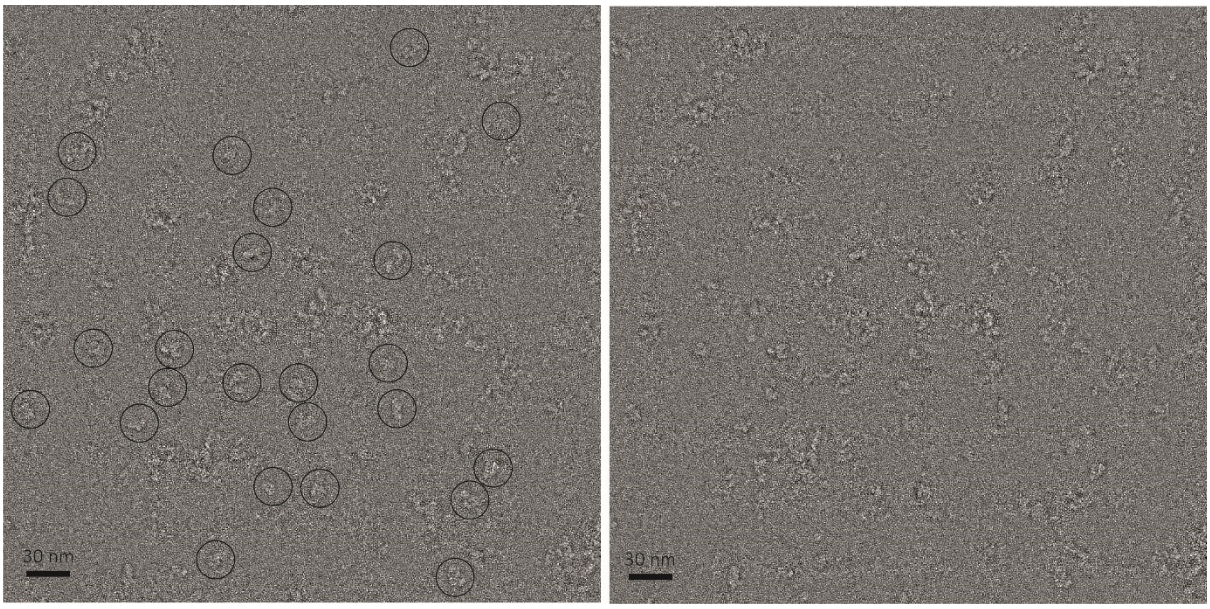

__Fig. R13.__ Negative-staining electron micrographs of the purified TOC-P complex. Samples were obtained after a two-step purification (affinity purification followed by size exclusion chromatography). Black circles show single particles selected for 2D classification analysis.

5. In Suppl. Fig. 2, toc159 knockdown phenotypes appear more prominent than those of toc75, despite the conclusion that Toc75 is the key player in complex stability. This discrepancy requires explanation.

Response: This is largely due to the differing strengths of the relevant mutant alleles, with the *kd-toc159* mutation being more strongly disruptive of protein function.

Specifically, in the *kd-toc159* mutant there is a nonsense mutation. The entire C-terminal alpha helix (32 amino acids) of Toc159 is deleted due to a premature stop codon (Q1472stop). We demonstrated that this helix is essential for the membrane stabilization of Toc159 (see Extended Data Fig. 8). As a result of this defect in membrane stabilization, only a very small amount of Toc159 is able to integrate into the membrane, thus significantly impairing chloroplast biogenesis and plant growth.

In contrast, the *kd-toc75* mutant carries only a missense mutation. This point mutation (G658R) does affect stability of the TOC complex, but the mutant retains all key TOC components, including Toc159, at substantial levels (see Fig. 2). Consequently, this mutant exhibits only mild phenotypic differences from wild-type plants.

6. Can Toc75 and Toc33 form a stable complex in the absence of Toc159? If experimental data are unavailable, have the authors attempted to predict such a structure using AlphaFold 3?

Response: Please refer to our detailed response to the closely-related Major Comment 1 of this reviewer above.

7. Fig. 2's immunoblotting results are clear. However, the conclusions could be strengthened by complementary immunofluorescence to confirm protein localization and abundance in situ.

Response: Thank you for the suggestion to conduct immunofluorescence experiments. While we accept that using this or a similar methodology could add further support to the conclusions arising from Fig. 2, we believe that doing this work would take 8-10 months to complete, given that the methodology is not currently established in our group. Therefore, given that the localization of TOC components is extremely well documented in the literature and not in any doubt, we feel that the proposed additional analysis here falls beyond the scope of the present study.

8. The microscopy-based ratiometric protein import assay in ko-toc33 and ko-toc34 mutants is elegant, but as it relies on protoplasts, the authors should discuss whether results may differ in whole tissue, given potential physiological differences.

Response: While we accept that there are certain limitations in conducting experiments using protoplasts (as indeed there are with any experimental system), we note that the protoplast system enables experiments that would otherwise be impossible owing to its flexibility and speed, and that it has been widely and successfully used to study many different aspects of plant biology including chloroplast protein import[20,21]. To address this comment, we summarized briefly the strengths and weaknesses of the system at beginning of the relevant section of the Results entitled "*Receptor mutant analyses reveal functional differences between TOC-P and TOC-N*" (on page 11).

Minor Comments

1. Line 149:"AF3 predicted the organization of the TOC-P complex with high confidence (ipTM = 0.72; pTM = 0.74)": An ipTM of 0.72 falls into the AlphaFold "gray zone" and indicates only moderate confidence in the predicted interface. Please acknowledge this

limitation and, if possible, provide supporting evidence from additional models or experiments.

Response: Please refer to our detailed response to Major Comment 6 of Reviewer #1.

2. Line 202: Regarding Toc33/Toc34 homodimerization: Is there any known physiological function for this homodimer in vivo? If unknown, please state explicitly.

10. **Response:** To our knowledge, the physiological role of Toc33 or Toc34 homodimers in vivo, if any, remains unclear. We have added clarification on this point to the revised manuscript (in the new Supplementary Results section; end of first paragraph).

3. Line 282: Toc75 expression parallels LHCB1 induction, whereas other TOC genes are only moderately upregulated. Could Toc75 have additional roles beyond its function in TOC-P and TOC-N complexes?

Response: This is an interesting question. However, we have not yet explored whether and how increased *TOC75* expression might influence other processes beyond the primary function in TOC complexes and protein import. Thus, we cannot rule out the possibility that higher levels of *TOC75* expression may help to promote chloroplast biogenesis in other less direct ways, for example through signalling.

4. Fig. 5b: It is unclear whether the purified chloroplasts shown are intact with outer membranes. Please provide a marker for the outer chloroplast membrane to confirm.

Response: We apologize for the lack of clarity in our presentation here. The data in Fig. 5b come from a radiolabelling experiment in which HA-Toc75 was affinity-purified from total protein extracts of young seedlings that had been incubated with ^{35}S-labeled Na_2SO_4; the samples were not extracted from isolated chloroplasts. This has been made clear in the relevant figure legend of the revised manuscript. Moreover, to demonstrate equal sample input and the presence of chloroplast proteins (notably, RbcL), CBB staining of the wild-type and HA-Toc75 extracts is now included in the revised version of the figure.

References

1. Ivanova, Y., Smith, M.D., Chen, K.H. & Schnell, D.J. Members of the Toc159 import receptor family represent distinct pathways for protein targeting to plastids. *Mol. Biol. Cell* **15**, 3379–3392 (2004).
2. Kubis, S. et al. Functional specialization amongst the Arabidopsis Toc159 family of chloroplast protein import receptors. *Plant Cell* **16**, 2059–2077 (2004).
3. Chu, C.C. & Li, H.M. Protein import into isolated pea root leucoplasts. *Front. Plant Sci.* **6**, 690 (2015).
4. Wallas, T.R., Smith, M.D., Sanchez-Nieto, S. & Schnell, D.J. The roles of Toc34 and Toc75 in targeting the Toc159 preprotein receptor to chloroplasts. *J. Biol. Chem.* **278**, 44289–97 (2003).
5. Hiltbrunner, A. et al. Targeting of an abundant cytosolic form of the protein import receptor at Toc159 to the outer chloroplast membrane. *J. Cell Biol.* **154**, 309–316 (2001).
6. Smith, M.D., Hiltbrunner, A., Kessler, F. & Schnell, D.J. The targeting of the atToc159 preprotein receptor to the chloroplast outer membrane is mediated by its GTPase domain and is regulated by GTP. *J. Cell Biol.* **159**, 833–843 (2002).
7. Abramson, J. et al. Accurate structure prediction of biomolecular interactions with AlphaFold 3. *Nature* **630**(2024).
8. Kikuchi, S. et al. Uncovering the Protein Translocon at the Chloroplast Inner Envelope Membrane. *Science* **339**, 571–574 (2013).

9. Takeda, H. et al. A multipoint guidance mechanism for beta-barrel folding on the SAM complex. *Nat. Struct. Mol. Biol.* **30**, 176–187 (2023).
10. Wu, R. et al. Plasticity within the barrel domain of BamA mediates a hybrid-barrel mechanism by BAM. *Nat. Commun.* **12**, 7131 (2021).
11. Shen, C. et al. Structural basis of BAM-mediated outer membrane beta-barrel protein assembly. *Nature* **617**, 185–193 (2023).
12. Jin, Z.Y. et al. Structure of a TOC-TIC supercomplex spanning two chloroplast envelope membranes. *Cell* **185**, 4788–4800 (2022).
13. Ganesan, I., Shi, L.X., Labs, M. & Theg, S.M. Evaluating the Functional Pore Size of Chloroplast TOC and TIC Protein Translocons: Import of Folded Proteins. *Plant Cell* **30**, 2161–2173 (2018).
14. Jeong, J., Moon, B., Hwang, I. & Lee, D.W. GREEN FLUORESCENT PROTEIN variants with enhanced folding are more efficiently imported into chloroplasts. *Plant Physiol* **190**, 238–249 (2022).
15. Stahl, K., Graziadei, A., Dau, T., Brock, O. & Rappsilber, J. Protein structure prediction with in-cell photo-crosslinking mass spectrometry and deep learning. *Nat. Biotechnol.* **41**, 1810–1819 (2023).
16. Stahl, K. et al. Modelling protein complexes with crosslinking mass spectrometry and deep learning. *Nat. Commun.* **15**, 7866 (2024).
17. Huang, X., Pearce, R. & Zhang, Y. EvoEF2: accurate and fast energy function for computational protein design. *Bioinformatics* **36**, 1135–1142 (2020).
18. Shanmugabalaji, V. et al. Chloroplast Biogenesis Controlled by DELLA-TOC159 Interaction in Early Plant Development. *Curr. Biol.* **28**, 2616–2623 e5 (2018).
19. Liu, H., Li, A., Rochaix, J.D. & Liu, Z. Architecture of chloroplast TOC-TIC translocon supercomplex. *Nature* **615**, 349–357 (2023).
20. Yoo, S.D., Cho, Y.H. & Sheen, J. Arabidopsis mesophyll protoplasts: a versatile cell system for transient gene expression analysis. *Nat. Protoc.* **2**, 1565–1572 (2007).
21. Lee, D.W. & Hwang, I. Transient expression and analysis of chloroplast proteins in Arabidopsis protoplasts. *Methods Mol. Biol.* **774**, 59–71 (2011).

Reviewer #1 (Remarks to the Author):

The authors have appropriately addressed most of my previous concerns, and the manuscript has been substantially improved. I have only a few remaining comments that can be addressed through rewriting.

Response: We sincerely thank the reviewer for these positive remarks on our revised manuscript. The remaining minor comments have now been addressed, as described below.

1. Concerning my previous major comment 7 (current manuscript, “Conserved and distinct features of the TOC complex in plants and algae,” line 247):
The authors cite a reference reporting purification of a TIC complex without TOC proteins (Kikuchi et al., 2013). I would like to remind the authors that these experiments were performed under extremely high salt conditions (1 M NaCl). Even considering this reference, there are substantial published data showing that TOC proteins can co-immunoprecipitate TIC proteins and vice versa, and that the amount of co-immunoprecipitated protein does not change in the presence or absence of bound precursor proteins. These findings suggest that stable TOC–TIC complexes can exist even in the absence of translocating precursor proteins (Akita et al., 1997, J. Cell Biol. 136:983–994; Nielsen et al., 1997, DOI: 10.1093/emboj/16.5.935; Kouranov et al., 1998, J. Cell Biol.).

Notably, the authors’ Fig. R5 (Extended Data Fig. 9a in the current revised manuscript) shows the presence of TOC proteins in fractions 14–18, which are the major fractions containing Tic214/56/100. This result could indicate that an independent TOC complex is present in fractions 9–12, while fractions 14–20 contain a TIC–TOC supercomplex, or a combination of a TIC–TOC supercomplex and a TIC complex. This interpretation is consistent with the results of Akita et al. (1997) and Chen and Li (2017; DOI: 10.1111/tpj.13643).

Response: We agree with the reviewer that a minor proportion of plant TOC and TIC complexes may exist as TOC-TIC supercomplexes in the absence of precursor protein. Thus, we revised the relevant text (on page 8) as follows:

“The results showed that TOC and TIC components primarily form distinct complexes of different sizes (~300 and ~600 kDa, respectively), suggesting that interactions between the TOC and TIC complexes in plants are weak or transient under these conditions, although minor co-fractionation of TOC components in the TIC-enriched heavier fractions (potentially representing TOC-TIC supercomplexes) was observed.”

In addition, we have cited all the suggested references in the following new sentence at the end of the same paragraph (page 9):

“Several studies based on chloroplast protein import assays previously suggested that proportions of plant TOC and TIC exist as TOC-TIC supercomplexes even in the absence translocating preprotein[37,40-42]; we speculate that these were preexisting interactions formed in planta, before organelle isolation, by chloroplasts that were actively engaged in natural import processes.”

It is possible that, when pull-downs are performed using a TOC protein, the TOC complex is preferentially isolated because most TOC proteins reside in the TOC complex, with only a smaller fraction present in the TIC–TOC supercomplex. Conversely, when pull-downs are

performed using a TIC component, as in the Chlamydomonas studies, the TIC–TOC supercomplex may be preferentially purified because a larger proportion of TIC proteins are present in that complex.

Response: We agree with this comment to some extent. However, more experiments would be required in order to reach a clear conclusion in this regard.

In this section of the Results ("Conserved and distinct features of the TOC complex in plants and algae," line 247) and based on Extended Data Fig. 9, the authors conclude that "TOC and TIC components form distinct complexes of different sizes (~300 and ~600 kDa, respectively), suggesting weak or transient interactions between the TOC and TIC complexes in plants." This conclusion is not supported by the data. The results show only that a greater proportion of TOC proteins is present in the ~300 kDa fractions and a smaller proportion in the ~600 kDa fractions. This is a matter of relative abundance and does not reflect interaction strength. Accordingly, this section should be re-written and Extended Data Fig. 9e should be removed.

Response: Thank you for this comment, which is closely related to points already addressed above. In the relevant figure (now Supplementary Fig. 14a), the enriched co-fractionation of TOC subunits in fractions 9-12 suggested that TOC exists as a complex in these fractions. Similarly, the enriched co-fractionation of TIC subunits in fractions 14-18 suggested that TIC exists as a complex in these fractions. However, if TOC and TIC complexes interact to form supercomplexes, intuitively one would expect them to co-fractionate in heavier sucrose fractions such as 20-22. Unfortunately, the immunoblotting signals were insufficient to enable clear detection of TOC-TIC components in these heavier fractions, suggesting that under these conditions the complexes do not interact strongly. In any case, to help resolve this matter, we have now additionally deleted Extended Data Fig. 9e (the model diagram where TOC-TIC interaction strengths were depicted), as requested.

2. Concerning my previous major comment 9:
The new data on TOC-iP are interesting. However, this reviewer still thinks that the review by Richardson et al. (2014; reference 17 in the current manuscript) should be cited when discussing Fig. 6 in the Discussion section. Citing this work would not diminish the novelty of the current study, but would appropriately acknowledge that a similar model has been proposed previously.

Response: We agree with the reviewer's suggestion. Accordingly, we have now cited this review article in the Discussion section (seventh line of paragraph 4).

3. Inconsistency in naming of HA-Toc75 transgenic lines:
The nomenclature used for the HA-Toc75 transgenic lines is inconsistent and should be corrected.
Line 328: "(HA-Toc75, #2 and #3)" versus line 333: "in the Ox-HA-Toc75 lines"
Figure 4d,e: "Ox-HA-Toc75 #1, #2" versus the Fig. 4d,e legend: "HA-Toc75 over-expressor plants"

Response: We thank the reviewer for noticing this inconsistency in naming the HA-Toc75 transgenic lines. We have now corrected this, both in the Results text (page 9) and in Fig. 7d,e (formerly Fig. 4d,e).

Reviewer #2 (Remarks to the Author):

Nellaepalli et al. now have extensively and adequately revised their previous manuscript with enormous amounts of additional high quality data in order to address most of the concerns raised by all the reviewers. I have no more concerns on this revised version. With these alterations, this manuscript will surely advance our understanding of assembly of TOC involved in chloroplast protein import.

Response: We thank the reviewer for these positive remarks, and for helping us to significantly improve the manuscript.

Reviewer #3 (Remarks to the Author):

I co-reviewed this manuscript with one of the reviewers who provided the listed reports. This is part of the Nature Communications initiative to facilitate training in peer review and to provide appropriate recognition for Early Career Researchers who co-review manuscripts.

Response: We thank the reviewer for co-reviewing our manuscript.

Reviewer #4 (Remarks to the Author):

Based on my knowledge, the cross-linked peptides are difficult to be extracted from SDS-PAGE gel,which often results in lower identification efficiency for in-gel digested cross-linking samples compared to in-solution digestion. I have no other comments.

Response: We thank the reviewer for this advice about how the extraction of crosslinked proteins from SDS-PAGE gels can be challenging, and for helping us to significantly improve the manuscript.

Reviewer #5 (Remarks to the Author):

The authors have addressed most comments satisfactorily. While it is helpful that the authors have now colored the models by pLDDT score in Extended Data Fig. 2 and elsewhere, the scale bar annotating the colors does not give any values at all and just notes "min" and "max" for the pLDDT range. Thus the pLDDT values could hypothetically range for instance from 20-50, or from 50-95, which would be a big difference. For Extended Data Fig. 3, in contrast, the pLDDT colors are more helpfully annotated. In Extended Data Fig. 2 and other figures with pLDDT color keys, the authors should instead note the pLDDT values for "min" and "max" for the model shown, or if they prefer, use a convention like in Extended Data Fig. 3. This would help readers to better understand both the pLDDT variability as well as the overall range of values being represented.

Response: We thank the reviewer for this important suggestion regarding the pLDDT scale bars. We have now revised the scale bars as requested (see below) in all relevant figures.

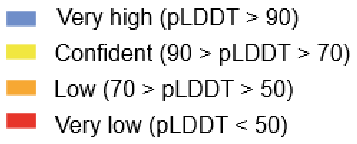

Reviewer #6 (Remarks to the Author):

The authors have adequately addressed all of my points, and I have no further comments or suggestions.

Response: We thank the reviewer for helping us to significantly improve the manuscript.
